# Discovery, isolation, heterologous expression and mode-of-action studies of the antibiotic polyketide tatiomicin from *Amycolatopsis* sp. DEM30355

**DOI:** 10.1038/s41598-022-18726-3

**Published:** 2022-09-16

**Authors:** Bernhard Kepplinger, Lina Mardiana, Joseph Cowell, Stephanie Morton-Laing, Yousef Dashti, Corinne Wills, Emma C. L. Marrs, John D. Perry, Joe Gray, Michael Goodfellow, Jeff Errington, Michael R. Probert, William Clegg, Jonathan Bogaerts, Wouter Herrebout, Nick E. E. Allenby, Michael J. Hall

**Affiliations:** 1 Biopharmaceutical Bioprocessing Technology Centre, Newcastle University, Newcastle upon Tyne, NE1 7RU UK; 2 Chemistry, School of Environmental and Natural Sciences, Newcastle University, Newcastle upon Tyne, NE1 7RU UK; 3 Department of Microbiology, Freeman Hospital, Newcastle upon Tyne, NE7 7DN UK; 4 Pinnacle Laboratory, Institute for Cell and Molecular Biosciences, Newcastle University, Newcastle Upon Tyne, NE2 4AX UK; 5 Biology, School of Environmental and Natural Sciences, Newcastle University, Newcastle upon Tyne, NE1 7RU UK; 6 Centre for Bacterial Cell Biology, Biosciences Institute, Newcastle University, Newcastle Upon Tyne, NE2 4AX UK; 7 Molecular Spectroscopy, Department of Chemistry, University of Antwerp, Groenenborgerlaan 171, 2020 Antwerp, Belgium; 8 Demuris Limited, The Biosphere, Draymans Way, Newcastle Helix, Newcastle upon Tyne, NE4 5BX UK; 9 Department of Molecular Microbiology, Faculty of Biotechnology, University of Wrocław, 50-383 Wrocław, Poland

**Keywords:** Chemical biology, Drug discovery, Microbiology

## Abstract

A genomic and bioactivity informed analysis of the metabolome of the extremophile *Amycolatopsis* sp. DEM30355 has allowed for the discovery and isolation of the polyketide antibiotic tatiomicin. Identification of the biosynthetic gene cluster was confirmed by heterologous expression in *Streptomyces coelicolor* M1152. Structural elucidation, including absolute stereochemical assignment, was performed using complementary crystallographic, spectroscopic and computational methods. Tatiomicin shows antibiotic activity against Gram-positive bacteria, including methicillin-resistant *Staphylococcus aureus* (MRSA). Cytological profiling experiments suggest a putative antibiotic mode-of-action, involving membrane depolarisation and chromosomal decondensation of the target bacteria.

## Introduction

Treatment of resistant infectious diseases poses a significant threat to human health, in particular those arising from the emergence of multidrug-resistant pathogenic bacteria. This has prompted significant efforts in the development of new small molecule antibiotics^1–4^, resulting in a resurgence of interest in natural product research to discover selective, novel mode-of-action therapeutic lead molecules^5–9^. Actinobacteria are a rich source of antibiotic natural products, with developments in this area being fuelled by the application of next-generation whole-genome sequencing technologies in combination with advanced bioinformatic analysis^10–14^. The combination of modern genomic techniques with the more classical approach of focussing on understudied, extremophilic, “unculturable” (so far not cultured) bacteria or rare bacterial genera, provides a rich ground for the discovery of new mode-of-action antibiotics. We therefore decided to investigate the metabolome of the extremophile *Amycolatopsis sp.* DEM30355^15,16^, which was isolated from a soil sample collected from the El Tatio geyser field within an arid part of the Atacama Desert in Chile^17^, through a combined genomic and bioactivity informed isolation approach resulting in the discovery of a novel anthracenone polyketide, tatiomicin. Following structural assignment, absolute stereochemistry was determined through a combination of crystallographic (resonant scattering), spectroscopic and computational methods, supporting the reassignment of the absolute stereochemistry of its nearest known natural product congeners the rishirilides^18^. Tatiomicin shows selective antibiotic activity against a focussed group of Gram-positive bacteria, including clinically relevant MRSA. Mode-of-action experiments indicated that tatiomicin may act as a Michael acceptor in vitro, an electrophile that can undergo conjugate addition reactions with biomolecules, and exhibits its antibiotic activity via a pathway involving membrane depolarisation and chromosomal decondensation.

## Results and discussion

**Figure 1:**
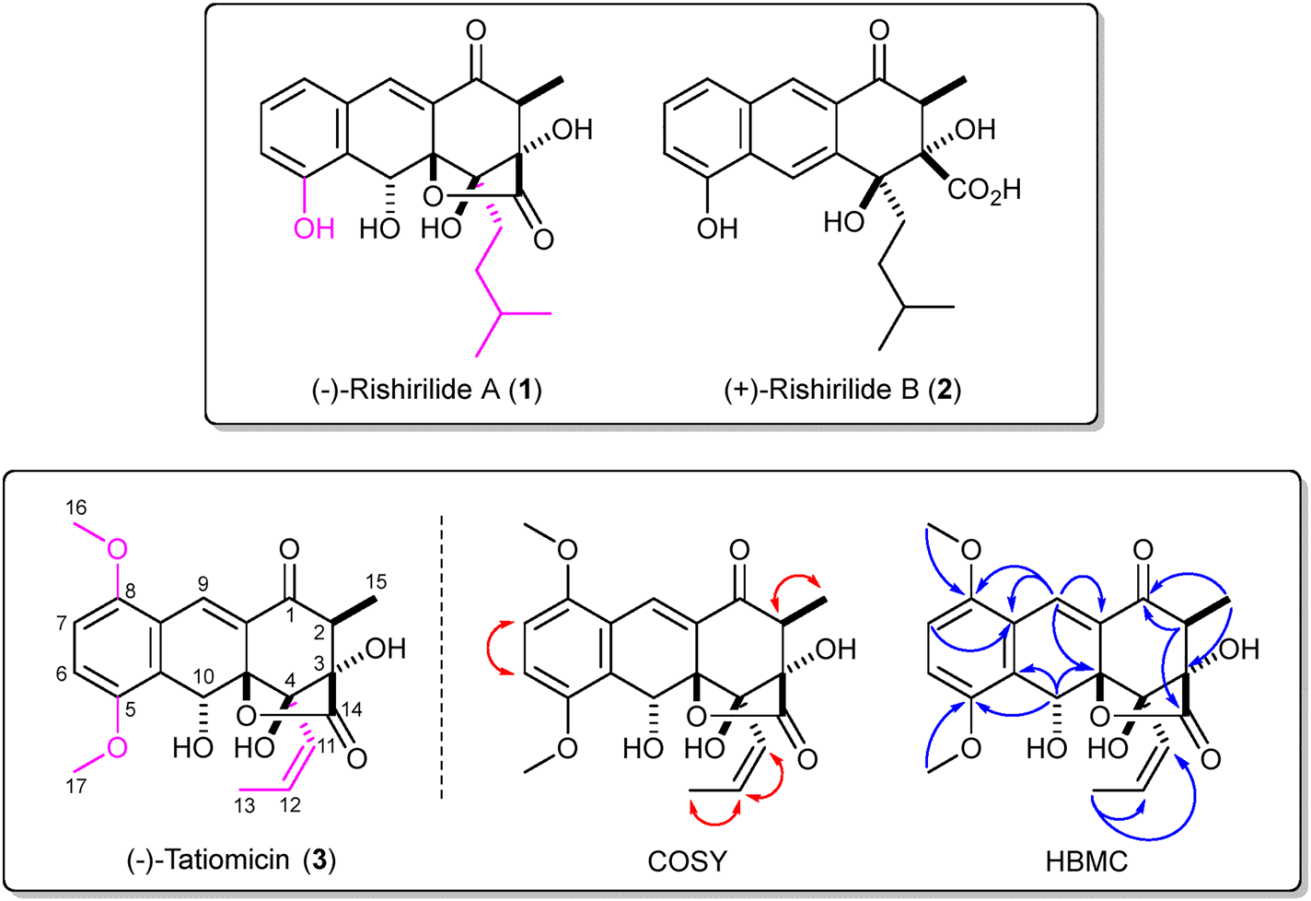
Top (–)-Rishirilide A (**1**) and ( +)-rishirilide B (**2**). Relative stereochemistry of (–)-1 and absolute stereochemistry of ( +)-2 shown. Bottom. Structure of (–)-tatiomicin (**3**) as derived from NMR and SCXRD experiments. Key COSY (red) and HMBC (blue) correlations shown. Absolute stereochemistry as shown by both vibrational circular dichroism (VCD) and single-crystal X-ray diffraction (SCXRD) resonant scattering experiments. Structural differences of rishirilide A and tatiomicin are highlighted (magenta).

The actinomycete DEM30355 was isolated from a soil sample, collected from the El Tatio geyser field within an arid part of the Atacama Desert in Chile^17^. Strain DEM30355 was recovered in the genus *Amycolatopsis*, based on 16S rRNA analysis, forming a subgroup with *Amycolatopsis vancoresmycina* DSM 44592^ T^ and *Amycolatopsis bullii* SF27^T^ (see ESI). The genus *Amycolatopsis* contains 94 species and four subspecies encompassing both extremophiles and producers of bioactive secondary metabolites, including the clinically used vancomycin and rifamycin antibiotics^19,20^. Preliminary bioactivity screening showed that extracts of *Amycolatopsis* sp. DEM30355 displayed promising antibiotic activity against *B. subtilis*, thus we decided to examine the genome of *Amycolatopsis* sp. DEM30355 for novel biosynthetic potential. Purified genomic DNA from *Amycolatopsis* sp. DEM30355 was analysed using both PacBio® and Illumina® sequencing technologies and genome assembly was performed using the combined datasets to give a 9.6 Mb draft genome, in 13 contigs. The draft genome of *Amycolatopsis* sp. DEM30355 was examined using the secondary metabolite analysis software AntiSMASH 6.0.1^21^. Of the 31 biosynthetic gene clusters (BGCs) detected, a PKS cluster was identified showing moderate overall similarity (81%) to that which encodes for rishirilides A and B^22–26^. These compounds are anthracenone polyketides, originally isolated from *Streptomyces rishiriensis* OFR-1056, with no reported antibiotic activity. Rishirilide B has been shown to be a moderately potent inhibitor of α2-macroglobulin, glutathione *S*-transferase and asparaginyl-*t*RNA synthetase, whilst little is known about the biological role of rishirilide A (Fig. 1)^18,27,28^.

**Figure 2:**
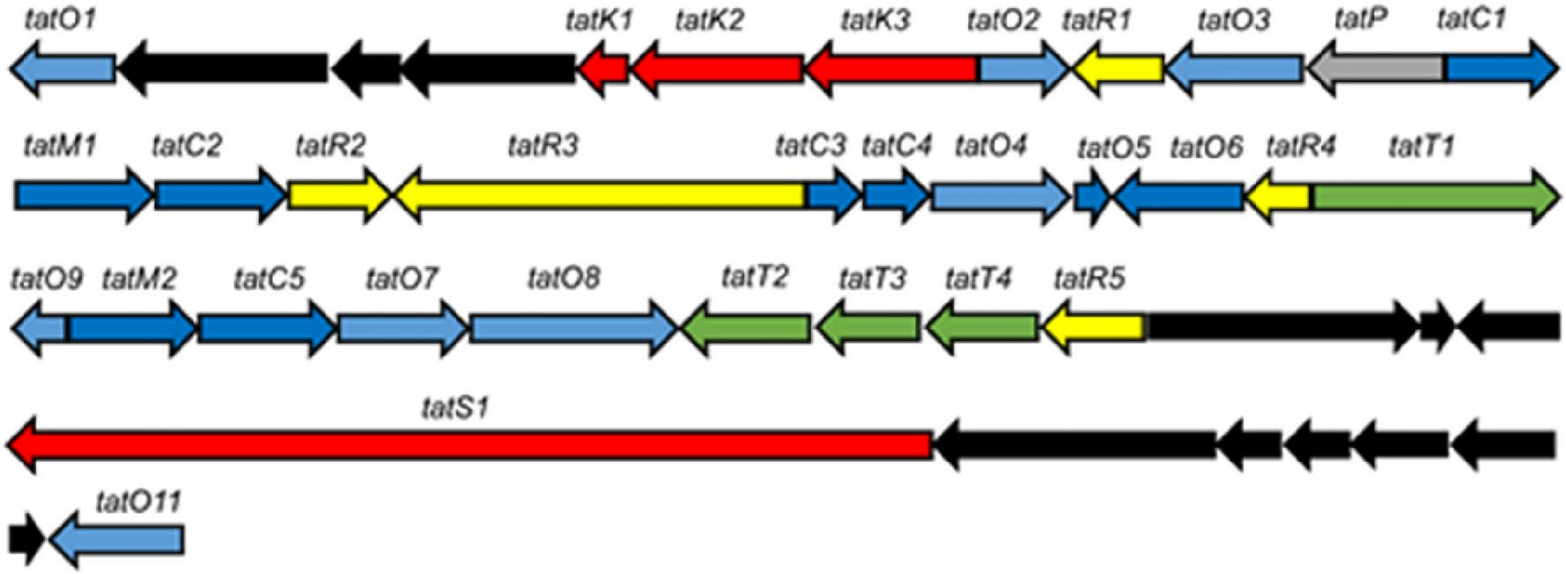
Organization of the tatiomicin BGC. Genes coding for polyketide biosynthesis (red; tatS = starter unit biosynthesis, tatK = chain biosynthesis), polyketide modification (blue; tatO = oxidoreductases, tatC = cyclases, tatM = methyltransferases), regulation (yellow; tatR), transport (green, tatT) and others (grey; tatP = phosphorylase, black; genes not assigned to the tatiomicin BGC based on homology to the rishirilide BGC and proposed biosynthetic pathway)).

Further inspection of the BGC from *Amycolatopsis* sp. DEM30355 revealed a highly altered gene synteny (see ESI), compared to the rishirilide BGC, along with the presence of several new genes: one postulated to be involved in PKS biosynthesis (*tatS1*), two encoding methyltransferases (*tatM1* and *tatM2*), two encoding cyclases (*tatC4* and *tatC5*) and one cytochrome p450 oxidoreductase (*tatO11*) (Fig. 2). Due to the significant variation in the genetic make-up of the BGC, we postulated that it may code for the production of an as yet undiscovered polyketide and as such we set about attempting to identify this molecule from the metabolome of *Amycolatopsis* sp. DEM30355.

Preliminary analysis of the fermentation supernatant of *Amycolatopsis* sp. DEM30355 by HPLC-HRMS showed the presence of a large number of secondary metabolites, in keeping with the predicted number of BGCs, including a compound with activity against Gram-positive bacteria (MW of 402 Da, *m*/*z* = 403 [M + H]^+^, *m*/*z* = 425 [M + Na]^+^, (–)-tatiomicin (**3**)). Fermentation of *Amycolatopsis* sp. DEM30355, removal of the biomass, extraction of the supernatant and bioactivity guided fractionation by multiple chromatography steps resulted in a fraction which retained antimicrobial activity and contained two closely related compounds. HRMS analysis suggested that these compounds were stereoisomers of each other, the major compound showing *m*/*z* = 425.1221 [M + Na]^+^ corresponding to a molecular formula of C_21_H_22_O_8_ for both molecules (see ESI).

Structural determination of the major component was initially performed by NMR, which provided the majority of molecular connectivity with the exception of the ordering of the three contiguous quaternary centres at the C-3, C-4 and C-4a positions. Structural assignment was completed via single-crystal X-ray diffraction (XRD) analysis, revealing a highly oxygenated anthracenone polyketide, structurally consistent with the BGC of interest, which we named (–)-tatiomicin (**3**) (Fig. 1)^29^.

NMR and HPLC experiments demonstrated that the minor compound was the *C*-2 epimer, capable of equilibrating with (–)-(**3**) under acidic conditions (see ESI).

Determination of the absolute stereochemistry of (–)-3 was undertaken in parallel via vibrational and electronic circular dichroism spectroscopies and additional single-crystal X-ray diffraction (SCXRD) experiments.

**Figure 3:**
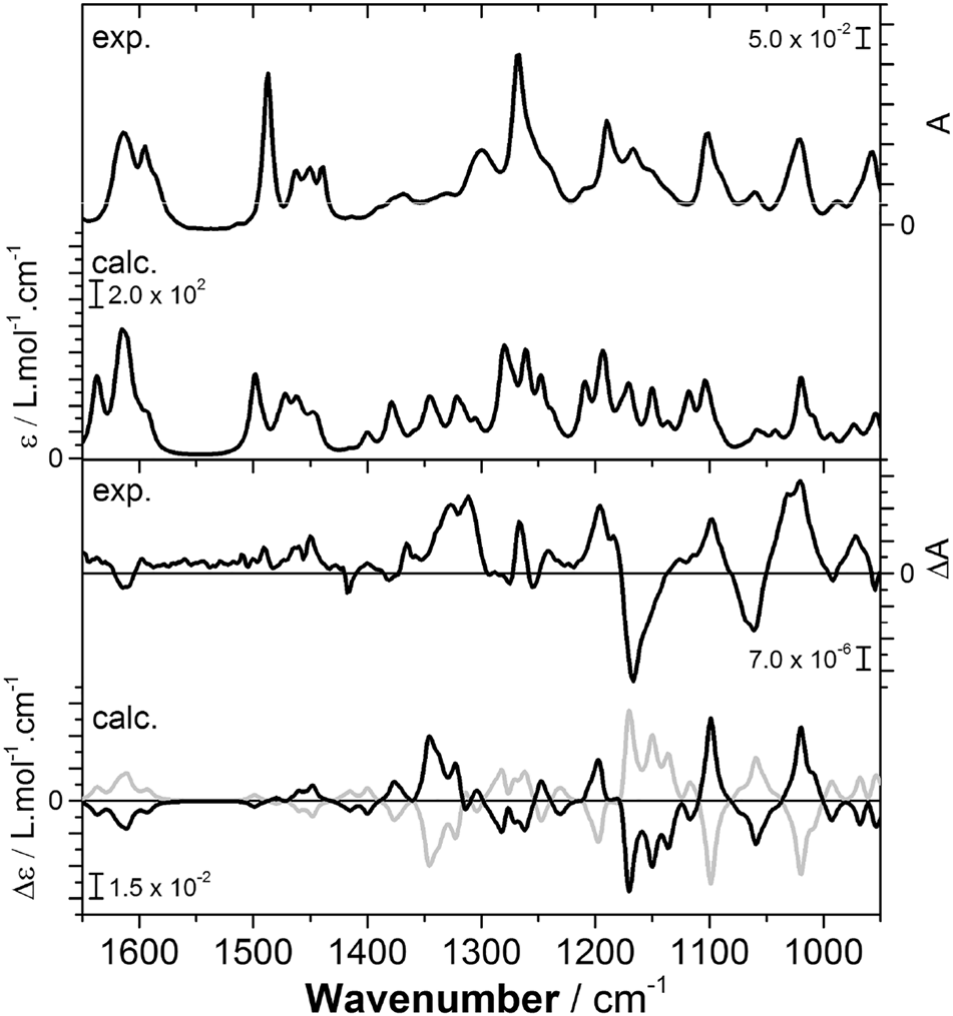
Experimental IR (top) and VCD spectra (bottom) of ( −)-tatiomicin 3 (CDCl_3_) with predicted spectra obtained at the B3LYP/PCM/6–311 +  + G(d,p) level of theory. VCD: Solid line = (2*R*,3*R*,4*S*,4a*S*,10*S*), dashed line = (2*S*,3*S*,4*R*,4a*R*,10*R*). Spectra have been frequency scaled Black line (σ = 0.987) to yield maximal similarity grey line between the computed and experimental VCD spectra.

Absolute configuration determination by vibrational circular dichroism (VCD) was based on a comparison of experimental and computationally predicted spectra, taking into account the presence of two epimers of (–)-3. Conformational analysis (see ESI), removal of redundant geometries and final optimization at the B3LYP/6–311 +  + G(d,p) level allowed Boltzmann-weighted VCD spectra for both epimers of (–)-3 to be constructed. The final predicted spectrum was obtained by applying a 3:1 ratio to account for the experimentally analysed mixture of epimers. Numerical analysis was used to establish agreement between experiment and theory, the neighbourhood similarity values (Σ^IR^ = 92.0, Σ^VCD^ = 71.2, ESI = − 57.8) suggesting an absolute stereochemical assignment of (2*S*,3*S*,4*R*,4a*R*,10*R*) (Fig. 3 and ESI)^30^. The assignment was supported through similar electronic circular dichroism (ECD) experiments; however, in this case correlation between experiment and prediction was weaker (see ESI).

**Figure 4:**
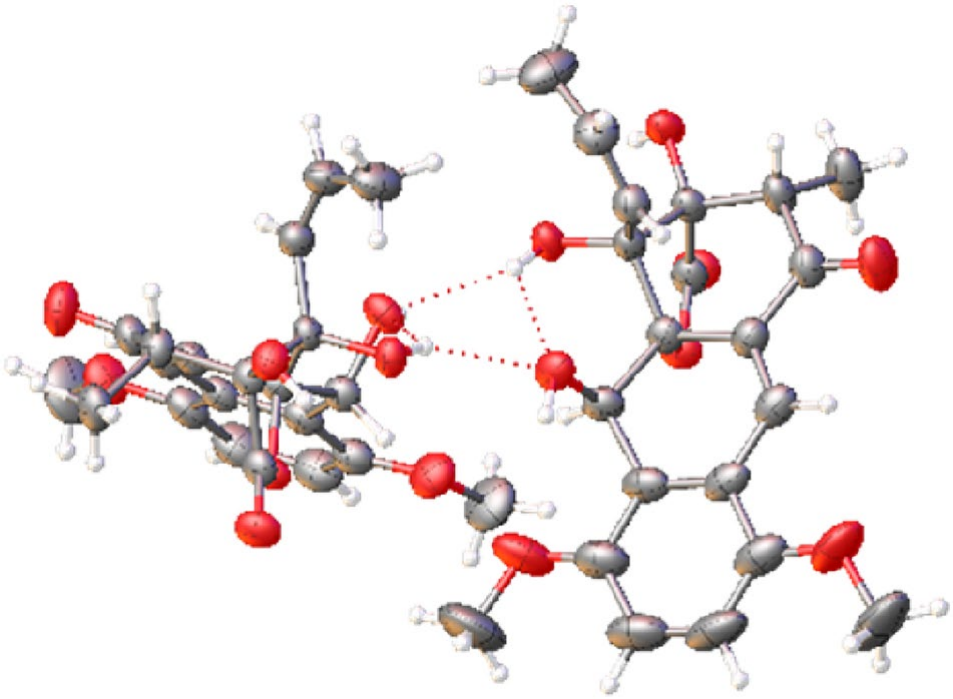
Displacement ellipsoid plot of the molecular structure of (–)-tatiomicin (**3**), absolute stereochemistry as shown determined by resonant scattering the dimer molecular structure (Flack parameter = 0.05(6)). Displacement ellipsoids shown at 50% probability level.

A suitable, albeit small, single-crystal of tatiomicin (**3**) was grown via slow evaporation from a benzene solution. Due to the crystal’s dimensions, diffraction data were collected at beam line I19 at the Diamond Light Source using synchrotron radiation at standard operating wavelength (λ = 0.6889 Å), providing a data set of sufficient quality to allow for structural confirmation. (–)-Tatiomicin (**3**) crystallized as an H-bonded dimer in the unit cell (Z’ = 2) along with a single molecule of solvent (benzene). To validate the absolute stereochemical assignment a further single-crystal X-ray diffraction experiment was undertaken at I19, employing non-typical, longer wavelength synchrotron radiation (λ = 1.4879 Å) to enhance resonant scattering contributions (also known inappropriately as anomalous dispersion). The absolute-structure (‘Flack’) parameter (0.05(6)) was insignificantly different from zero and with a small standard uncertainty, indicating the correct absolute configuration in the refined (2*S*,3*S*,4*R*,4a*R*,10*R*) structure (see ESI)^29^. Interestingly, following extensive stereochemical debate and several reported total syntheses, the absolute stereochemistry of the congeneric (+)-rishirilide B (**2**) was recently revised (2*S*,3*S*,4*S*), matching that of (–)-(**3**) over the three common stereocentres, suggesting a similar biosynthetic pathway for both sets of natural products (Fig. 4)^31–35^.

To verify that the BGC previously identified does indeed encode the biosynthetic pathway for tatiomicin (**3**), a high molecular-weight P1 artificial chromosome (PAC) library was obtained, consisting of 2,688 clones with an average insert size of 138 kb which contained resistant markers for kanamycin (for *E. coli*) and thiostreptone (for *S. coelicolor*). The PAC library was screened by PCR, using four primer pairs for the putative BGC. A single PAC clone was identified with the required PCR profile, which was then transferred into *E. coli* strain ET12567/pR9604 (dam^-^ dcm^-^), the plasmid was subsequently transferred into *S. coelicolor* M1152 via conjugation. Exconjugants containing the plasmid integrated on the chromosome were selected for resistance to thiostrepton. Ninety-six putatively identified exconjugants were arrayed into 24 well plates and screened for the production of tatiomicin (**3**) by TLC, with detection based on the characteristic fluorescence upon UV irradiation at 365 nm. Based on these screening parameters, *S. coelicolor* M1152::tat was identified as a producer of tatiomicin (**3**﻿) (see ESI).

Growth of *S. coelicolor* M1152::tat was examined on solid medium, the agar was extracted (EtOAc) and analysed by LCMS alongside similar fermentation extracts from both the parent strain M1152, *Amycolatopsis* sp. DEM30355 and a tatiomicin (**3**) standard. An LCMS peak corresponding to tatiomicin (**3**) was observed in the extract from *S. coelicolor* M1152::tat but was absent in that of the parent strain M1152 (Fig. 6).

**Figure 5:**
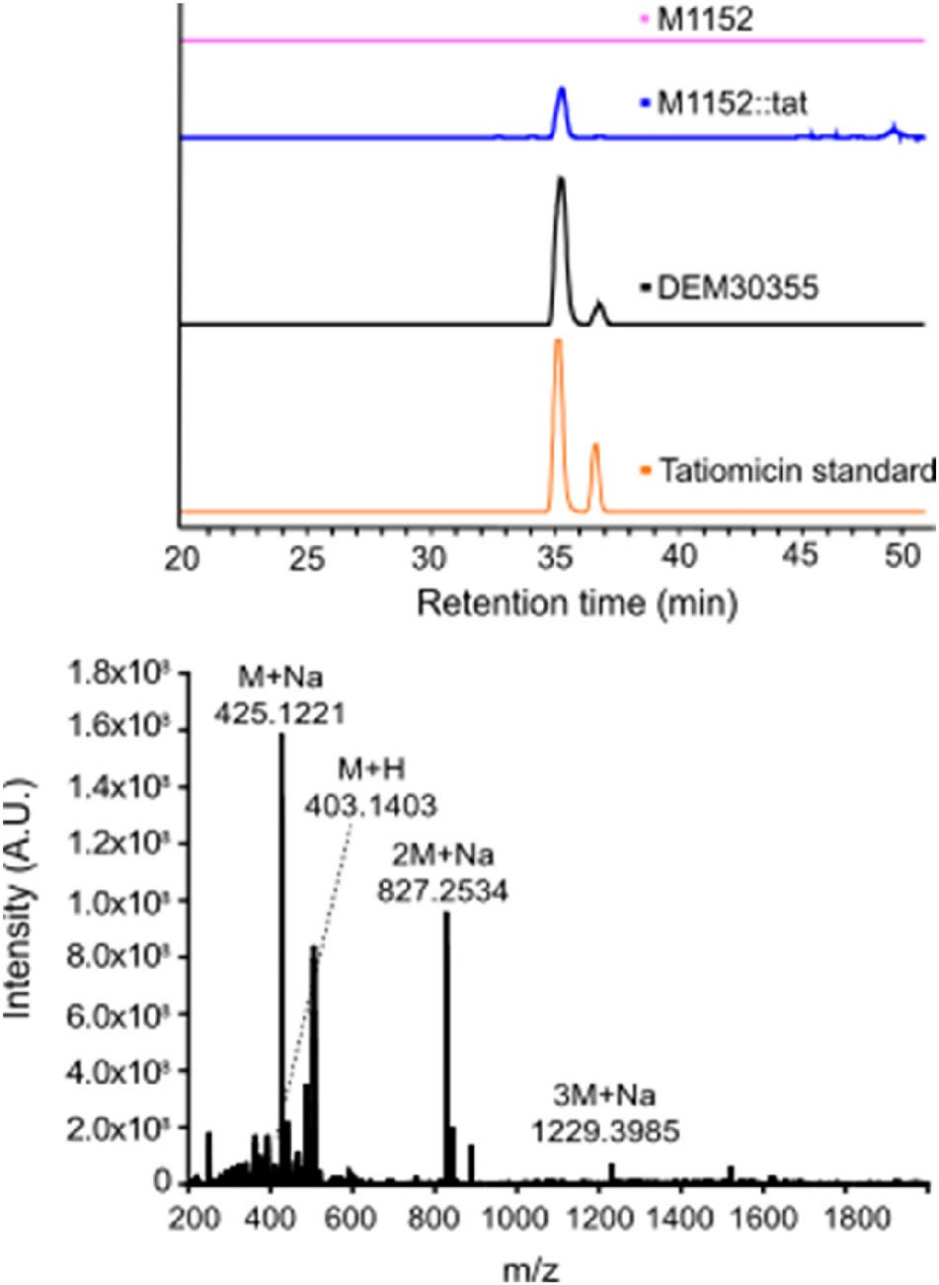
Detection of tatiomicin from the fermentation of heterologous host *S. coelicolor* M1152::tat. Top) Extracted ion chromatogram (EIC) based on *m/z* = 827.25. *S. coelicolor* M1152 (purple), *S. coelicolor* M1152::tat (blue), *Amycolatopsis* sp. DEM30355 (black) and tatiomicin standard (red). Bottom) MS spectrum of tatiomicin (**3**) purified from the heterologous host *S. coelicolor* M1152::tat.

Tatiomicin (**3**) was subsequently isolated from the fermentation of *S. coelicolor* M1152::tat in liquid medium (GYMG), as demonstrated by HRMS ([M + H]^+^ = 403.1403), with a production level in the heterologous host estimated at 0.57 mg/L, confirming the identity of the tatiomicin BGC (Fig. 5).

Based on a comparison between the tatiomicin and rishirilide BGCs^22–26^ we propose the following biosynthetic pathway operates for the assembly of tatiomicin (**3**) (See ESI). The modular type I polyketide synthase TatS1 is likely responsible for the biosynthesis of the polyketide starter unit, *cis*-crotonyl-ACP, which is then elongated via the attachment of eight malonyl-CoA by minimal PKS enzymes TatK1, TatK2, and TatK3. TatC1, TatC2, TatC3 and TatO10 show close homology to rishirilide cyclases RslC1, RslC2, and RslC3 and C9-ketoreductase RslO10, respectively. Thus, TatC1 and TatO10 likely act together to form the A ring of tatiomicin (**3**), whilst TatC2 and TatC3 catalyse the formation of the B and C rings. Tailoring of the polyketide core likely involves oxidation of the C ring by TatO4, and installation of the C ring epoxide by flavin mononucleotide (FMN)-dependent monooxygenase TatO1 together with a putative flavin reductase, TatO2. Opening of the epoxide is proposed to be mediated by NADPH:acceptor oxidoreductase TatO5, followed by the key Baeyer–Villiger oxidation/rearrangement controlled by TatO9 and finally reduction of the B ring ketone by ketoreductase TatO8.

**Figure 6:**
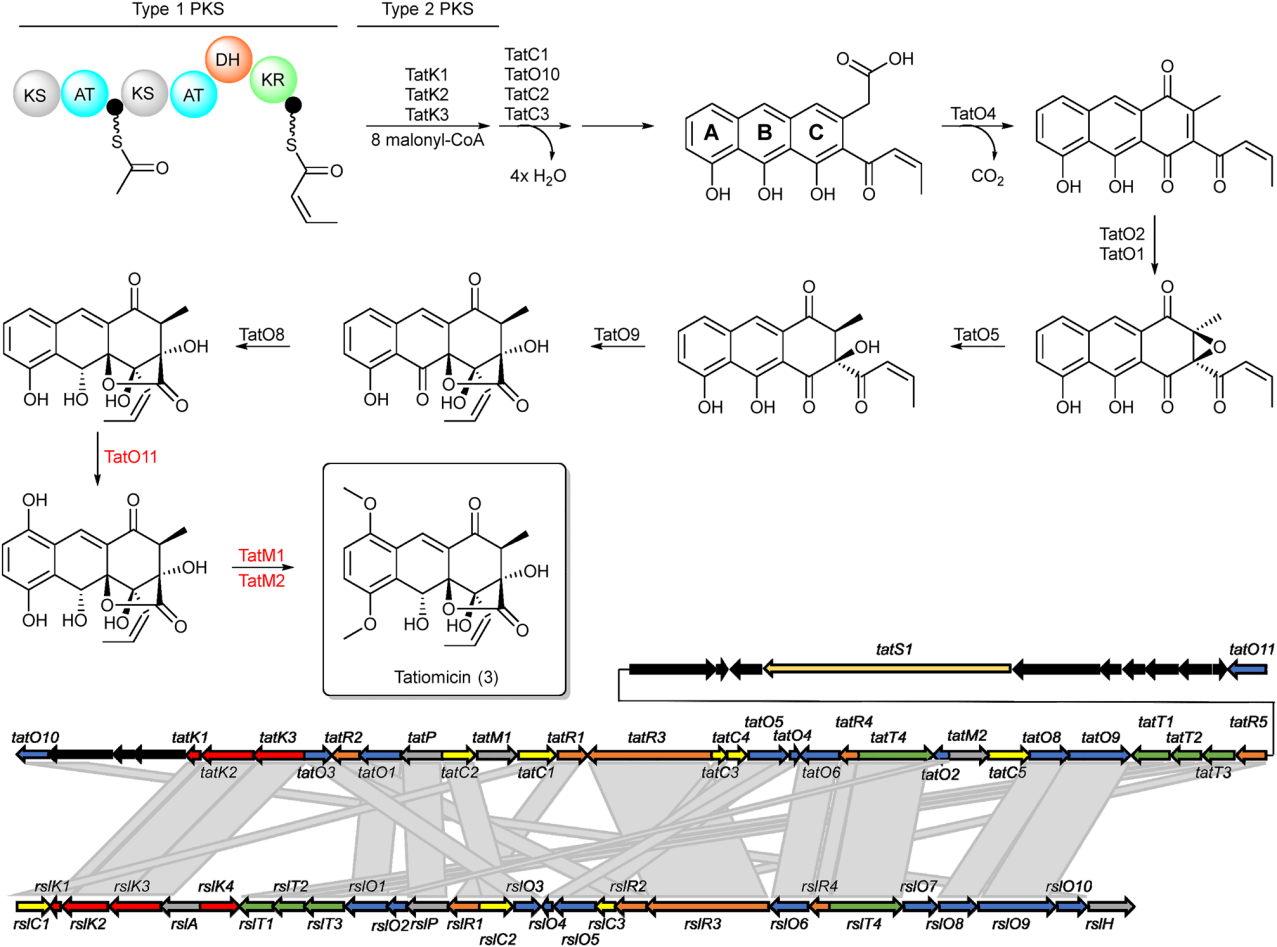
Top) Proposed pathway for the biosynthesis of (–)-tatiomicin (**3**) based on homology with the biosynthetic gene cluster for the rishirilides. Enzymes shown in red have no direct congener in the rishirilide BGC and their biosynthetic role is hypothesised, based on BLAST analysis. Bottom) comparison of the rishirilide and (-) tatiomicin gene cluter based on BLAST analysis. (red; tatS = starter unit biosynthesis, tatK or rslK = chain biosynthesis), polyketide modification (blue; tatO or rslO = oxidoreductases, tatC or rslO = cyclases), regulation (yellow; tatR or rslR), transport (green, tatT or rslT) and others (grey; tatP or rslP = phosphorylase; tatM = methyltransferases, black; genes not assigned to the tatiomicin BGC based on homology to the rishirilide BGC and proposed biosynthetic pathway)).

Three additional tailoring enzymes are present in the BGC of tatiomicin (**3**) for which no homologues are present in that of rishirilide, TatO11, TatM1 and TatM2. TatO11 is a cytochrome p450 oxidoreductase, likely responsible for oxidation of the A ring to the hydroquinone form, followed by double methylation by the two methyl transferases TatM1 and TatM2 to yield the completed molecule (Fig. 6).

The enzymes TatC4 and TatC5, which are not present in the rishirilide cluster, encode for a dehydrogenase and a monooxygenase and are located in the centre of the biosynthetic gene cluster. The tatiomicin BGC contains all orthologous genes responsible for the synthesis of rishirilide. The function of these additional genes is therefore not immediate obvious and might be a result of evolutionary divergence.

(–)-Tatiomicin (**3**) showed no detectable antimicrobial activity (MIC > 64 µg/mL) against ten Gram-negative bacteria and two eukaryotic microorganisms (*Candida* spp.) (see ESI). However, antibacterial activity was observed against a sub-set of Gram-positive bacteria (MIC = 4–8 µg/mL), namely *Staphylococcus* and *Streptococcus* species. Due to the interest in developing new antibiotics against drug-resistant *Staphylococcus* infections, we further evaluated (–)-3 against a panel of MRSA clinical isolates, including twenty-four EMRSA-15 and EMRSA-16 strains (the main causative agents of nosocomial epidemic MRSA bacteraemia in the UK, with resistance to penicillin, ciprofloxacin and erythromycin)^36^, and twelve MRSA strains isolated from Belgian, Finnish, French and German hospitals (see SI). In all cases antibiotic activity was maintained (MIC = 4–8 µg/mL), suggesting that (–)-3 does not operate via a mode-of-action previously encountered by these strains, prompting us towards further investigation.

Elucidation of the mode-of-action (MOA) for a new antibacterial agent is a significant experimental challenge. The characterization of resistance mutations can be informative, however all attempts to isolate *Bacillus subtilis* mutants resistant to (–)-tatiomicin (**3**) proved unsuccessful (see ESI). Also, no positive responses were seen with a panel of *B. subtilis* strains containing *lacZ* reporter genes used to indicate common antibacterial mechanisms of action, including: fatty acid synthesis (*fabHA*), DNA damage (*ɸ105* prophage induction), RNA polymerase (RNAP) inhibition (*helD*), cell wall damage (*ypuA*), gyrase inhibition (*gyrA*), and cell envelope stress (*liaI*)) (see ESI)^37–39^.

Due to the presence of an, albeit electron-rich, α,β-unsaturated carbonyl moiety, we postulated that the observed biological activity of (–)-tatiomicin (**3**) may involve the covalent modification of thiol-containing enzymes through a conjugate or Michael addition of the thiol to the α,β-unsaturated carbonyl. Thus, (–)-tatiomicin (**3**) was reacted with L-cysteine hydrochloride, L-cysteine methyl ester hydrochloride and a short thiol-containing peptide (LcrV (271–291)) as an enzyme proxy, under biologically relevant conditions. In all cases thiol adducts could be detected by LCMS, suggesting that (–)-tatiomicin (**3**) may have biologically relevant Michael acceptor activity (see ESI).

**Figure 7:**
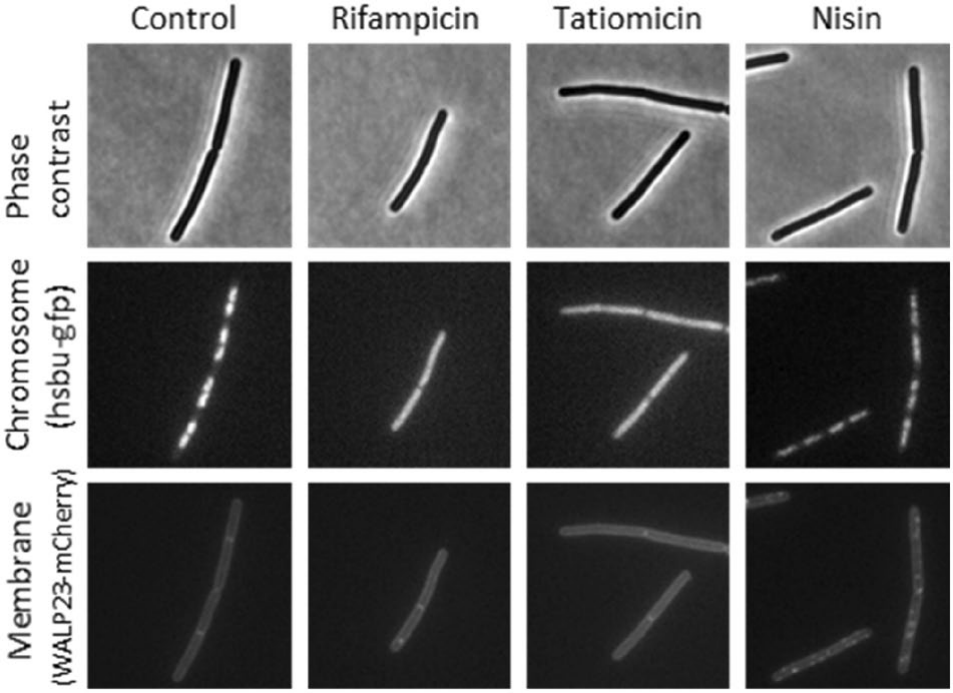
Single-cell analysis of chromosome and membrane integrity. Phase contrast (top panels) and fluorescence microscopy of *B. subtilis* cells treated with various antibiotics (indicated above). DNA was visualized with an HsbU-GFP fusion (middle panels) and the cytoplasmic membrane with a WALP23-mCherry fusion (bottom panels).

To gain further insight into a potential mode-of-action, we undertook a bacterial cytological profiling experiment in which antibacterial induced changes in the morphology of test bacteria are compared to those induced by known mode-of-action antibacterials^40,41^. *B. subtilis* 168CA-CRW419 expresses two fusion proteins, HbsU-GFP and WALP23-mCherry, allowing simultaneous visualization of both the chromosomal DNA and the bacterial cell membrane by fluorescence microscopy. The cytoplasmic membrane was unaffected unlike in the control compound nisin, which forms large pores in the membrane^42^. Interestingly, treatment with (–)-tatiomicin (**3**) induced chromosome decondensation in *B. subtilis* 168CA-CRW419, similar to the effects elicited by the RNAP inhibitor rifampicin (Fig. 7).

**Figure 8:**
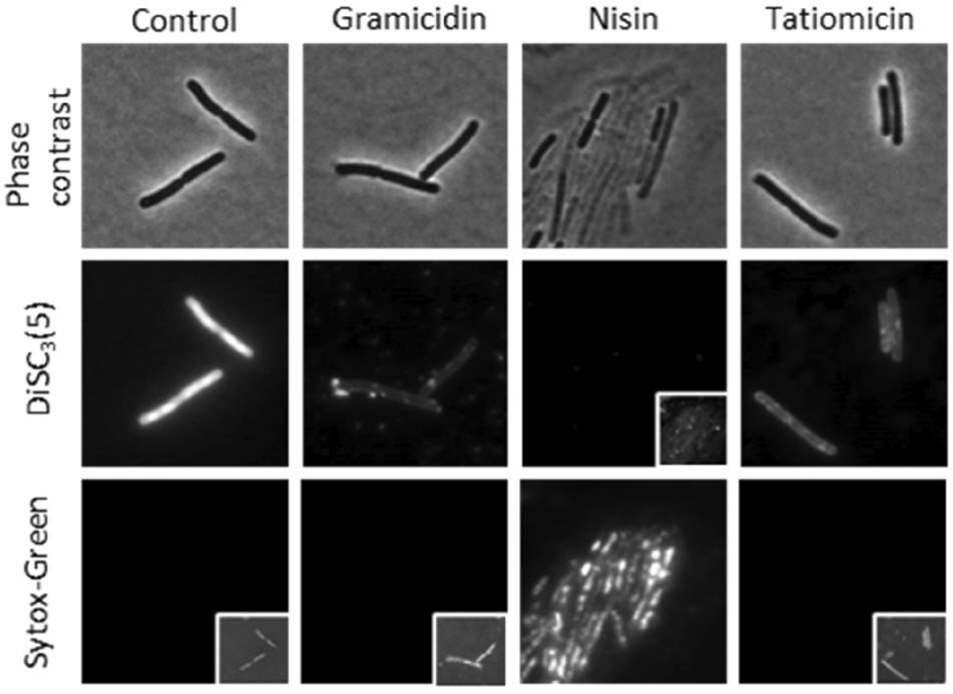
Single-cell measurement of membrane potential and permeability. Phase contrast (top panels) and fluorescence microscopy of *B. subtilis* cells stained with the voltage-sensitive dye DiSC3(5) (middle panels) and the membrane permeability indicator Sytox Green (bottom panels) in the presence and absence of 32 μg/mL of tatiomicin. As positive controls, the cells were treated with 5 μg/mL of gramicidin (membrane depolarisation without pore formation) and 10 μM nisin (membrane depolarisation through pore formation). Cellular DiSC3(5) and Sytox Green fluorescence values were quantified for cells treated with tatiomicin (32 μg/mL), gramicidin (5 μg/mL), and nisin (10 μM) (see SI).

The combination of the negative result observed with the helD reporter strain, cell lysis after prolonged incubation with the compound and the inability to create resistant mutants suggest that direct RNAP inhibition is unlikely. We therefore attempted to examine the integrity of the cytoplasmic membrane using the voltage sensitive dye DiSC3(5). This dye accumulates in well-energised cells in the cytoplasmic membrane^15,43^ but is released upon depolarisation of the membrane, and this release can be measured by fluorescence microscopy. DiSC3(5) is used in parallel with Sytox Green, a membrane-impermeable DNA stain used as a reporter for pore formation^44^. Upon addition of nisin, which forms large pores in the *B. subtilis* membrane^42^, both a loss of DiSC3(5) and uptake of Sytox Green was observed. In contrast gramicidin, which forms small cation-specific channels^45^, showed loss of DiSC3(5) without Sytox Green staining. Treatment with (–)-tatiomicin (**3**) showed a similar effect to that of gramicidin, i.e. loss of DiSC3(5) without Sytox Green staining. Hence tatiomicin probably acts to dissipate the membrane potential without the formation of large pores (Fig. 8).

**Figure 9:**
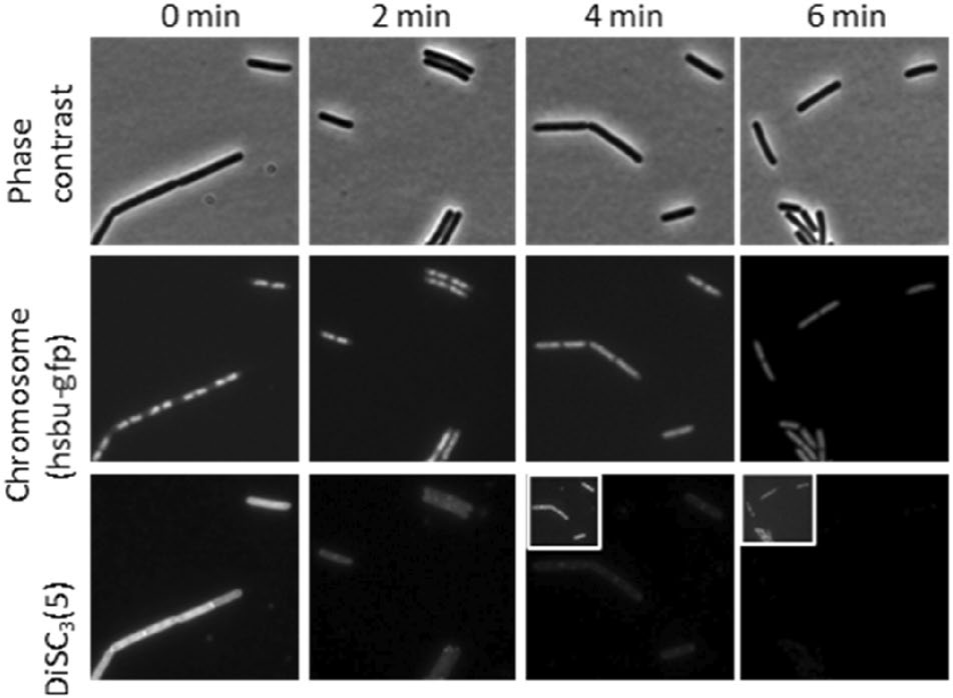
Single-cell measurement of chromosome decondensation and membrane potential in a time course experiment in the presence of tatiomicin (32 μg/mL). Phase contrast (top panels), fluorescence microscopy of *B. subtilis* HsbUGFP (chromosome marker) (middle panel) and stained with the voltage sensitive dye DiSC3(5) bottom panel. Cellular DiSC3(5) fluorescence values where quantified over time. The bar chart depicts the fluorescent intensity values of individual cells (> 30) (see SI).

In an attempt to ascertain whether the observed loss of membrane potential is a downstream effect or occurs at the same time as chromosome depolarisation we performed a time-course experiment using DiSC3(5) in combination with a HsbU-GFP fusion to assess chromosome decondensation with images taken every two minutes. This showed that the loss of membrane potential occurred simultaneously with the chromosome decondensation, between 2 to 4 min, suggesting that they are closely linked events (Fig. 9).

## Conclusions

We have identified (–)-tatiomicin (**3**) from *Amycolatopsis sp.* DEM30355, isolated from an hyper-arid Atacama desert soil, which shows antibiotic activity against a range of Gram-positive bacteria including MRSA clinical isolates. Comprehensive structural elucidation included absolute stereochemical assignment by complementary VCD and single crystal X-ray diffraction analysis, using long wavelength synchrotron radiation. The biosynthetic gene cluster of (–)-tatiomicin (**3**) was identified and validated through expression in a heterologous host, allowing us to propose a biosynthetic pathway. Single-cell microscopy studies suggest a pleiotropic mode-of-action involving membrane depolarisation and chromosomal decondensation, potentially involving the covalent modification of a target protein(s), although a definitive mode-of-action remains elusive at this time. Thus, we have demonstrated that modern bioprospecting techniques when applied to actinobacteria from extreme environments can still result in the discovery of new natural products with important bioactivity.

## Methods

### Growth conditions of *Amycolatopsis sp.* DEM30355

Fermentation was carried out at the Centre for Process Innovation, UK. DEM30355 was transferred from GYMG plate (yeast extract 0.4%, malt extract 1%, glucose 0.4%, glycerol 1% and agar 1%) into two conical flasks filled with 10 mL of GYMG media (yeast extract 0.4%, malt extract 1%, glucose 0.4% and glycerol 1%). After 2 days of growth, cells were transferred into a 200 mL wv (working volume) flask. After incubation overnight, the two flasks were combined and an 8 L wv bioreactor was inoculated. An overnight incubation of a 50 L seed bioreactor was inoculated with 2.5 L of culture broth. Incubation for 15 h resulted in 40 L of exponentially growing cells at an OD450nm of 5.4. The 500 L wv bioreactor was inoculated with 25 L of this culture broth. The temperature was controlled at 30 °C. The dissolved oxygen was controlled at 50% via the agitation speed (starting speed 400 rpm). Compressed air was applied to the vessel at 1 vvm. The pH was adjusted to pH 7 prior to autoclaving.

### Isolation of tatiomicin

Biomass was removed via centrifugation (disc stack centrifuge, Satorious) and filtration Satorious depth filters). Amberlite XAD-16 absorption resin (10 kg) was applied to the supernatant. After overnight incubation under agitation, the beads were filtered off and washed with deionised water. Elution using methanol (40 L) was carried out. The solvent was evaporated to an aqueous extract. The pH of the aqueous extract was adjusted to 4 using sulphuric acid and twice extracted with ethyl acetate. The organic layer was evaporated to dryness resulting in 231.11 g of crude extract.

An aliquot (~ 4 g) of ethyl acetate extract, obtained from a 500 L fermentation of DEM30355, was dissolved in 20 mL of methanol (MeOH), absorbed onto 10 mL of chromatography grade silica and dried. This was loaded onto a 100 g silica SNAP Biotage cartridge and eluted with ethyl acetate (528 mL; 4 CV) at a flow rate of 50 mL/min and a fraction size of 50 mL. Fractions containing tatiomicin (Rf: 0.67, ethyl acetate, TLC) were combined and the organic solvent was evaporated under reduced pressure. The resulting black oil was dissolved in 5 mL of methanol and loaded onto an LH20 size exclusion column (GE Healthcare XK 26/100; 2.6 cm diameter, 90 cm bed height). The column was eluted with methanol at a flow rate of 1 mL/min. The first 80 mL of eluent (1 CV) was discarded, after which 10 mL fractions were collected to give 80 fractions in total. The fractions were analysed by TLC and those containing tatiomicin were combined and the solvent evaporated under reduced pressure. The resulting dark yellow oil was dissolved in 5 mL of methanol, absorbed onto 5 mL of chromatography grade silica, dried and loaded onto a 50 g silica SNAP Biotage cartridge. The column was eluted with diethyl ether at a flow rate of 50 mL/min, with 50 mL fractions collected. The fractions were analysed by TLC and those containing tatiomicin, as a mixture of two epimers, were combined, and the solvent evaporated under reduced pressure to give 171.5 mg of yellow powder.

### Chemical characterisation of tatiomicin and tatiomicin*

#### Tatiomicin

[α]_D_ -78 (c 0.3, CH_2_Cl_2_); ^1^H NMR (700 MHz, CD_2_Cl_2_) δ_H_ 8.12 (1H, s), 7.05 (1H, d, *J* = 9.1 Hz), 6.92 (1H, d, *J* = 9.2 Hz), 5.77 (1H, dq, *J* = 12.0, 7.2 Hz), 5.49 (1H, s), 5.47 (1H, dq, *J* = 12.0, 2.0 Hz), 3.85 (3H, s) 3.84 (3H, s), 2.78 (1H, q, *J* = 7.6 Hz), 1.94 (3H, dd, *J* = 7.2, 2.0 Hz), 1.20 (3H, d, *J* = 7.6 Hz); ^13^C NMR (175 MHz, CD_2_Cl_2_) δ_C_ 196.3, 174.9, 152.9, 151.2, 133.2, 131.9, 129.0, 124.7, 123.3, 118.9, 115.9, 112.8, 83.5, 82.7, 81.7, 66.5, 56.5, 56.4, 49.8, 15.6, 11.3; HRESIMS *m*/*z* found 425.1207, C_21_H_22_O_8_Na requires 425.1207.

#### Tatiomicin*

^1^H NMR (700 MHz, CD_2_Cl_2_) δ_H_ 8.14 (1H, s), 7.07 (1H, d, *J* = 9.1 Hz), 6.95 (1H, d, *J* = 9.1 Hz), 5.76 (1H, dq, *J* = 11.9, 7.3 Hz), 5.52 (1H, s), 5.54–5.50 (1H, m), 3.88 (3H, s) 3.87 (3H, s), 2.81 (1H, q, *J* = 7.4 Hz), 1.98 (3H, *J* = 7.3, 1.7 Hz), 1.41 (3H, d, *J* = 7.4 Hz); ^13^C NMR (175 MHz, CD_2_Cl_2_) δ_C_ 196.3, 177.7, 153.1, 151.5, 133.9, 132.0, 129.4, 125.8, 123.5, 119.2, 116.1, 113.1, 83.7, 81.9, 80.0, 66.8, 56.8, 56.7, 49.7, 15.9, 11.3.

### DNA extraction, sequencing, genome assembly and bioinformatic analysis

Exponentially growing cells (20 mL) were harvested (3000 rpm; 15 min), lysed and the DNA was purified via a phenol–chloroform extraction. The purity of extracted DNA was assessed via NanoDrop and visualisation on an agarose gel. Sequencing was performed using the next-generation sequencing platforms of Illumina HiSeq 2000 technology (Illumina) and PacBio (Pacific Biosciences). The data received from PacBio sequencing was assembled into 13 contigs using the open source PacBio software SMRTportal and subsequently corrected with the illumina reads using CLC Genomic Workbench 7.0.4 with a trial license. The gene sequence of the tatiomicin gene cluster was analysed using AntiSMASH 6.0.1 The cluster was visualized in artemis. The rishirilide gene cluster was retrieved from the MIBiG database (MGC0001179) and using the Blastp algorithm manually aligned to the tatiomicin gene cluster to find homologous genes (Table S11). The nucleotide sequence of the tatiomicin cluster was deposited in the NCBI database with the accession number (ON685203).

### Creation of bacterial artificial chromosome (PAC) library

A genomic library of *Amycolatopsis* strain DEM30355 DNA was constructed in pESAC13 (by BioS&T). The supplied genomic library was screened for the tatiomicin gene cluster using PCR with multiple primer pairs (Table S12). PCR amplifications was performed using Herculase II Fusion according to manufacturer’s protocols. This resulted in one PAC clone (61H7) being identified which was transferred together with pR9604 into E. coli ET1256. We subsequently conjugated the plasmid pEASAC13_61H7 into S. coelicolor M1152.

### Analysis of exconjugants

The received exconjugants were arrayed into five 24 well plates filled with 4 mL of GYMG media (0.4% yeast extract, 1% malt extract, 0.4% glucose and 1% glycerol) and incubated shaking at 300 rpm for 5 days at 30 °C. The supernatant was subsequently freeze dried and resuspended in 100 µL DMSO. This was subjected to TLC analysis in ethyl acetate (tatiomicin *R*_*f*_ 0.67, ethyl acetate). Six strains showed putative tatiomicin production and were subjected to expression analysis at a 500 mL scale in GYMG media in baffled flask for 5 days at 30 °C. One exconjugant showed via TLC clear tatiomicin production. The supernatant was delipified using petroleum ether before extraction of tatiomicin using diethyl ether at equal volume. The tatiomicin extract was then subjected to HPLC and MS analysis. Alternatively, the strain was grown on GYMG agar (7 plates in total). The plates were homogenised, freeze thawed and extracted with 500 mL ethyl acetate and subjected to LC–MS analysis.

### MIC determination against a panel of bacterial isolates

Agar titration was performed in accordance with British Society for Antimicrobial Chemotherapy guidelines^46^, against a collection of 54 bacterial and 2 yeast isolates. The collection included 16 isolates acquired from the National Collection of Type Cultures (NCTC, Colindale, UK), 3 isolates acquired from the American Type Culture Collection (ATCC, Manassas, USA), 1 isolate from the National Collection of Pathogenic Fungi (NCPF, Colindale, UK) and 36 MRSA strains frequently encountered in Europe. Tatiomicin/tatiomicin* (3:1) was prepared at a stock concentration of 10 mg/mL in 100% DMSO, which was tested in IsoSensitest agar at a concentration range of 0.031 to 64 μg/mL, and inoculated using a multipoint inoculator to deliver a final concentration of 10^4^ CFU/1 µL spot of each isolate. An inhibitor-free control plate was also included. Incubation occurred at 37 ± 0.5 °C for 18 h. The MIC was defined as the lowest concentration of tatiomicin/tatiomicin* inhibiting visual growth after overnight incubation.

### Assessment of Michael acceptor ability of tatiomicin

#### Stock solution preparation

A stock solution of tatiomicin (10 mg, 0.025 mmol) was prepared in DMSO (10 mL) to give a 2.50 mM solution. A stock solution of L-cysteine hydrochloride (1.57 mg, 0.0100 mmol) was prepared in deionised water (1 mL) to give 0.01 M solution. A stock solution of L-cysteine methyl ester hydrochloride (1.72 mg, 0.0100 mmol) was prepared in deionised water (1 mL) to give 0.01 M solution.

#### Experiments with L-cysteine and L-cysteine methyl ester

To a 5 mL pear shaped RBF, was added water (725 µL), 0.01 M solution of L-cysteine hydrochloride or L-cysteine methyl ester hydrochloride in water (75 µL, 0.75 µmol) and a 2.50 mM solution of tatiomicin in DMSO (200 µL, 0.50 µmol). The reactions were stirred for 180 min at room temperature. After 30, 60, 90, 120, 150 and 180 min, a 50 µL aliquot of each reaction mixture was diluted into 250 µL of water, and then analysed by LCMS to identify possible Michael addition products.

#### Stock solution preparation

A stock solution of tatiomicin (10 mg, 0.025 mmol) was prepared in DMSO (1 mL) to give a 25 mM solution. A 4.5 mM solution of LcrV (272–291) was prepared in 200 mM Tris buffer (pH 8) in 20 mM DTT (as a stabilizer).

#### Experiments with short peptide LcrV (272–291)

To a 0.2 mL Eppendorf tube, was added water (2.6 µL), 4.5 mM solution of LcrV (271–291) in 20 mM DTT (4.4 µL, 0.020 µmol), 200 mM solution of Tris buffer 200 mM (pH 8) (5 µL, 1.0 µmol) and 25 mM solution of tatiomicin in DMSO (8 µL, 0.20 µmol). The resulting mixture was mixed for 5 s (vortex) at room temperature. After 0, 15, 30, 45 and 60 min, a 2 µL aliquot of the reaction mixture was taken, diluted with 10 µL water and then analysed by LCMS to examine the presence of Michael addition products.

### Single cell microscopy

*B. subtilis* 168CA was grown to an OD600 of 0.1 in LB medium at 37 °C while shaking. Tatiomicin/tatiomicin* was imaged at 2 × the inhibitory concentration at an OD600 0.1 (32 µg/mL). The control antibiotics rifampicin at 3 µg/mL, nisin at 33 μg/mL and gramicidin at 38 µg/mL (mix of gramicidin A–D). We assayed the effect of the compounds after 8 min of incubation with the compounds by transferring the cells onto a microscope slides covered with 1.2% agarose in water. For DiSC3(5) and Sytox Green staining a final concentration of 2 μM and 50 nM was used retrospectively. Cells were incubated for 5 min at 37 degrees before adding the compounds. Microscopy was carried out with Nikon Eclipse Ti (Nikon Plan Apo 1.40 Oil Ph3 objective) and the images acquired with Prime 4.2 sCMOS camera (Photometrics) and Metamorph 7 (Molecular Devices).

## Supplementary Information

Supplementary Information.

## Data Availability

All the data generated or analysed during this study are available as Supplementary Information files. The tatiomicin gene cluster has been deposited in NCBI GenBank under accession number of ON685203.
