# Supplementary material for: Discovery, isolation, heterologous expression and mode-of-action studies of the antibiotic polyketide tatiomicin from Amycolatopsis sp. DEM30355
Source: Sci Rep. 2022 Sep 16;12:15579. doi: 10.1038/s41598-022-18726-3 (PMC9481585; doi:10.1038/s41598-022-18726-3)
Supplement: Supplementary file 1 — Supplementary Information. [file 41598_2022_18726_MOESM1_ESM.pdf]

Table of Contents

Identification and Classification of DEM30355 **S1**

Phylogenetic Analysis of DEM30355 S1

Neighbour-joining tree for DEM30355 S2

Photographic Images of DEM30355 S3

Scanning Electron Microscopy of DEM30355 S4

Fermentation of DEM30355 **S5**

Structural Analysis of Tatiomicin/Tatiomicin* **S6**

HPLC Separation of the Epimers of Tatiomicin S6

Evaluation of Epimer Stability by HPLC S6

UV Absorption Spectra of Tatiomicin and Tatiomicin* S7

LCMS of Tatiomicin and Tatiomicin* S8

Chemical Characterization of Tatiomicin/Tatiomicin* **S9**

Chemical Characterization of Tatiomicin and Tatiomicin* including optical rotation S9

Comparison of ^{1}H NMR data of Tatiomicin vs Tatiomicin* S10

NMR data for Tatiomicin/Tatiomicin* S11

NMR data for Tatiomicin S14

NMR data for Tatiomicin* S17

X-Ray Crystallographic Studies of Tatiomicin **S20**

Crystallography Data Tables for Tatiomicin S20

Computational Methods **S31**

Conformers of Tatiomicin S31

Conformers of Tatiomicin* S32

Experimental and Calculated VCD and ECD Spectra S33

Cartesian coordinates of calculated conformers S34

Genome sequencing and analysis of biosynthetic pathway **S49**

Bioinformatic analysis S49

Proposed biosynthesis of tatiomicin S55

Heterologous expression of tatiomicin **S57**

Analysis of Exconjugants S57

Bioactivity Studies **S59**

MIC determination against a panel of bacterial isolates S59

Mode of Action Studies **S61**

Reporter strain panel S61

Assessment of Michael acceptor ability of tatiomicin S62

Single cell microscopy S65
Creation of tatiomicin/tatiomicin* resistant mutants S66
Microorganisms Used **S67**
References **S68**

Identification and Classification of DEM30355

Phylogenetic analysis of DEM30355

The total genomic DNA was extracted using Gene Elute™ Bacterial Genomic Kits (Sigma), following the manufacturer's protocol for the analysis of Gram-positive bacteria**.** An amplification of the 16S gene was performed using the primers 27F and 1525R.

Table S1 PCR conditions for amplification of 16S gene

	Time in min	Temperature in °C	
Initial denaturation	5	95	
Denaturation	1	94	30 Cycles
Annealing	1	58	
Extension	1	72	
Final Extension	10	72	

Primers Employed:

27f 5'-AGAGTTTGATCMTGGCTCAG

1525r 5'-AAGGAGGTGWTCCARCC

The PCR product was purified using GeneJet PCR purification Kit (Thermo Scientific) and the product was subsequently sequenced at SolGent Co., Ltd. The resulting sequences were trimmed and assembled in a DNA Baser and uploaded for identification to the online server EZTaxon. The 16S sequences of related *Amycolatopsis* and the *Prauserella rugosa* (GenBank accession no AB297964.1) was downloaded from EZTaxon and imported into the Mega X software package to perform neighbour-joining maximum likelihood and maximum parsimony tree-making algorithm based on 1000 bootstrap iterations.

Neighbour-joining tree for DEM30355

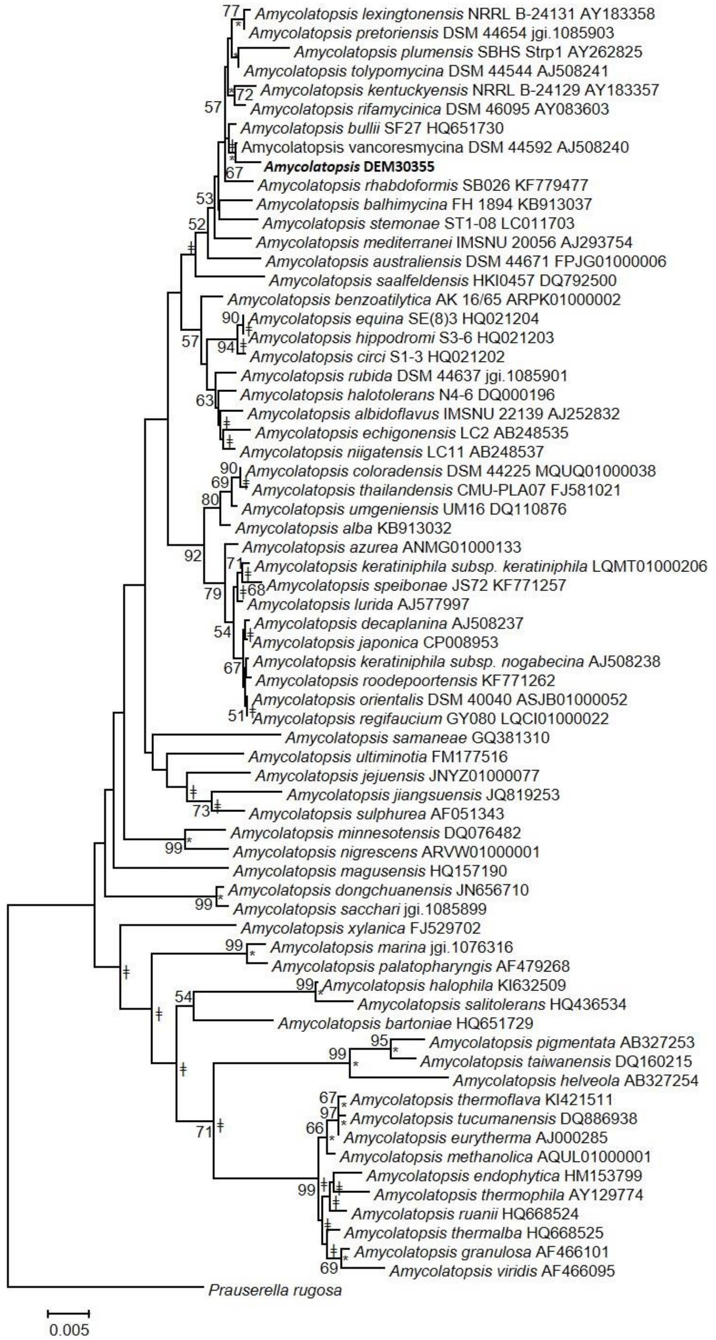

Figure S1 Neighbour-joining tree based on nearly complete 16S rRNA sequences showing relationships between isolate DEM 30355 and the type strains of *Amycolatopsis* species. Stars (*)indicate branches that were also recovered using the maximum-likelihood and maximum-parsimony tree-making algorithm; palatal click (ǂ) indicate branches that were also recovered with the maximum-likelihood tree-making algorithm. Numbers on the nodes are percentage bootstrap values based on a neighbour-joining analysis of 1000 resampled datasets. Only values >50 % are shown. *Prauserella rugosa* (GenBank accession no AB297964.1) was used as an outgroup.

Photographic Images of DEM30355

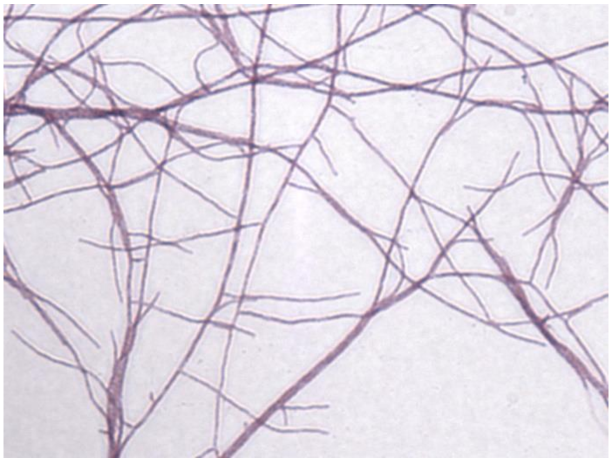

Figure S2 Microscope image of *Amycolatopsis* sp. DEM30355 with Gram-stain

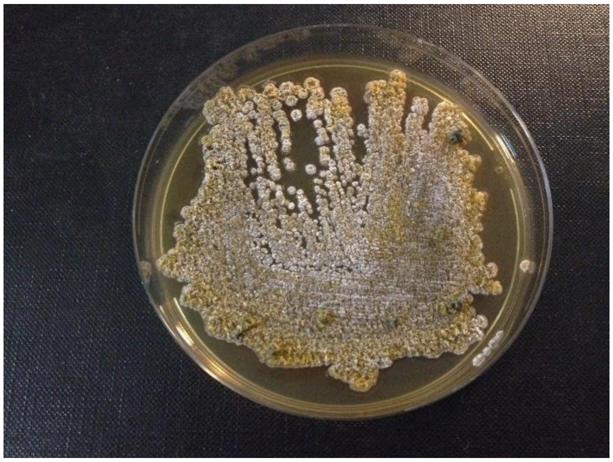

Figure S3 *Amycolatopsis* sp. DEM30355 grown on GYMG agar

Scanning Electron Microscopy of DEM30355

Amycolatopsis isolate DEM30355 was fixed overnight in 2% glutaraldehyde in Sorenson's Phosphate buffer (0.1 M sodium phosphate buffer, pH 7.2) then rinsed in several changes of Sorenson's buffer. The cells were dehydrated using 25%, 50% and 75% ethanol for 30min each, followed by 100% ethanol x2 for 1h, and carbon dioxide in a Baltec Critical Point Dryer. The specimen was mounted on an aluminium stub with Achesons Silver Dag and dried overnight. The sample was coated with gold, 5-10 nm using a Polaron SEM Coating Unit. The specimen was examined using a TESCAN VEGA LMU Scanning Electron Microscope housed within the EM Research Services (Newcastle University).

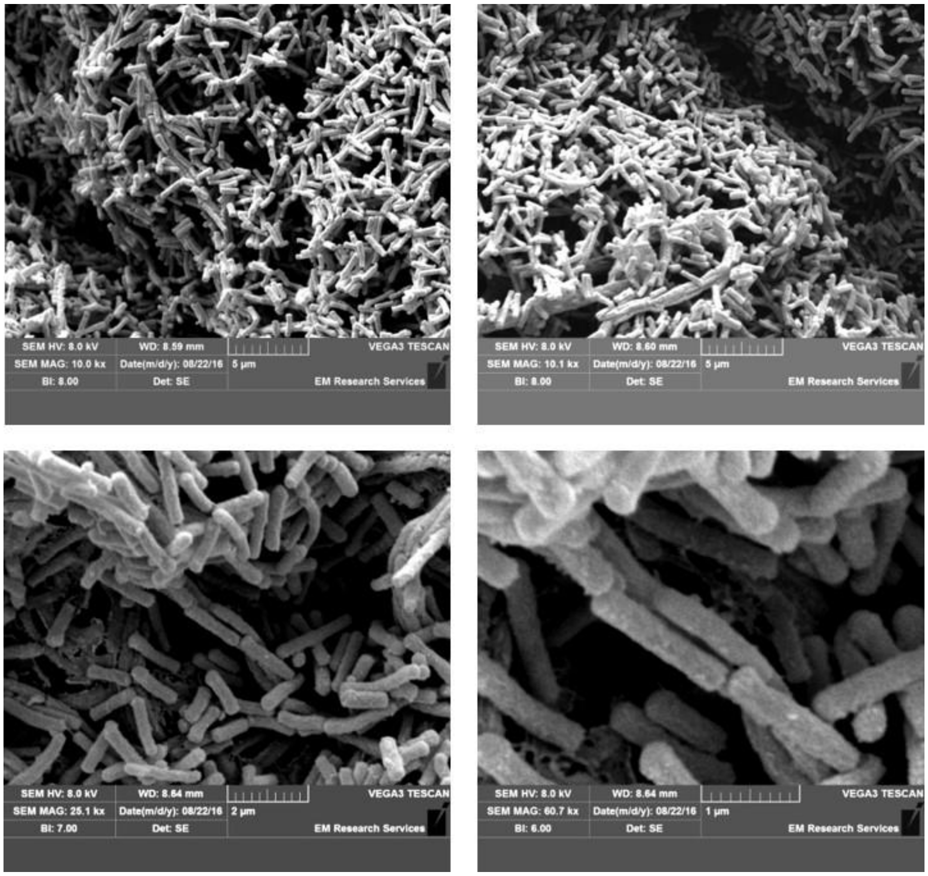

Figure S4 SEM images of DEM30355

Fermentation of DEM30355

The optical density was measured at 450 nm. The dry cell weight was determined using 15 mL falcon tubes in triplicates. Determination of glucose was performed using the SD Codefree blood glucose monitoring system with disposable strips. Alkaline phosphatase and phosphate was determined using published methods.[1]

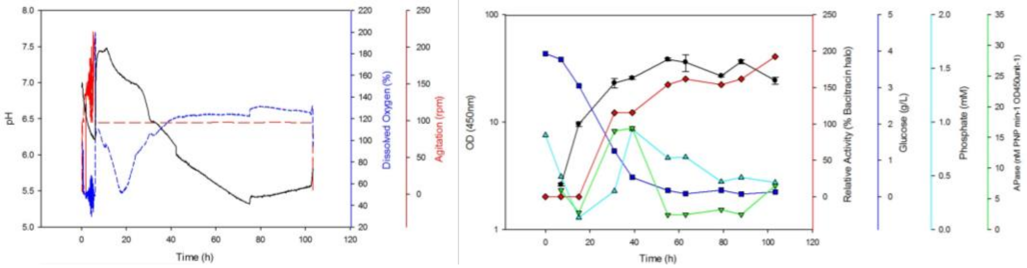

Figure S5 DEM30355 grown in 500 L bioreactor in GYMG over 103.5h: (left) Online measurements; (—), pH was controlled at pH 7; (--), Dissolved Oxygen; (– –), Agitation; (right) Offline measurements (★) Dry cell weight; (●), Optical density; (♦), Relative activity; (■), Glucose concentration; (▲), Phosphate concentration; (▼), Alkaline Phosphatase activity.

Structural Analysis of Tatiomicin

HPLC Separation of the Epimers of Tatiomicin

A sample of tatiomicin, as a mixture of epimers, was further purified via HPLC to provide material for NMR analysis (using an Agilent 1260 with an integrated fraction collector on a semi-preparative C18 column (250 x 10 mm, 5 µm)). A linear gradient from 30% over 60 min (water (0.1 % formic acid): acetonitrile (0.1 % formic acid)) at a flow rate of 2.5 mL/min was performed with monitoring by UV/vis absorption at 325 nm.

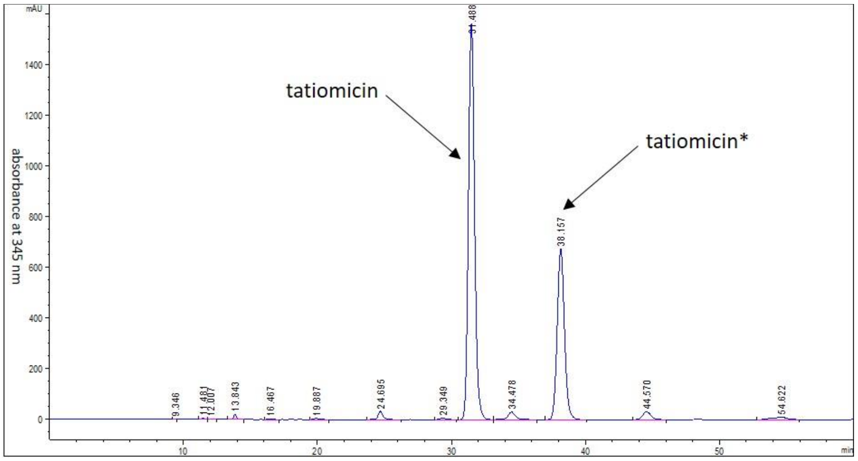

Figure S6 HPLC separation of the epimers of tatiomicin, UV-vis absorption recorded at 325 nm.

Evaluation of Epimer Stability by HPLC

In order to evaluate the stability of the minor epimer of tatiomicin (tatiomicin*), immediately following HPLC separation fractions containing tatiomicin* were collected and HPLC analysis was carried out at 2 hour intervals. After 8 h, nearly half of minor epimer of tatiomicin had epimerised to the corresponding major epimer.

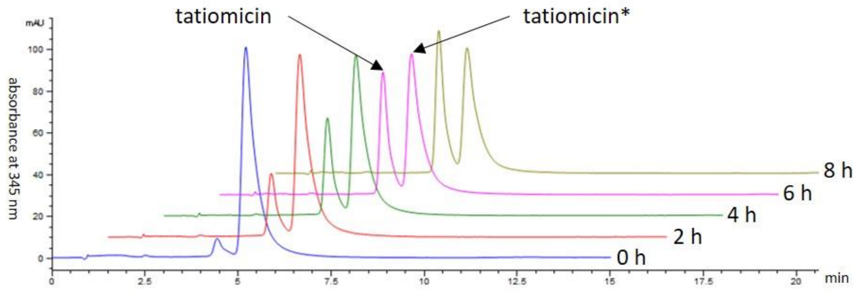

Figure S7 HPLC analysis of minor epimer of tatiomicin verses time showing equilibration with major epimer.

UV Absorption Spectra of Tatiomicin and Tatiomicin*

The UV/VIS spectra of tatiomicin and tatiomicin* were measured during HPLC separation of a mixture of the epimers of tatiomicin using an Agilent 1260 HPLC equipped with a fully wavelength UV/vis detector.

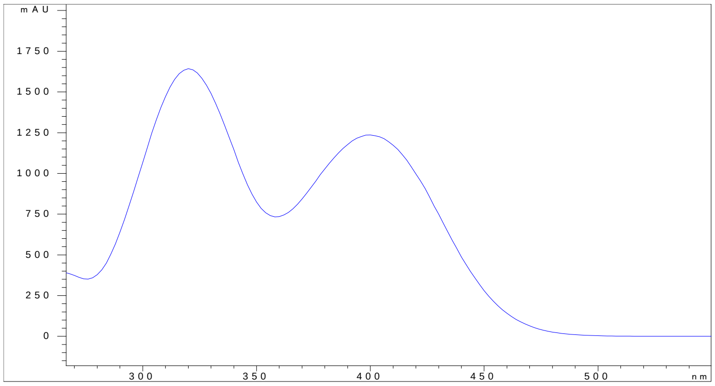

Figure S8 UV/vis absorption spectrum of tatiomicin

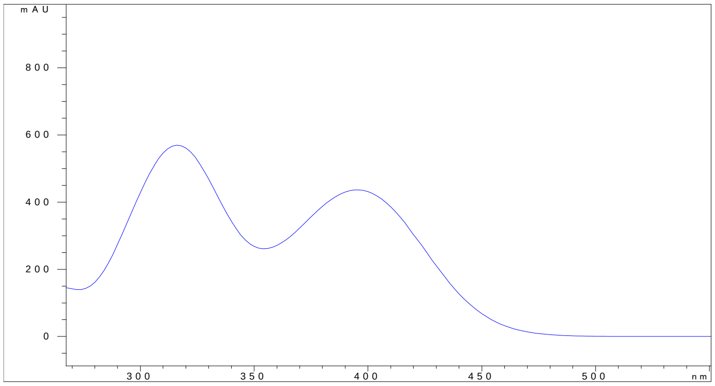

Figure S9 UV/vis absorption spectrum of tatiomicin*

LCMS of Tatiomicin and Tatiomicin*

Liquid chromatography - mass spectrometry (LCMS) experiments were performed on an Agilent HPLC 1260 – MicroTOF, using electrospray ionisation in positive mode. Both tatiomicin and tatiomicin* showed the presence of $[M+H]^+$ (403), $[M+Na]^+$ (425), $[2M+Na]^+$ (827) and $[3M+Na]^+$ (1229) ions.

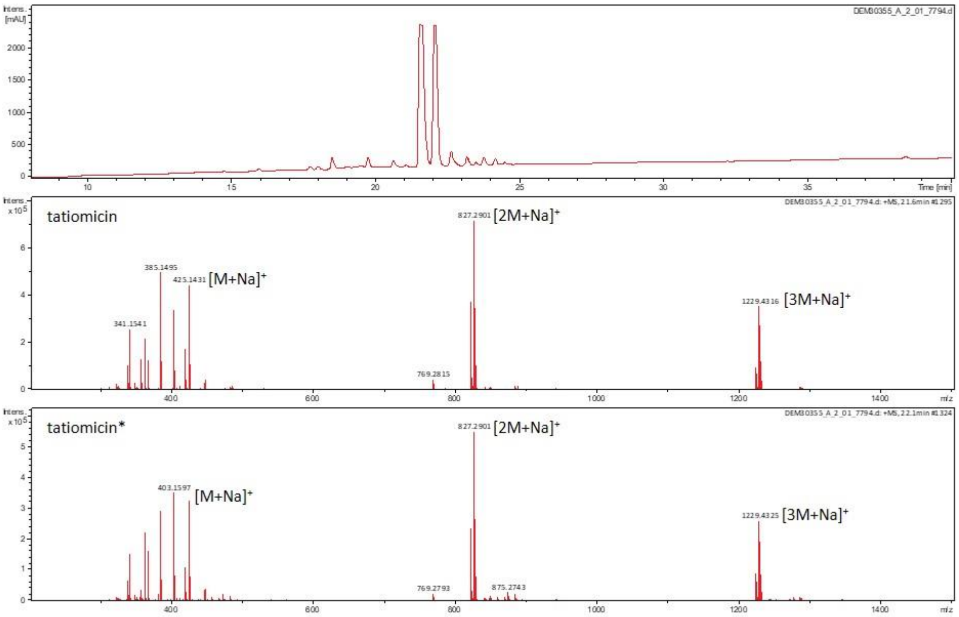

Figure S10 LCMS chromatogram (top) and mass spectra of tatiomicin (middle) and tatiomicin* (bottom).

Chemical Characterisation of Tatiomicin and Tatiomicin*

Table S2 NMR data for (–)-tatiomicin (**3**).

Position	δ_H (*J* in Hz)	δ_C	Position	δ_H (*J* in Hz)	δ_C
1	-	196.3, C	9a	-	129.0, C
2	2.78 q (7.6)	49.8, CH	10	5.49 s	66.5, CH
3	-	81.7, C	10a	-	118.9, C
4	-	82.7, C	11	5.47 dq (12.0, 2.0)	124.7, CH
4a	-	83.5, C	12	5.77 dq (12.0, 7.2)	133.2, CH
5	-	151.2, C	13	1.94 dd (7.2, 2.0)	15.6, CH_3
6	6.92 d, (9.2)	112.8, CH	14	-	174.9, C
7	7.05 d (9.2)	115.9, CH	15	1.20 d (7.6)	11.3, CH_3
8	-	152.9, C	16	3.84 s	56.4, CH_3
8a	-	123.3, C	17	3.85, s	56.5, CH_3
9	8.12 s	131.9, CH	-	-	-

Optical Rotation Tatiomicin

$[\alpha]_D$ -78 (c 0.3, CH_2Cl_2)

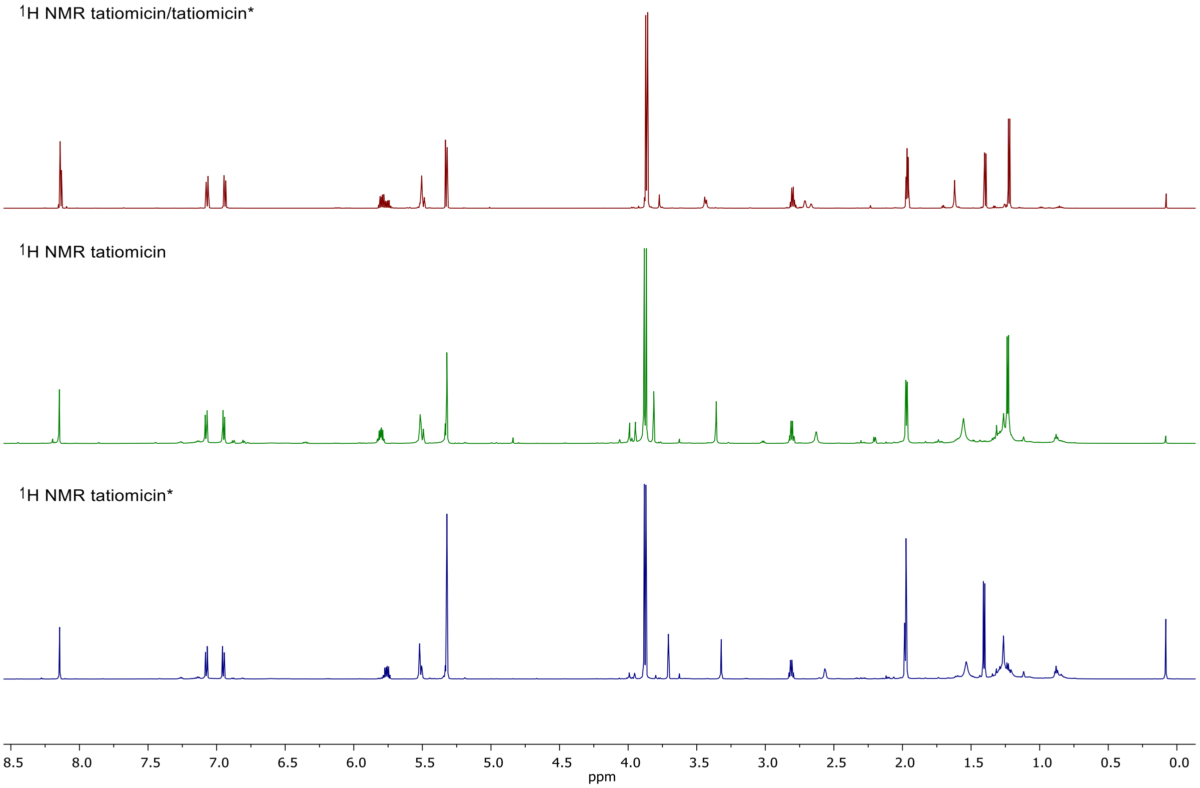

Figure S11 Comparison of ^{1}H NMR data of Tatiomicin vs Tatiomicin*

NMR data Tatiomicin/Tatiomicin*

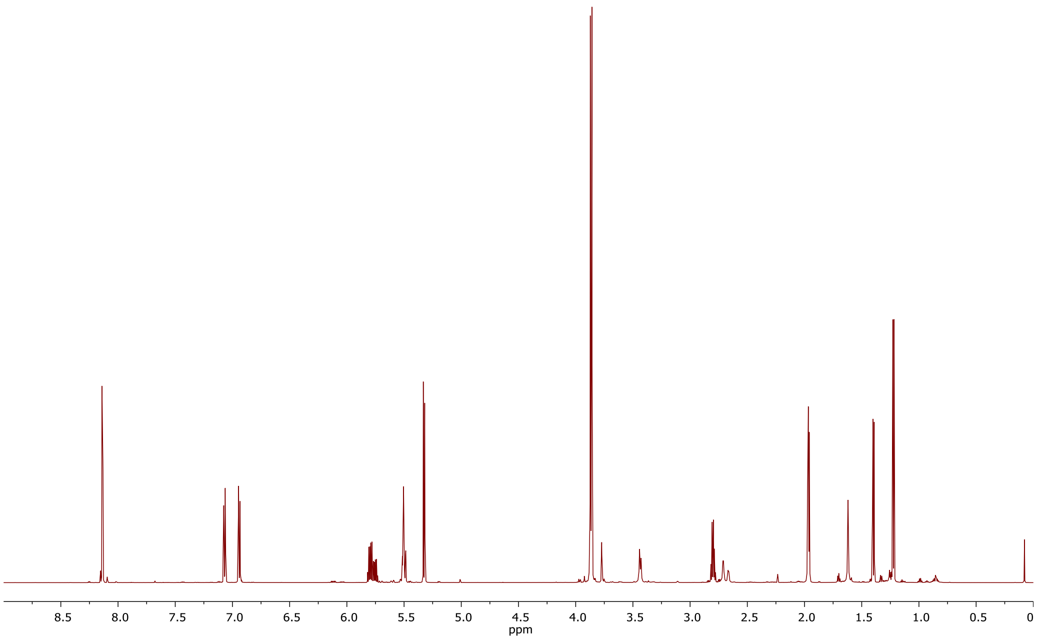

Figure S12 ^{1}H NMR 700 MHz (CD_2Cl_2)

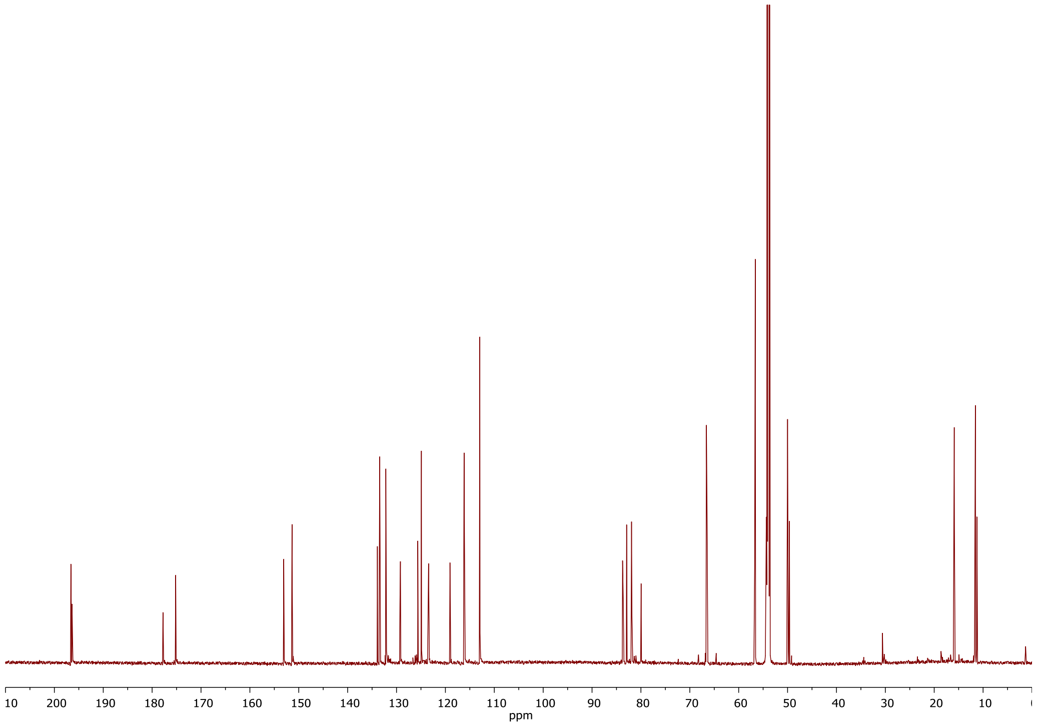

Figure S13 ^{13}C NMR 175 MHz (CD_2Cl_2)

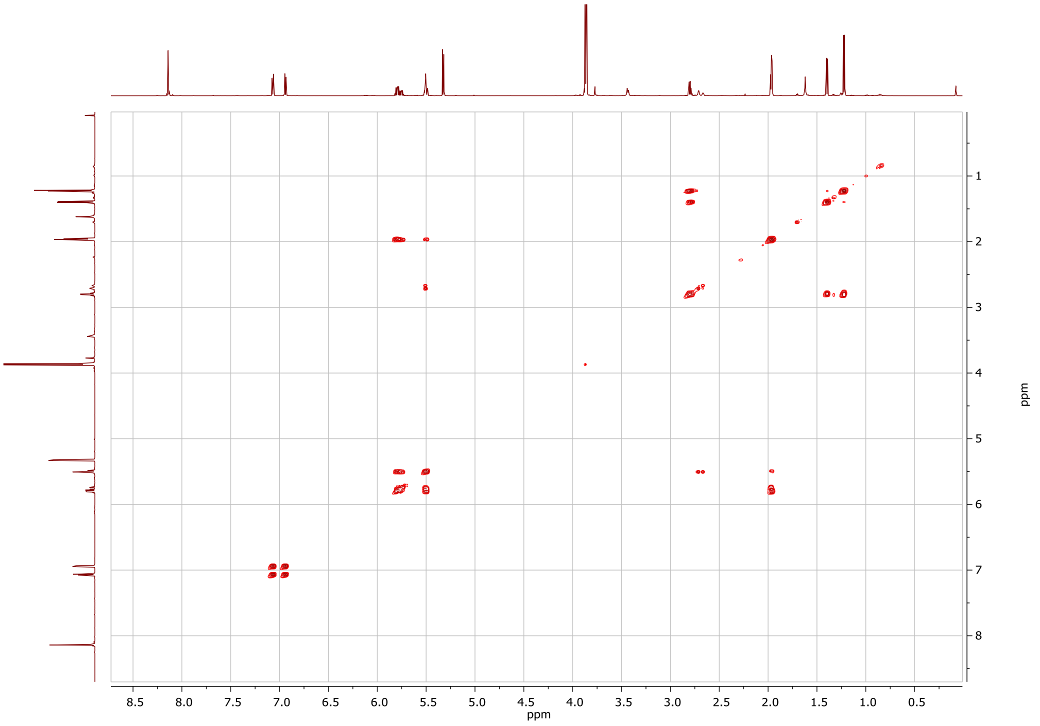

Figure S14 COSY (CD_2Cl_2)

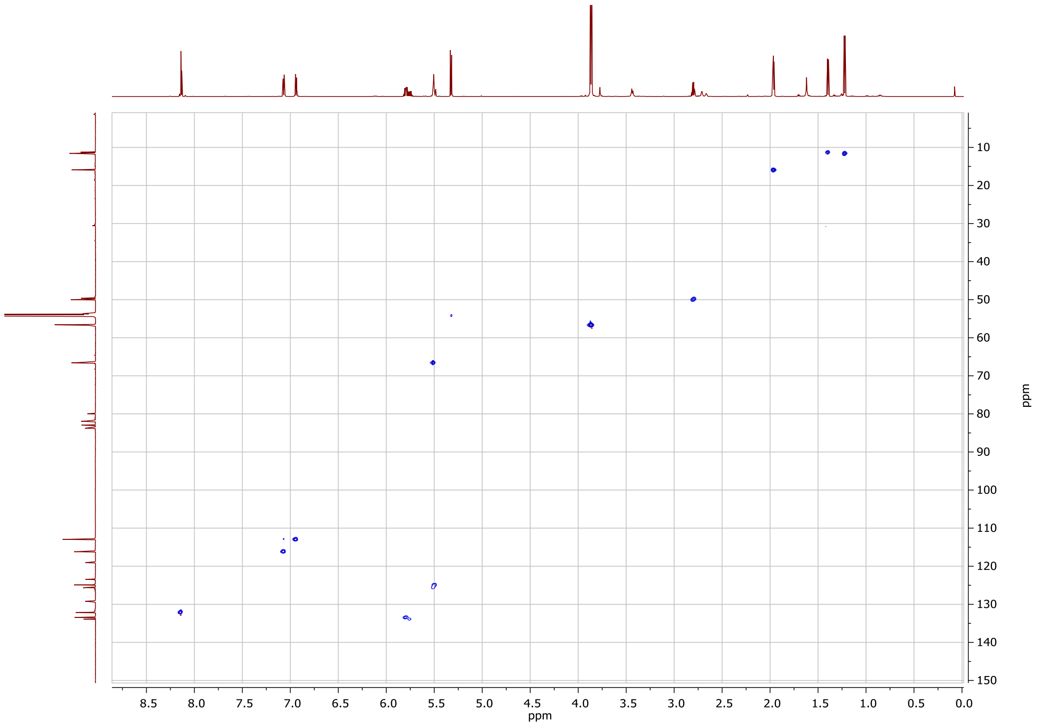

Figure S15 HSQC (CD_2Cl_2)

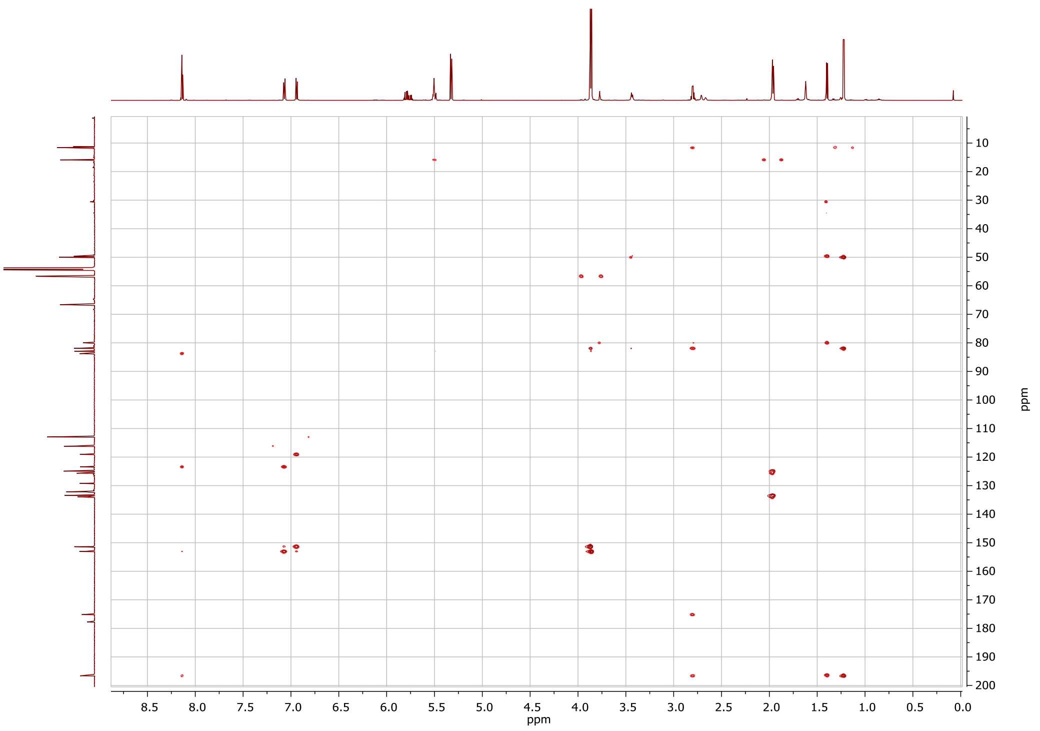

Figure S16 HMBC (CD_2Cl_2)

NMR data Tatiomicin

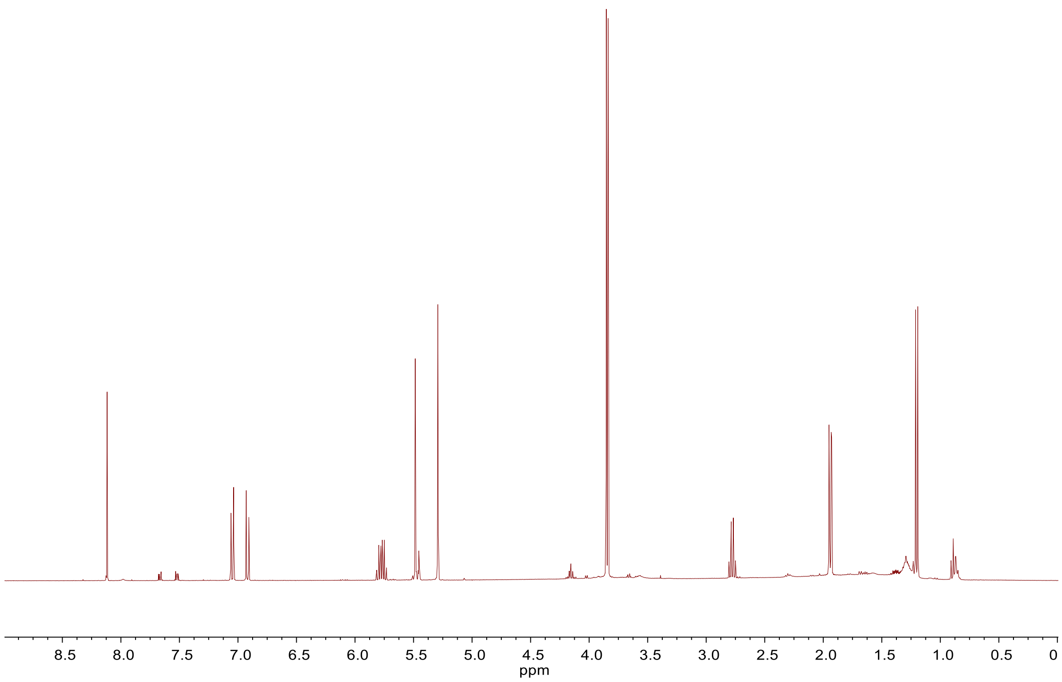

Figure S17 ^{1}H NMR 500 MHz (CD_2Cl_2)

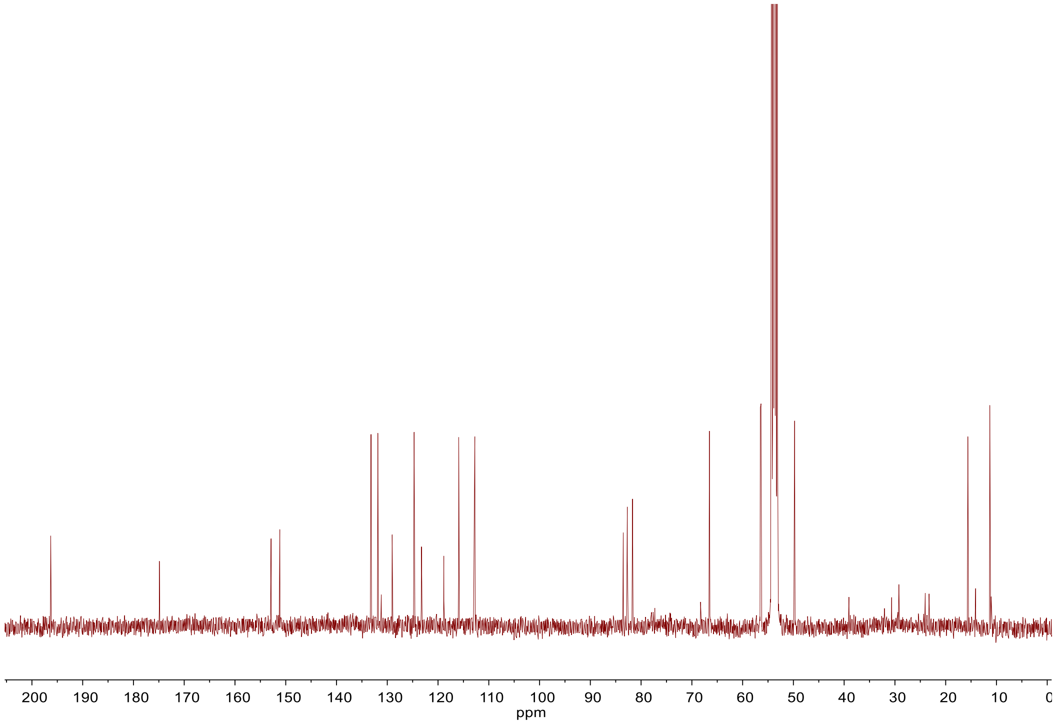

Figure S18 ^{13}C NMR 125 MHz (CD_2Cl_2)

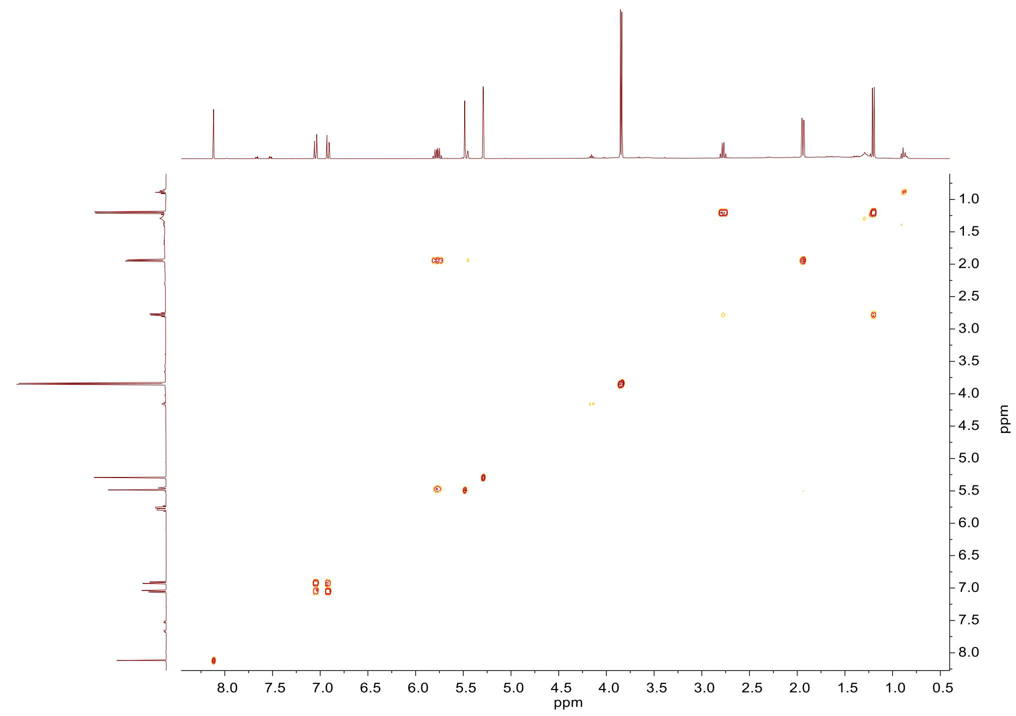

Figure S19 COSY (CD_2Cl_2)

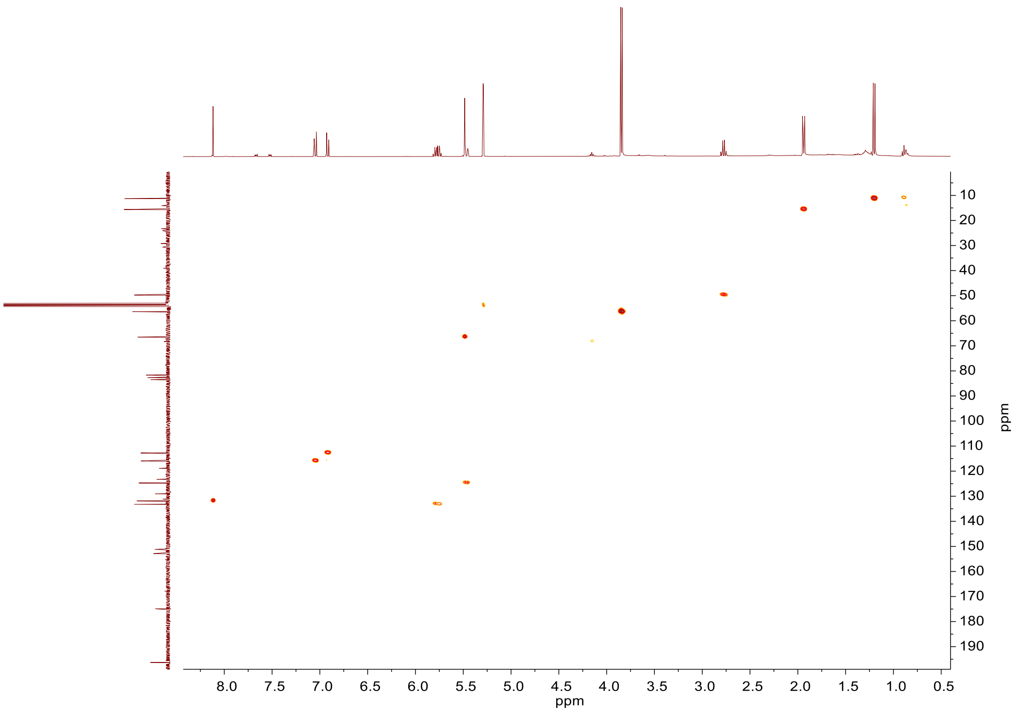

Figure S20 HSQC (CD_2Cl_2)

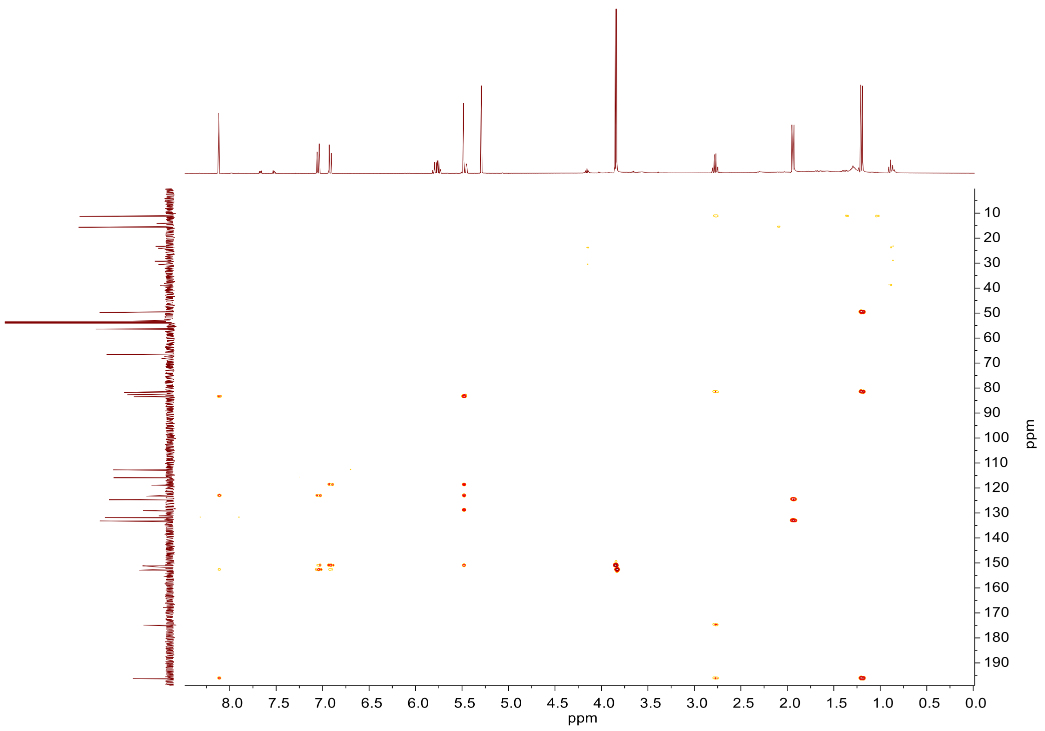

Figure S21 HMBC (CD_2Cl_2)

NMR data Tatiomicin*

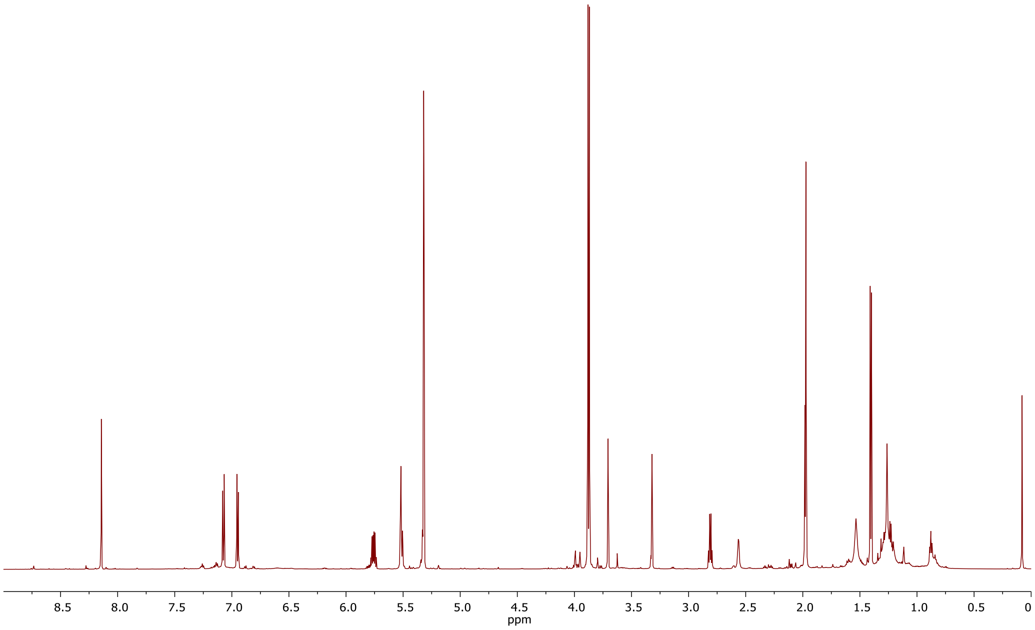

Figure S22 HMBC (CD_2Cl_2) ^{1}H NMR 700 MHz (CD_2Cl_2)

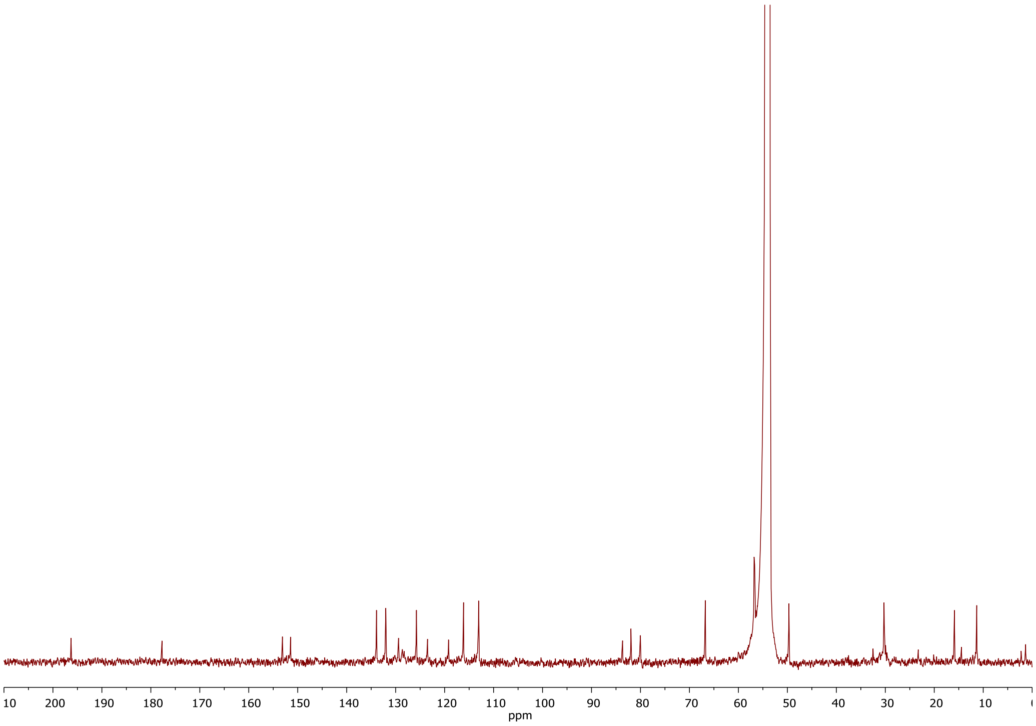

Figure S23 ^{13}C NMR 175 MHz (CD_2Cl_2)

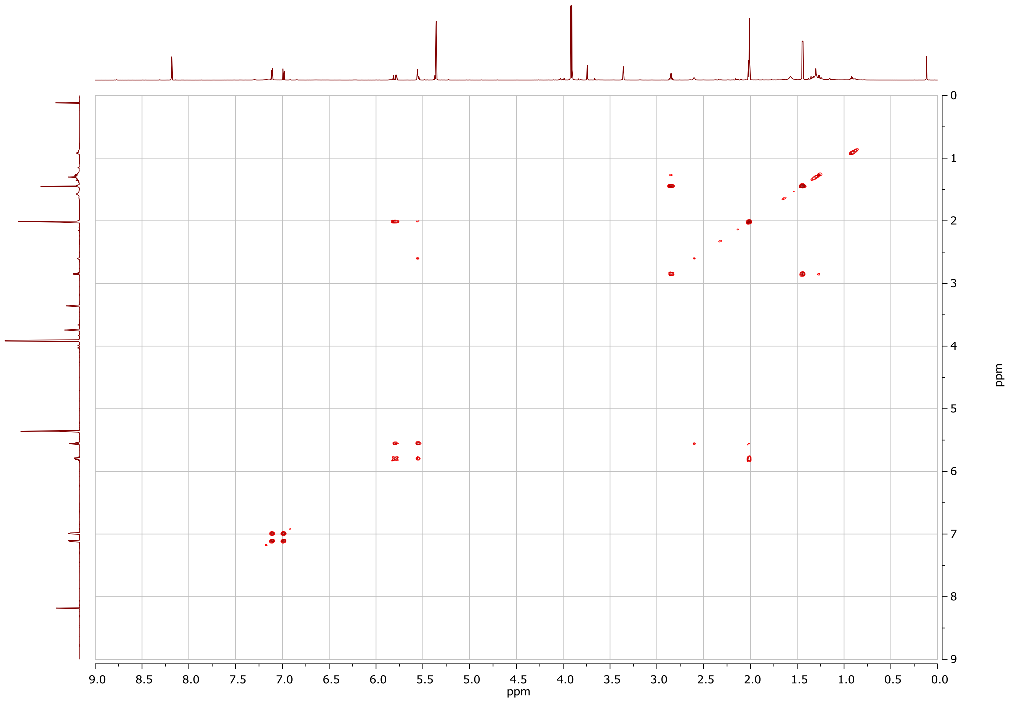

Figure S 24 COSY (CD_2Cl_2)

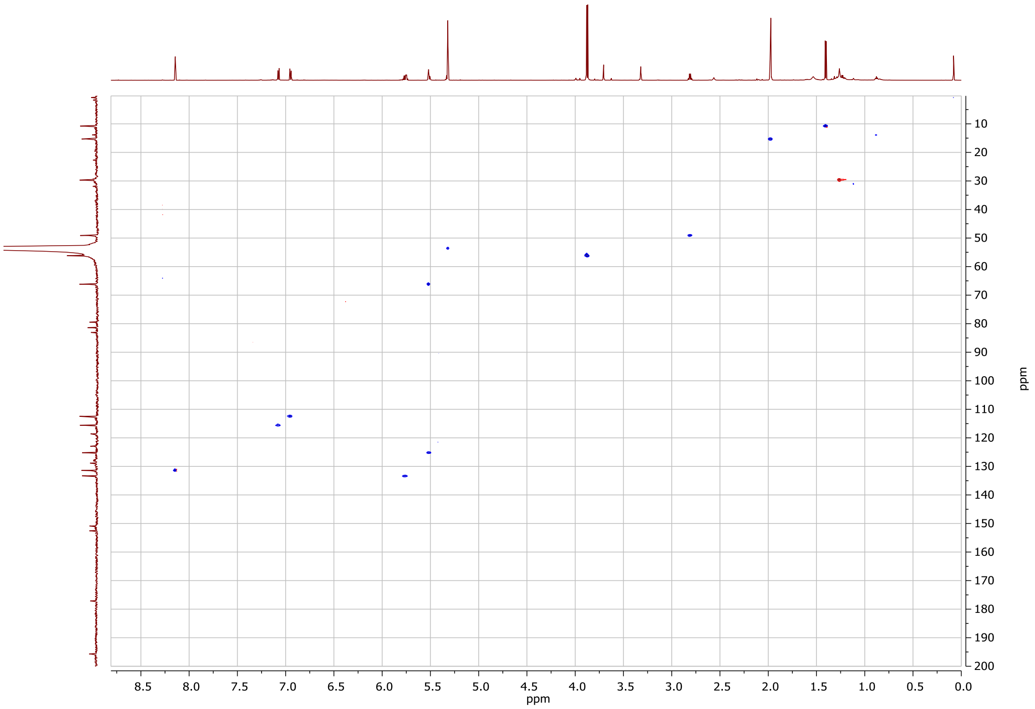

Figure S25 HSQC (CD_2Cl_2)

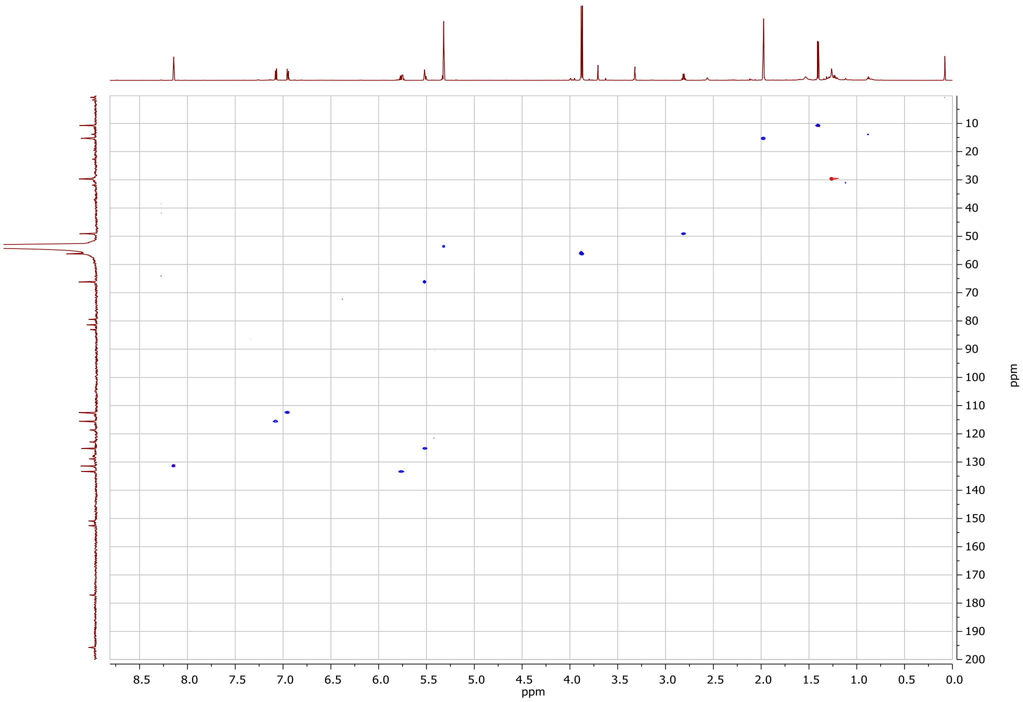

Figure S26 HMBC (CD_2Cl_2)

X-Ray Crystallographic Studies of Tatiomicin

An initial single-crystal X-ray diffraction experiment was conducted at Diamond Light Source beamline I19 prior to its major upgrade in 2016,[2] following in-house screening to demonstrate that synchrotron radiation was required for the very small crystals. The principal beamline equipment was a Crystal Logics kappa-geometry diffractometer fitted with a Rigaku Saturn 724+ CCD detector and controlled by Rigaku CrystalClear software. The sample temperature, maintained by an Oxford Cryosystems Cryostream nitrogen-gas cooler, was 150 K, and the X-ray wavelength was 0.6889 Å (standard beamline conditions, close to the Zr absorption edge and similar to the widely used Mo-$K\alpha$ wavelength). This wavelength does not permit determination of absolute configuration for a chiral molecule, the resonant scattering contributions being too small for compounds containing no elements heavier than oxygen; the configuration reported here is that confirmed subsequently in the second diffraction experiment with longer X-ray wavelength.

The structure was determined by standard direct methods and refined by least-squares techniques based on all unique measured data (refinement on F^2 values).[3, 4] The asymmetric unit consists of two independent tatiomicin molecules and one molecule of d_6-benzene; this solvent was used for recording NMR spectra prior to crystallization. All components are fully ordered. The data were of sufficient quality for O-bound H atoms to be refined freely, while C-bound H atoms were constrained with a riding model. Hydrogen bonding patterns were thus established and are included in the CIF-format results deposited in the Cambridge Structural Database. A summary of crystal data and refinement information is given below (Table S3).

In order to establish the absolute configuration experimentally, a further X-ray diffraction experiment was conducted following the I19 beamline upgrade. Another crystal from the same original sample was selected. The principal equipment of the upgraded beamline is a large custom-built 3-circle fixed-χ diffractometer fitted with a Dectris Pilatus 2M detector, controlled by Diamond Light Source GDA software.[5] The sample temperature (Cryostream) was 100 K, and the X-ray wavelength was 1.4879 Å (close to the Ni absorption edge, and similar to the widely used Cu-$K\alpha$ wavelength). This unusual experimental beamline arrangement requires a large number of individual scans with different detector positions in order to obtain a complete diffraction pattern with moderate multiplicity of observations.

Approximately 1/6 of the solvent molecules were lost from the crystal in the intervening period. In those locations, the nearby dimethoxy-substituted benzene ring of one of the tatiomicin molecules in the

asymmetric unit is slightly displaced towards the vacant solvent site. This has been modelled by inclusion of a minor disorder component for this part of the molecule. The refined occupancy factors for the major disorder component and the solvent molecule refined independently to essentially the same value, so they were subsequently set to be equal; this treatment is seen to be valid by recognition of unacceptably short contacts between the minor disorder component and the solvent molecule, meaning that they cannot both be present together. The disorder makes no significant difference to the molecular geometry; the difference between the major and minor components is mainly a small hinge movement about the C29…C30 axis in the central non-aromatic ring.

The principal purpose of this experiment was to establish the absolute configuration, which was indeterminate in the previous experiment with short-wavelength X-rays. This has been completely successful: the absolute-structure ('Flack') parameter[6-8] has a value of 0.05(6), insignificantly different from zero and with a small standard uncertainty, indicating the correct absolute configuration in the refined model structure (Table S4).

Detailed analysis of the molecular geometry is best done with the previous, fully ordered, crystal structure. The two structures are of comparable precision. Note that different temperatures were used (150 and 100 K). A summary of crystal data and refinement information for the second experiment is provided in Table S2 below. Methods and software were as for the first set of data. A listing of molecular geometrical parameters for the ordered structure is given below (Table S5).

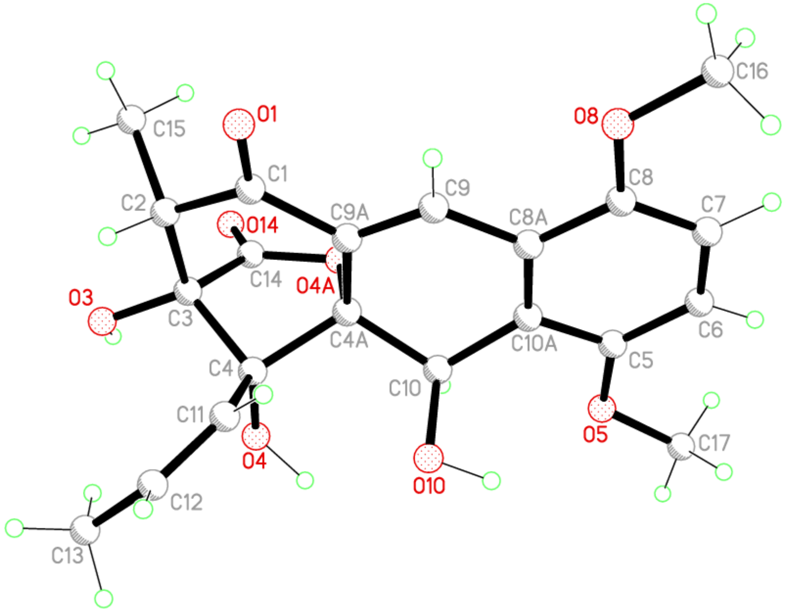

Figure S27 One of the two molecules of tatiomicin in the asymmetric unit

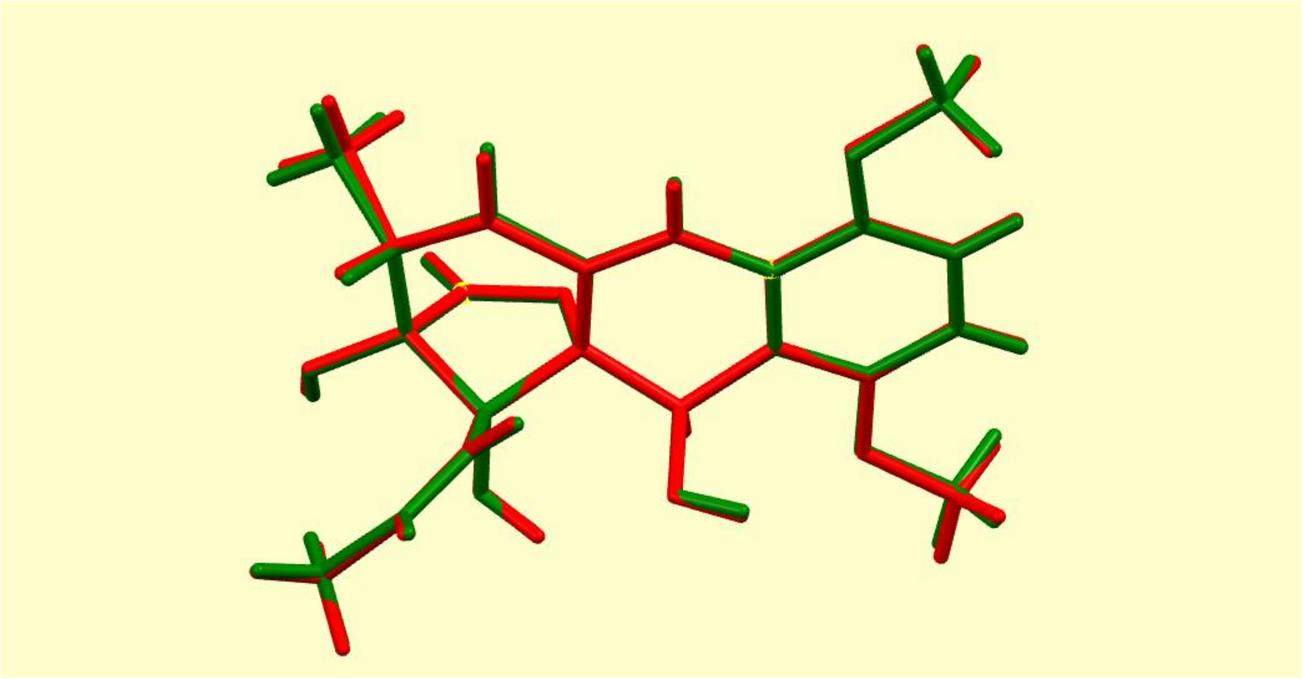

Figure S28 An overlay of the two molecules (red and green) in the asymmetric unit (ignoring the minor disorder component), showing that they have essentially the same conformation and the same absolute configuration (second experiment, at 100 K)

Crystallography Data Tables for Tatiomicin

Table S3. Crystal data and structure refinement from first experiment.

Identification code	mjh73	
Chemical formula (moiety)	$C_{21}H_{22}O_8 \cdot 0.5C_6D_6$	
Chemical formula (total)	$C_{24}H_{22}D_3O_8$	
Formula weight	444.46	
Temperature	150(2) K	
Radiation, wavelength	synchrotron, 0.6889 Å	
Crystal system, space group	orthorhombic, $P2_12_12_1$	
Unit cell parameters	a = 8.0333(13) Å	α = 90°
	b = 17.001(3) Å	β = 90°
	c = 32.193(5) Å	γ = 90°
Cell volume	4396.7(13) $Å^3$	
Z	8	
Calculated density	1.343 g/cm^3	
Absorption coefficient μ	0.060 mm^{-1}	
F(000)	1864	
Crystal colour and size	yellow, 0.100 $\times$ 0.020 $\times$ 0.020 mm^3	
Reflections for cell refinement	9992 (θ range 2.5 to 24.3°)	
Data collection method	Rigaku Saturn 724+ on kappa diffractometer<br>wide-frame ω scans	
θ range for data collection	1.2 to 24.4°	
Index ranges	h –9 to 9, k –20 to 20, l –38 to 38	
Completeness to θ = 24.4°	98.8 %	
Reflections collected	34672	
Independent reflections	7819 (R_{int} = 0.0676)	
Reflections with $F^2 > 2\sigma$	6274	
Absorption correction	none	
Structure solution	direct methods	
Refinement method	Full-matrix least-squares on F^2	
Weighting parameters a, b	0.0622, 0.1985	
Data / restraints / parameters	7819 / 0 / 610	
Final R indices [$F^2 > 2\sigma$]	R1 = 0.0423, wR2 = 0.1022	
R indices (all data)	R1 = 0.0589, wR2 = 0.1088	
Goodness-of-fit on F^2	1.036	
Absolute structure parameter	0.3(4)	
Extinction coefficient	0.031(3)	
Largest and mean shift/su	0.001 and 0.000	
Largest diff. peak and hole	0.18 and –0.20 e $Å^{-3}$	

Table S4. Crystal data and structure refinement from second experiment.

Identification code	mjh73	
Chemical formula (moiety)	$C_{21}H_{22}O_8 \cdot 0.42C_6D_6$	
Chemical formula (total)	$C_{23.5}H_{22}D_{2.5}O_8$	
Formula weight	437.44	
Temperature	100(2) K	
Radiation, wavelength	synchrotron, 1.4879 Å	
Crystal system, space group	orthorhombic, $P2_12_12_1$	
Unit cell parameters	a = 8.0059(3) Å	α = 90°
	b = 16.8978(7) Å	β = 90°
	c = 31.9515(13) Å	γ = 90°
Cell volume	4322.5(3) Å^3	
Z	8	
Calculated density	1.344 g/cm^3	
Absorption coefficient μ	0.760 mm^{-1}	
F(000)	1836	
Crystal colour and size	yellow, 0.042 $\times$ 0.031 $\times$ 0.028 mm^3	
Reflections for cell refinement	1717 (θ range 2.8 to 25.0°)	
Data collection method	fixed-χ diffractometer with Pilatus 2M detector	
	narrow-frame ω and ϕ scans	
θ range for data collection	2.7 to 65.8°	
Index ranges	h –9 to 9, k –20 to 20, l –39 to 39	
Completeness to θ = 63.2°	99.9 %	
Reflections collected	48250	
Independent reflections	8060 (R_{int} = 0.0920)	
Reflections with F^2>2σ	6155	
Absorption correction	none	
Structure solution	direct methods	
Refinement method	Full-matrix least-squares on F^2	
Weighting parameters a, b	0.0536,	
Data / restraints / parameters	8060 / 520 / 699	
Final R indices [F^2>2σ]	R1 = 0.0482, wR2 = 0.0940	
R indices (all data)	R1 = 0.0714, wR2 = 0.0969	
Goodness-of-fit on F^2	0.999	
Absolute structure parameter	0.05(6)	
Extinction coefficient	0.00065(12)	
Largest and mean shift/su	0.001 and 0.000	
Largest diff. peak and hole	0.18 and –0.17 e Å^{-3}	

Table S5. Bond lengths (Å), bond angles and torsion angles (°) for tatiomicin at 150 K

C1–C2	1.520(4)	C1–C9A	1.479(4)
C1–O1	1.221(4)	C2–H2	1.000
C2–C3	1.541(4)	C2–C15	1.533(5)
C3–C4	1.557(4)	C3–C14	1.511(5)
C3–O3	1.399(4)	C4A–C4	1.560(4)
C4A–C9A	1.500(4)	C4A–C10	1.540(4)
C4A–O4A	1.492(3)	C4–C11	1.516(4)
C4–O4	1.424(4)	C5–C6	1.389(5)
C5–C10A	1.407(5)	C5–O5	1.358(4)
C6–H6	0.950	C6–C7	1.377(6)
C7–H7	0.950	C7–C8	1.384(5)
C8A–C8	1.404(5)	C8A–C9	1.450(4)
C8A–C10A	1.400(5)	C8–O8	1.375(4)
C9A–C9	1.337(4)	C9–H9	0.950
C10A–C10	1.510(5)	C10–H10	1.000
C10–O10	1.437(4)	C11–H11	0.950
C11–C12	1.314(5)	C12–H12	0.950
C12–C13	1.491(5)	C13–H13A	0.980
C13–H13B	0.980	C13–H13C	0.980
C14–O4A	1.350(3)	C14–O14	1.201(4)
C15–H15A	0.980	C15–H15B	0.980
C15–H15C	0.980	C16–H16A	0.980
C16–H16B	0.980	C16–H16C	0.980
C16–O8	1.437(5)	C17–H17A	0.980
C17–H17B	0.980	C17–H17C	0.980
C17–O5	1.438(4)	O3–H3O	0.85(5)
O4–H4O	0.89(4)	O10–H10O	0.85(5)
C21–C22	1.520(5)	C21–C29A	1.472(5)
C21–O21	1.226(4)	C22–H22	1.000
C22–C23	1.549(4)	C22–C35	1.535(5)
C23–C24	1.560(4)	C23–C34	1.518(5)
C23–O23	1.398(4)	C24A–C24	1.561(4)
C24A–C29A	1.499(5)	C24A–C30	1.529(4)
C24A–O24A	1.489(4)	C24–C31	1.508(5)
C24–O24	1.429(4)	C25–C26	1.407(6)
C25–C30A	1.384(5)	C25–O25	1.364(5)
C26–H26	0.950	C26–C27	1.381(7)
C27–H27	0.950	C27–C28	1.377(7)
C28A–C28	1.417(5)	C28A–C29	1.436(5)
C28A–C30A	1.408(5)	C28–O28	1.377(6)
C29A–C29	1.341(5)	C29–H29	0.950
C30–H30	1.000	C30–C30A	1.510(5)
C30–O30	1.442(4)	C31–H31	0.950
C31–C32	1.325(5)	C32–H32	0.950
C32–C33	1.485(6)	C33–H33A	0.980
C33–H33B	0.980	C33–H33C	0.980

C34–O24A	1.336(4)	C34–O34	1.203(4)
C35–H35A	0.980	C35–H35B	0.980
C35–H35C	0.980	C36–H36A	0.980
C36–H36B	0.980	C36–H36C	0.980
C36–O28	1.442(5)	C37–H37A	0.980
C37–H37B	0.980	C37–H37C	0.980
C37–O25	1.418(5)	O23–H23O	0.86(5)
O24–H24O	0.90(5)	O30–H30O	0.90(5)
C41–D41	0.950	C41–C42	1.338(11)
C41–C46	1.359(10)	C42–D42	0.950
C42–C43	1.448(11)	C43–D43	0.950
C43–C44	1.361(11)	C44–D44	0.950
C44–C45	1.314(12)	C45–D45	0.950
C45–C46	1.346(11)	C46–D46	0.950

C2–C1–C9A	118.6(3)	C2–C1–O1	119.4(3)
C9A–C1–O1	122.0(3)	C1–C2–H2	106.6
C1–C2–C3	114.5(2)	C1–C2–C15	109.5(3)
H2–C2–C3	106.6	H2–C2–C15	106.6
C3–C2–C15	112.5(3)	C2–C3–C4	110.6(2)
C2–C3–C14	110.2(3)	C2–C3–O3	106.8(2)
C4–C3–C14	100.2(2)	C4–C3–O3	115.4(3)
C14–C3–O3	113.5(2)	C4–C4A–C9A	109.9(2)
C4–C4A–C10	115.9(2)	C4–C4A–O4A	102.9(2)
C9A–C4A–C10	115.0(2)	C9A–C4A–O4A	107.3(2)
C10–C4A–O4A	104.6(2)	C3–C4–C4A	97.2(2)
C3–C4–C11	116.4(3)	C3–C4–O4	104.6(2)
C4A–C4–C11	113.1(2)	C4A–C4–O4	110.4(2)
C11–C4–O4	113.7(2)	C6–C5–C10A	118.6(3)
C6–C5–O5	126.1(3)	C10A–C5–O5	115.2(3)
C5–C6–H6	119.3	C5–C6–C7	121.3(3)
H6–C6–C7	119.3	C6–C7–H7	119.7
C6–C7–C8	120.5(3)	H7–C7–C8	119.7
C8–C8A–C9	120.9(3)	C8–C8A–C10A	119.6(3)
C9–C8A–C10A	119.4(3)	C7–C8–C8A	119.6(3)
C7–C8–O8	125.1(3)	C8A–C8–O8	115.3(3)
C1–C9A–C4A	117.5(2)	C1–C9A–C9	120.6(3)
C4A–C9A–C9	121.6(3)	C8A–C9–C9A	122.8(3)
C8A–C9–H9	118.6	C9A–C9–H9	118.6
C5–C10A–C8A	120.2(3)	C5–C10A–C10	119.3(3)
C8A–C10A–C10	120.2(3)	C4A–C10–C10A	113.5(3)
C4A–C10–H10	108.8	C4A–C10–O10	106.3(2)
C10A–C10–H10	108.8	C10A–C10–O10	110.5(2)
H10–C10–O10	108.8	C4–C11–H11	115.8
C4–C11–C12	128.4(3)	H11–C11–C12	115.8
C11–C12–H12	115.4	C11–C12–C13	129.3(3)
H12–C12–C13	115.4	C12–C13–H13A	109.5
C12–C13–H13B	109.5	C12–C13–H13C	109.5

H13A–C13–H13B	109.5	H13A–C13–H13C	109.5
H13B–C13–H13C	109.5	C3–C14–O4A	109.5(2)
C3–C14–O14	128.8(3)	O4A–C14–O14	121.7(3)
C2–C15–H15A	109.5	C2–C15–H15B	109.5
C2–C15–H15C	109.5	H15A–C15–H15B	109.5
H15A–C15–H15C	109.5	H15B–C15–H15C	109.5
H16A–C16–H16B	109.5	H16A–C16–H16C	109.5
H16A–C16–O8	109.5	H16B–C16–H16C	109.5
H16B–C16–O8	109.5	H16C–C16–O8	109.5
H17A–C17–H17B	109.5	H17A–C17–H17C	109.5
H17A–C17–O5	109.5	H17B–C17–H17C	109.5
H17B–C17–O5	109.5	H17C–C17–O5	109.5
C3–O3–H3O	100(3)	C4–O4–H4O	108(3)
C4A–O4A–C14	108.7(2)	C5–O5–C17	117.5(3)
C8–O8–C16	118.2(3)	C10–O10–H10O	100(3)
C22–C21–C29A	119.2(3)	C22–C21–O21	119.0(3)
C29A–C21–O21	121.8(3)	C21–C22–H22	106.2
C21–C22–C23	114.4(3)	C21–C22–C35	111.0(3)
H22–C22–C23	106.2	H22–C22–C35	106.2
C23–C22–C35	112.3(3)	C22–C23–C24	109.5(2)
C22–C23–C34	110.3(3)	C22–C23–O23	107.4(3)
C24–C23–C34	100.4(2)	C24–C23–O23	115.9(3)
C34–C23–O23	113.2(3)	C24–C24A–C29A	110.1(3)
C24–C24A–C30	114.9(3)	C24–C24A–O24A	103.2(2)
C29A–C24A–C30	115.5(3)	C29A–C24A–O24A	107.4(2)
C30–C24A–O24A	104.5(2)	C23–C24–C24A	97.0(2)
C23–C24–C31	117.3(3)	C23–C24–O24	104.3(2)
C24A–C24–C31	113.4(2)	C24A–C24–O24	110.3(2)
C31–C24–O24	113.2(2)	C26–C25–C30A	118.7(4)
C26–C25–O25	125.7(4)	C30A–C25–O25	115.5(3)
C25–C26–H26	119.5	C25–C26–C27	121.0(4)
H26–C26–C27	119.5	C26–C27–H27	119.7
C26–C27–C28	120.6(4)	H27–C27–C28	119.7
C28–C28A–C29	120.6(4)	C28–C28A–C30A	119.1(4)
C29–C28A–C30A	120.1(3)	C27–C28–C28A	119.7(4)
C27–C28–O28	124.9(4)	C28A–C28–O28	115.4(4)
C21–C29A–C24A	117.3(3)	C21–C29A–C29	121.1(3)
C24A–C29A–C29	121.3(3)	C28A–C29–C29A	122.7(3)
C28A–C29–H29	118.7	C29A–C29–H29	118.7
C24A–C30–H30	108.8	C24A–C30–C30A	113.6(3)
C24A–C30–O30	107.2(3)	H30–C30–C30A	108.8
H30–C30–O30	108.8	C30A–C30–O30	109.7(2)
C25–C30A–C28A	120.8(3)	C25–C30A–C30	119.6(4)
C28A–C30A–C30	119.2(3)	C24–C31–H31	115.9
C24–C31–C32	128.2(3)	H31–C31–C32	115.9
C31–C32–H32	115.6	C31–C32–C33	128.7(4)
H32–C32–C33	115.6	C32–C33–H33A	109.5
C32–C33–H33B	109.5	C32–C33–H33C	109.5

H33A–C33–H33B	109.5	H33A–C33–H33C	109.5
H33B–C33–H33C	109.5	C23–C34–O24A	109.7(3)
C23–C34–O34	127.6(3)	O24A–C34–O34	122.6(3)
C22–C35–H35A	109.5	C22–C35–H35B	109.5
C22–C35–H35C	109.5	H35A–C35–H35B	109.5
H35A–C35–H35C	109.5	H35B–C35–H35C	109.5
H36A–C36–H36B	109.5	H36A–C36–H36C	109.5
H36A–C36–O28	109.5	H36B–C36–H36C	109.5
H36B–C36–O28	109.5	H36C–C36–O28	109.5
H37A–C37–H37B	109.5	H37A–C37–H37C	109.5
H37A–C37–O25	109.5	H37B–C37–H37C	109.5
H37B–C37–O25	109.5	H37C–C37–O25	109.5
C23–O23–H23O	105(3)	C24–O24–H24O	104(3)
C24A–O24A–C34	109.0(2)	C25–O25–C37	117.5(4)
C28–O28–C36	117.9(4)	C30–O30–H30O	106(3)
D41–C41–C42	120.4	D41–C41–C46	120.4
C42–C41–C46	119.2(7)	C41–C42–D42	120.4
C41–C42–C43	119.3(7)	D42–C42–C43	120.4
C42–C43–D43	121.4	C42–C43–C44	117.1(7)
D43–C43–C44	121.4	C43–C44–D44	118.8
C43–C44–C45	122.4(8)	D44–C44–C45	118.8
C44–C45–D45	120.2	C44–C45–C46	119.6(8)
D45–C45–C46	120.2	C41–C46–C45	122.4(9)
C41–C46–D46	118.8	C45–C46–D46	118.8

C9A–C1–C2–C3	5.8(4)	C9A–C1–C2–C15	–121.6(3)
O1–C1–C2–C3	–175.9(3)	O1–C1–C2–C15	56.7(4)
C1–C2–C3–C4	36.1(4)	C1–C2–C3–C14	–73.8(3)
C1–C2–C3–O3	162.5(3)	C15–C2–C3–C4	162.0(3)
C15–C2–C3–C14	52.1(3)	C15–C2–C3–O3	–71.7(3)
C2–C3–C4–C4A	–73.0(3)	C2–C3–C4–C11	47.3(4)
C2–C3–C4–O4	173.7(2)	C14–C3–C4–C4A	43.3(3)
C14–C3–C4–C11	163.6(3)	C14–C3–C4–O4	–70.0(3)
O3–C3–C4–C4A	165.7(2)	O3–C3–C4–C11	–74.1(3)
O3–C3–C4–O4	52.3(3)	C9A–C4A–C4–C3	73.2(3)
C9A–C4A–C4–C11	–49.5(3)	C9A–C4A–C4–O4	–178.2(2)
C10–C4A–C4–C3	–154.4(2)	C10–C4A–C4–C11	82.9(3)
C10–C4A–C4–O4	–45.8(3)	O4A–C4A–C4–C3	–40.9(3)
O4A–C4A–C4–C11	–163.6(2)	O4A–C4A–C4–O4	67.7(3)
C10A–C5–C6–C7	0.6(5)	O5–C5–C6–C7	–177.6(3)
C5–C6–C7–C8	–0.6(6)	C6–C7–C8–C8A	–0.5(5)
C6–C7–C8–O8	179.4(3)	C9–C8A–C8–C7	–174.7(3)
C9–C8A–C8–O8	5.5(4)	C10A–C8A–C8–C7	1.6(5)
C10A–C8A–C8–O8	–178.2(3)	C2–C1–C9A–C4A	–5.3(4)
C2–C1–C9A–C9	169.5(3)	O1–C1–C9A–C4A	176.5(3)
O1–C1–C9A–C9	–8.7(5)	C4–C4A–C9A–C1	–37.0(3)
C4–C4A–C9A–C9	148.2(3)	C10–C4A–C9A–C1	–169.9(3)

C10–C4A–C9A–C9	15.3(4)	O4A–C4A–C9A–C1	74.2(3)
O4A–C4A–C9A–C9	−100.6(3)	C1–C9A–C9–C8A	−171.2(3)
C4A–C9A–C9–C8A	3.4(4)	C8–C8A–C9–C9A	170.3(3)
C10A–C8A–C9–C9A	−6.0(4)	C8–C8A–C10A–C5	−1.7(4)
C8–C8A–C10A–C10	172.1(3)	C9–C8A–C10A–C5	174.7(3)
C9–C8A–C10A–C10	−11.5(4)	C6–C5–C10A–C8A	0.6(5)
C6–C5–C10A–C10	−173.3(3)	O5–C5–C10A–C8A	178.9(3)
O5–C5–C10A–C10	5.1(4)	C5–C10A–C10–C4A	−157.0(3)
C5–C10A–C10–O10	83.7(3)	C8A–C10A–C10–C4A	29.1(4)
C8A–C10A–C10–O10	−90.2(3)	C4–C4A–C10–C10A	−160.0(3)
C4–C4A–C10–O10	−38.4(3)	C9A–C4A–C10–C10A	−30.0(4)
C9A–C4A–C10–O10	91.6(3)	O4A–C4A–C10–C10A	87.4(3)
O4A–C4A–C10–O10	−150.9(2)	C3–C4–C11–C12	79.0(4)
C4A–C4–C11–C12	−169.6(3)	O4–C4–C11–C12	−42.6(5)
C4–C11–C12–C13	−0.2(7)	C2–C3–C14–O4A	83.7(3)
C2–C3–C14–O14	−95.6(4)	C4–C3–C14–O4A	−32.9(3)
C4–C3–C14–O14	147.8(3)	O3–C3–C14–O4A	−156.5(2)
O3–C3–C14–O14	24.2(5)	C3–C14–O4A–C4A	6.2(3)
O14–C14–O4A–C4A	−174.4(3)	C4–C4A–O4A–C14	23.1(3)
C9A–C4A–O4A–C14	−92.8(3)	C10–C4A–O4A–C14	144.6(2)
C6–C5–O5–C17	7.0(5)	C10A–C5–O5–C17	−171.1(3)
C7–C8–O8–C16	−0.6(5)	C8A–C8–O8–C16	179.2(3)
C29A–C21–C22–C23	−1.2(5)	C29A–C21–C22–C35	−129.6(3)
O21–C21–C22–C23	178.1(3)	O21–C21–C22–C35	49.8(5)
C21–C22–C23–C24	40.4(4)	C21–C22–C23–C34	−69.1(4)
C21–C22–C23–O23	167.1(3)	C35–C22–C23–C24	168.1(3)
C35–C22–C23–C34	58.5(4)	C35–C22–C23–O23	−65.3(4)
C22–C23–C24–C24A	−73.8(3)	C22–C23–C24–C31	47.1(4)
C22–C23–C24–O24	173.1(3)	C34–C23–C24–C24A	42.3(3)
C34–C23–C24–C31	163.1(3)	C34–C23–C24–O24	−70.9(3)
O23–C23–C24–C24A	164.6(3)	O23–C23–C24–C31	−74.6(4)
O23–C23–C24–O24	51.5(3)	C29A–C24A–C24–C23	73.9(3)
C29A–C24A–C24–C31	−49.9(3)	C29A–C24A–C24–O24	−178.0(2)
C30–C24A–C24–C23	−153.6(3)	C30–C24A–C24–C31	82.6(3)
C30–C24A–C24–O24	−45.5(3)	O24A–C24A–C24–C23	−40.5(3)
O24A–C24A–C24–C31	−164.3(2)	O24A–C24A–C24–O24	67.6(3)
C30A–C25–C26–C27	1.9(6)	O25–C25–C26–C27	−176.6(4)
C25–C26–C27–C28	−1.7(7)	C26–C27–C28–C28A	−0.3(6)
C26–C27–C28–O28	178.5(4)	C29–C28A–C28–C27	−173.2(4)
C29–C28A–C28–O28	7.9(5)	C30A–C28A–C28–C27	2.0(5)
C30A–C28A–C28–O28	−176.9(3)	C22–C21–C29A–C24A	1.0(4)
C22–C21–C29A–C29	173.8(3)	O21–C21–C29A–C24A	−178.3(3)
O21–C21–C29A–C29	−5.6(5)	C24–C24A–C29A–C21	−40.1(4)
C24–C24A–C29A–C29	147.2(3)	C30–C24A–C29A–C21	−172.2(3)
C30–C24A–C29A–C29	15.0(4)	O24A–C24A–C29A–C21	71.6(3)
O24A–C24A–C29A–C29	−101.2(3)	C21–C29A–C29–C28A	−169.5(3)

C24A–C29A–C29–C28A	2.9(5)	C28–C28A–C29–C29A	171.0(3)
C30A–C28A–C29–C29A	–4.1(5)	C24–C24A–C30–C30A	–160.0(3)
C24–C24A–C30–O30	–38.7(3)	C29A–C24A–C30–C30A	–30.1(4)
C29A–C24A–C30–O30	91.2(3)	O24A–C24A–C30–C30A	87.7(3)
O24A–C24A–C30–O30	–151.0(2)	C26–C25–C30A–C28A	–0.2(5)
C26–C25–C30A–C30	–173.2(3)	O25–C25–C30A–C28A	178.5(3)
O25–C25–C30A–C30	5.5(5)	C28–C28A–C30A–C25	–1.7(5)
C28–C28A–C30A–C30	171.3(3)	C29–C28A–C30A–C25	173.5(3)
C29–C28A–C30A–C30	–13.5(5)	C24A–C30–C30A–C25	–156.8(3)
C24A–C30–C30A–C28A	30.0(4)	O30–C30–C30A–C25	83.3(4)
O30–C30–C30A–C28A	–89.8(4)	C23–C24–C31–C32	77.2(4)
C24A–C24–C31–C32	–171.0(3)	O24–C24–C31–C32	–44.4(5)
C24–C31–C32–C33	–1.4(7)	C22–C23–C34–O24A	83.8(3)
C22–C23–C34–O34	–96.4(4)	C24–C23–C34–O24A	–31.7(3)
C24–C23–C34–O34	148.1(3)	O23–C23–C34–O24A	–155.9(3)
O23–C23–C34–O34	23.9(5)	C23–C34–O24A–C24A	5.2(3)
O34–C34–O24A–C24A	–174.7(3)	C24–C24A–O24A–C34	23.7(3)
C29A–C24A–O24A–C34	–92.6(3)	C30–C24A–O24A–C34	144.2(3)
C26–C25–O25–C37	5.7(6)	C30A–C25–O25–C37	–172.9(4)
C27–C28–O28–C36	8.4(6)	C28A–C28–O28–C36	–172.8(4)
C46–C41–C42–C43	1.0(10)	C41–C42–C43–C44	–1.3(9)
C42–C43–C44–C45	1.9(11)	C43–C44–C45–C46	–2.2(15)
C44–C45–C46–C41	1.8(15)	C42–C41–C46–C45	–1.3(13)

Hydrogen bonds for (–)-tatiomicin [Å and °].

D–H...A	d(D–H)	d(H...A)	d(D...A)	<(DHA)
O3–H3O...O14	0.85(5)	2.47(5)	2.880(3)	110(4)
O3–H3O...O24a	0.85(5)	2.01(5)	2.781(3)	150(4)
O4–H4O...O10	0.89(4)	2.33(4)	2.948(3)	126(3)
O4–H4O...O30	0.89(4)	2.13(4)	2.809(3)	133(3)
O10–H10O...O14b	0.85(5)	1.99(5)	2.834(3)	169(4)
O23–H23O...O34	0.86(5)	2.48(4)	2.859(4)	107(3)
O23–H23O...O4b	0.86(5)	2.01(5)	2.834(3)	159(4)
O24–H24O...O10	0.90(5)	2.23(5)	2.865(3)	127(4)
O24–H24O...O30	0.90(5)	2.30(5)	2.930(3)	126(3)
O30–H30O...O34a	0.90(5)	1.91(5)	2.814(3)	174(4)

Symmetry operations for equivalent atoms
a x–1,y,z b x+1,y,z

Computational Methods

Conformers of Tatiomicin

Table S6 Relative enthalpies (ΔH_{298K}) and the corresponding Boltzmann weights of tatiomicin calculated within the IEFPCM of chloroform.

Conformer	ΔH_{298K}[a] [kcal mol^{-1}]	Pop-ΔH [%]
**1**	0.00	77.1
**2**	0.89	17.2
**3**	1.65	4.8
**4**	3.17	0.4
**5**	3.26	0.3
**6**	3.42	0.2

[a]Referenced to H = -1414.649964 hartree

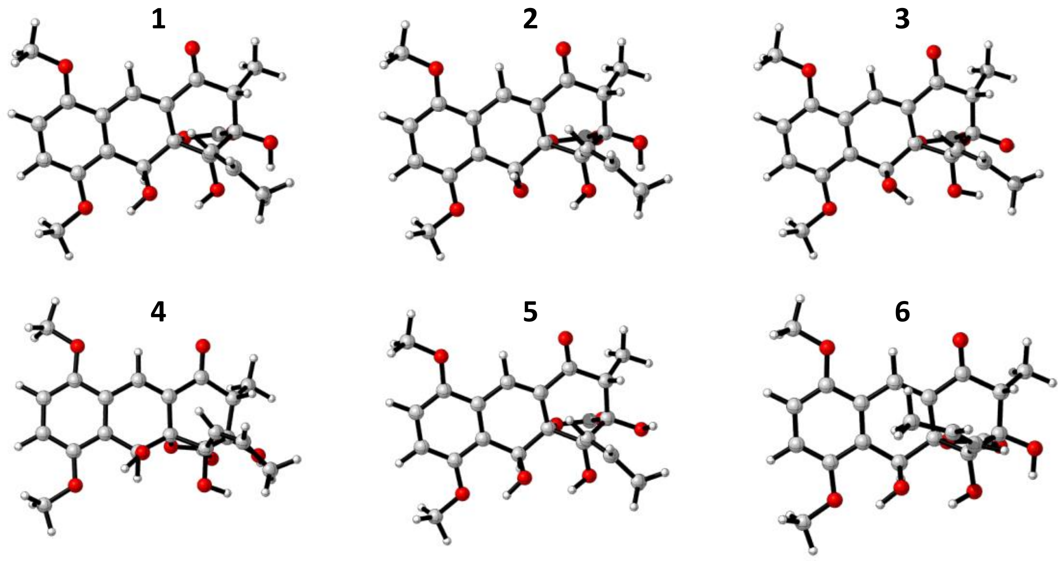

Figure S29 3D representations of the optimized structures, prepared using CYLview[9]

Conformers of Tatiomicin*

Table S7 Relative enthalpies (ΔH_{298K}) and the corresponding Boltzmann weights of the minor C-2 epimer of (-) **3** calculated within the IEFPCM of chloroform.

Conformer	ΔH_{298K}[a] [kcal mol^{-1}]	Pop-ΔH [%]
**1**	0.00	83.1
**2**	1.19	11.1
**3**	1.73	4.5
**4**	3.05	0.5
**5**	3.29	0.3
**6**	3.33	0.3
**7**	3.73	0.2

[a]Referenced to H = -1415.199655 hartree

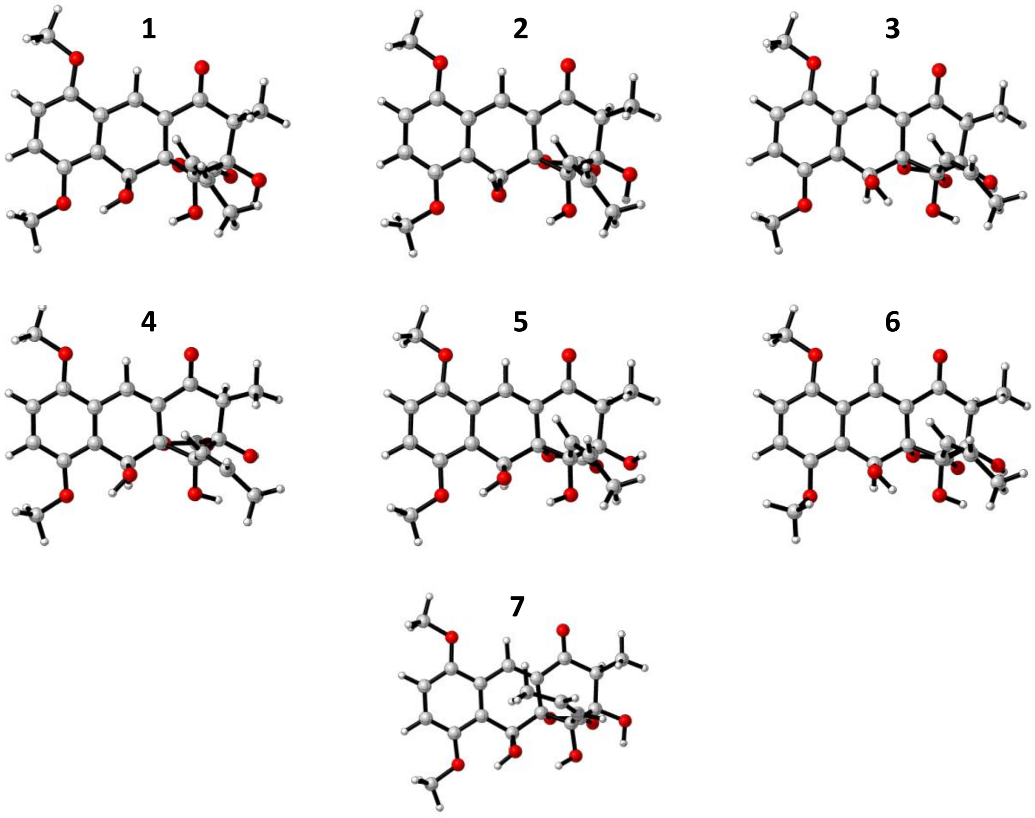

Figure S30 3D representations of the optimized structures were prepared using CYLview[9]

Experimental and Calculated VCD and ECD Spectra

IR and VCD spectra were recorded on a dual PEM ChiralIR-2X spectrometer (Biotools Inc., Jupiter, FL). All measurements were performed in $CDCl_3$ with a concentration of 0.1 M. A cell with 100 µm path length and BaF_2 windows was used. Both the sample and the solvent spectrum were recorded with a resolution of 4 cm^{-1}, totaling 30 000 scans each with both PEMs optimized at 1400 cm^{-1}. The final baseline-corrected VCD spectrum was obtained through subtraction of the solvent spectrum.

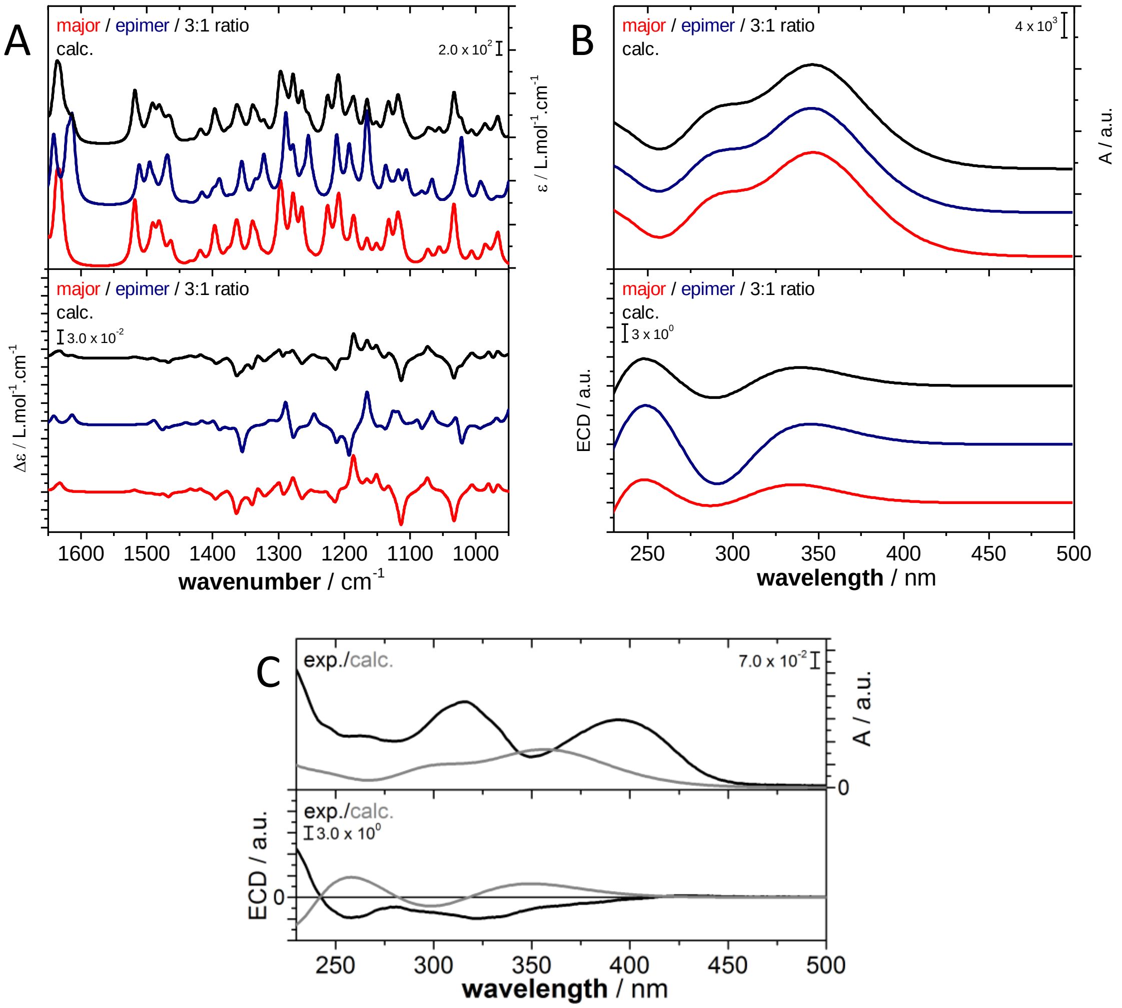

Figure S31 Calculated VCD (A) and ECD (B) spectra for tatiomicin, tatiomicin* and a 3:1 mixture of tatiomicin:tatiomicin* as found in isolated samples, and comparison between experimental and calculated ECD (C).

Table S8 Cartesian coordinates of calculated conformers

Tatiomicin **(3)** Conformer 1

C	-4.54314	0.45963	0.12473
C	-4.36309	-0.89022	-0.17258
C	-3.08259	-1.41420	-0.31674
C	-1.96787	-0.58164	-0.17678
C	-2.14060	0.77010	0.13898
C	-3.44580	1.29724	0.28956
C	-0.59839	-1.19424	-0.34070
C	0.57996	-0.20039	-0.37311
C	0.27882	1.19116	0.10272
C	-0.98415	1.61526	0.30441
C	1.89619	-0.72739	0.28539
C	2.88964	0.17544	-0.49521
C	2.83098	1.62665	0.01311
C	1.40912	2.13924	0.25513
O	-3.52976	2.61381	0.58639
O	-2.80845	-2.72887	-0.56795
C	-4.81748	3.19184	0.74988
C	-3.89534	-3.61790	-0.79928
O	-0.35912	-2.16043	0.69191
O	1.23602	3.30064	0.58165
C	1.91837	-0.58439	1.78416
C	2.72983	-1.18146	2.66308
C	3.83975	-2.15803	2.44641
O	2.19019	-2.03478	-0.17211
O	4.21522	-0.25021	-0.43785
C	3.63865	2.60749	-0.83907
O	0.95410	-0.08145	-1.79352
C	2.28855	0.07184	-1.90473
O	2.85025	0.11377	-2.96316
H	-5.55137	0.83872	0.22901
H	-5.23643	-1.52042	-0.28375
H	-0.57246	-1.70552	-1.30959
H	-1.13827	2.64778	0.59700
H	3.28493	1.58498	1.01297
H	-4.64377	4.24137	0.98089
H	-5.40499	3.11476	-0.17059
H	-5.35975	2.72232	1.57690
H	-3.44763	-4.58762	-1.00980
H	-4.53595	-3.69706	0.08450
H	-4.48723	-3.29563	-1.66122
H	-1.05831	-2.82207	0.59971
H	1.17777	0.09859	2.19169
H	2.55800	-0.91986	3.70699
H	4.78455	-1.73048	2.80115
H	3.66724	-3.06058	3.04421

H	3.95689	-2.44726	1.40491
H	1.49503	-2.60140	0.19827
H	4.23441	-1.18084	-0.69300
H	3.63095	3.58984	-0.36594
H	4.67046	2.26614	-0.92958
H	3.21996	2.70824	-1.84362

Tatiomicin **(3)** Conformer 2

C	-4.53525	0.41307	0.08560
C	-4.34092	-0.92762	-0.23751
C	-3.05704	-1.46832	-0.30729
C	-1.95254	-0.64557	-0.05266
C	-2.13998	0.71215	0.24992
C	-3.44827	1.24447	0.33060
C	-0.57041	-1.24505	-0.04092
C	0.56570	-0.22936	-0.26112
C	0.27098	1.17411	0.17285
C	-0.99428	1.57721	0.40801
C	1.94274	-0.69748	0.30724
C	2.84041	0.16963	-0.61558
C	2.79047	1.64717	-0.17799
C	1.39309	2.13954	0.21507
O	-3.54696	2.55936	0.63499
O	-2.79211	-2.76246	-0.61299
C	-4.83970	3.14586	0.69552
C	-3.87900	-3.61843	-0.93226
O	-0.35467	-2.00772	1.15023
O	1.23847	3.30032	0.55316
C	2.11149	-0.45538	1.78412
C	3.00304	-0.99978	2.61959
C	4.08718	-1.99574	2.36486
O	2.20602	-2.03223	-0.08352
O	4.17769	-0.21947	-0.65940
C	3.45069	2.60190	-1.17549
O	0.80092	-0.19220	-1.71248
C	2.11560	-0.02645	-1.95554
O	2.58004	-0.04281	-3.06056
H	-5.54703	0.79432	0.13334
H	-5.20951	-1.54430	-0.43056
H	-0.49037	-1.97290	-0.84966
H	-1.16600	2.61402	0.67485
H	3.36909	1.67373	0.75520
H	-4.67832	4.19369	0.94278
H	-5.35146	3.07463	-0.26958
H	-5.45085	2.67883	1.47464
H	-4.56217	-3.72742	-0.08345
H	-4.42969	-3.25395	-1.80580
H	-3.43654	-4.58605	-1.16343

H	-0.60966	-1.48200	1.91622
H	1.42574	0.27037	2.21443
H	2.93438	-0.66570	3.65459
H	5.05707	-1.55903	2.62839
H	3.95644	-2.86830	3.01548
H	4.12634	-2.33306	1.33200
H	1.64673	-2.59591	0.46822
H	4.20634	-1.15619	-0.88898
H	3.47704	3.60647	-0.75209
H	4.47119	2.27854	-1.38408
H	2.90203	2.64230	-2.11997

Tatiomicin **(3)** Conformer 3

C	-4.53756	0.41213	0.03378
C	-4.33978	-0.93823	-0.24471
C	-3.05652	-1.48512	-0.26471
C	-1.95722	-0.65883	-0.00964
C	-2.14684	0.70468	0.25730
C	-3.45387	1.24531	0.28623
C	-0.57919	-1.26100	0.06550
C	0.54846	-0.24947	-0.20412
C	0.26695	1.15516	0.23377
C	-1.00207	1.56397	0.44192
C	1.94234	-0.71213	0.31125
C	2.79517	0.15641	-0.64463
C	2.77107	1.63193	-0.18704
C	1.38974	2.11516	0.27891
O	-3.55440	2.56882	0.55204
O	-2.79004	-2.79155	-0.51911
C	-4.84714	3.15638	0.58611
C	-3.87559	-3.65606	-0.81583
O	-0.45009	-1.83447	1.36095
O	1.25317	3.26584	0.65741
C	2.14221	-0.47869	1.79232
C	3.12833	-0.94179	2.56588
C	4.31849	-1.77382	2.20846
O	2.07858	-2.07828	-0.05640
O	4.10807	-0.32418	-0.77804
C	3.37244	2.60095	-1.20674
O	0.72850	-0.21691	-1.67283
C	2.02343	-0.04770	-1.94503
O	2.49633	-0.07951	-3.05075
H	-5.54875	0.79780	0.04422
H	-5.20599	-1.55759	-0.43996
H	-0.48650	-2.03772	-0.69872
H	-1.17433	2.60241	0.70212
H	3.39652	1.64955	0.71491
H	-4.68871	4.20839	0.81736

H	-5.34720	3.06889	-0.38392
H	-5.46823	2.70282	1.36533
H	-4.57225	-3.72346	0.02646
H	-4.41186	-3.33005	-1.71352
H	-3.43512	-4.63545	-0.99632
H	0.30395	-2.43720	1.33153
H	1.36794	0.10728	2.27827
H	3.05274	-0.69012	3.62281
H	4.18334	-2.80536	2.55513
H	4.53959	-1.78491	1.14149
H	5.20601	-1.38912	2.72055
H	3.01926	-2.25759	-0.17713
H	4.31217	-0.34907	-1.72395
H	3.40998	3.60244	-0.77675
H	4.38920	2.29851	-1.46516
H	2.78039	2.64604	-2.12482

Tatiomicin **(3)** Conformer 4

C	4.52756	0.40529	0.02791
C	4.32780	-0.95045	0.28092
C	3.04575	-1.49702	0.25342
C	1.95000	-0.66839	-0.00143
C	2.14383	0.69287	-0.27275
C	3.44962	1.23622	-0.25885
C	0.57165	-1.27412	-0.11758
C	-0.54740	-0.26825	0.15119
C	-0.27273	1.11434	-0.36793
C	0.99988	1.53272	-0.53557
C	-1.97480	-0.75119	-0.24508
C	-2.75256	0.21872	0.68473
C	-2.74960	1.64815	0.10533
C	-1.39426	2.07007	-0.47516
O	3.55483	2.55952	-0.52063
O	2.76841	-2.81830	0.43312
C	4.84654	3.15093	-0.51667
C	3.83864	-3.69483	0.75516
O	0.38583	-1.78720	-1.43466
O	-1.26982	3.17361	-0.97781
C	-2.25593	-0.62712	-1.72486
C	-3.29886	-1.10990	-2.40494
C	-4.48683	-1.88067	-1.92467
O	-2.14065	-2.06524	0.25467
O	-4.05945	-0.23195	0.94282
C	-3.27672	2.70791	1.07526
O	-0.63650	-0.15019	1.62253
C	-1.90583	0.09711	1.95092
O	-2.31030	0.17485	3.08154
H	5.53670	0.79535	0.05578

H	5.18991	-1.57071	0.49185
H	0.45287	-2.07378	0.61737
H	1.17206	2.56299	-0.82656
H	-3.42913	1.59882	-0.75498
H	4.69199	4.20213	-0.75401
H	5.31705	3.06625	0.46823
H	5.49204	2.69795	-1.27609
H	4.57977	-3.73005	-0.05023
H	4.32357	-3.40028	1.69153
H	3.39103	-4.68005	0.87602
H	0.94544	-2.56662	-1.51473
H	-1.49489	-0.10555	-2.29732
H	-3.27936	-0.93478	-3.47997
H	-4.41009	-2.92997	-2.23385
H	-4.62514	-1.84333	-0.84529
H	-5.39734	-1.48627	-2.38748
H	-3.05984	-2.14158	0.54153
H	-4.21135	-0.14970	1.89532
H	-3.33053	3.67089	0.56619
H	-4.27895	2.44406	1.41888
H	-2.62909	2.81919	1.94912

Tatiomicin **(3)** Conformer 5

C	-4.54970	0.42332	0.08559
C	-4.35237	-0.92460	-0.20927
C	-3.06457	-1.44192	-0.31190
C	-1.96105	-0.60129	-0.13924
C	-2.15141	0.74770	0.17963
C	-3.46345	1.26687	0.29074
C	-0.58279	-1.20398	-0.24709
C	0.57674	-0.19321	-0.31595
C	0.26455	1.18353	0.18952
C	-1.00414	1.59620	0.38458
C	1.92829	-0.72675	0.27010
C	2.87321	0.19957	-0.54735
C	2.79328	1.65672	-0.02217
C	1.38523	2.13395	0.34681
O	-3.56499	2.58148	0.59086
O	-2.77358	-2.75494	-0.54565
C	-4.86086	3.15109	0.71535
C	-3.84538	-3.64937	-0.82037
O	-0.34700	-2.09810	0.84990
O	1.22560	3.27025	0.75854
C	2.05434	-0.58755	1.76583
C	2.91959	-1.21139	2.57332
C	3.98120	-2.21507	2.25131
O	2.16396	-2.03683	-0.19716
O	4.19657	-0.23522	-0.63921

C	3.48454	2.66971	-0.93883
O	0.88894	-0.05274	-1.74827
C	2.21811	0.10113	-1.92362
O	2.72454	0.16906	-3.00625
H	-5.56300	0.79643	0.15699
H	-5.21821	-1.55932	-0.34975
H	-0.53021	-1.77311	-1.18127
H	-1.17002	2.62295	0.69098
H	3.32962	1.64235	0.93802
H	-4.70111	4.20007	0.95866
H	-5.41701	3.07674	-0.22461
H	-5.42760	2.67306	1.52083
H	-3.38453	-4.61679	-1.01246
H	-4.52097	-3.73236	0.03678
H	-4.40415	-3.33067	-1.70558
H	-1.03323	-2.77588	0.79224
H	1.36144	0.10794	2.23219
H	2.83706	-0.96210	3.63086
H	4.06016	-2.42479	1.18638
H	4.95108	-1.86607	2.62439
H	3.77746	-3.15613	2.77602
H	1.49057	-2.58133	0.23885
H	4.61833	-0.10803	0.21786
H	3.50869	3.64414	-0.45022
H	4.50705	2.35462	-1.15167
H	2.95610	2.77665	-1.88947

Tatiomicin **(3)** Conformer 6

C	-4.28479	0.73389	-0.34247
C	-4.21858	-0.65752	-0.39273
C	-2.98917	-1.30528	-0.33874
C	-1.80953	-0.56016	-0.23805
C	-1.86922	0.83744	-0.17540
C	-3.12428	1.49079	-0.23025
C	-0.49683	-1.30811	-0.21224
C	0.77000	-0.42715	-0.25525
C	0.57260	1.04220	-0.02772
C	-0.65285	1.60088	-0.04097
C	1.98783	-0.94272	0.56208
C	3.10935	-0.27037	-0.27814
C	3.15808	1.24398	-0.00550
C	1.77677	1.89684	0.09971
O	-3.09914	2.84145	-0.17036
O	-2.82976	-2.66252	-0.36235
C	-4.33260	3.54565	-0.21628
C	-3.98236	-3.48086	-0.52990
O	-0.42209	-2.18701	0.91696
O	1.69548	3.09852	0.28654

C	2.16703	-0.63733	2.02624
C	1.38079	-0.21249	3.02080
C	-0.06372	0.15444	3.07713
O	2.19195	-2.33419	0.33631
O	4.37920	-0.79795	-0.04574
C	4.06129	2.01289	-0.97298
O	1.24573	-0.55676	-1.65331
C	2.59025	-0.54583	-1.69212
O	3.21306	-0.72360	-2.70138
H	-5.25532	1.21026	-0.38932
H	-5.13965	-1.22080	-0.47416
H	-0.44907	-1.91494	-1.12353
H	-0.72884	2.67720	0.06309
H	3.58501	1.33241	1.00190
H	-4.85934	3.35889	-1.15757
H	-4.97426	3.27454	0.62830
H	-4.07371	4.60093	-0.15018
H	-4.67818	-3.35735	0.30563
H	-4.48761	-3.25495	-1.47356
H	-3.61894	-4.50679	-0.55039
H	-1.17145	-2.79258	0.82757
H	3.20097	-0.84597	2.29994
H	1.89227	-0.11174	3.97834
H	-0.52063	-0.31238	3.95638
H	-0.17413	1.23768	3.20731
H	-0.63234	-0.15297	2.20533
H	1.41442	-2.77591	0.71369
H	4.31182	-1.75889	-0.11854
H	4.12785	3.05438	-0.65670
H	5.06234	1.57967	-0.97598
H	3.67295	1.98874	-1.99438

Tatiomicin (**3**) C-2 epimer conformation 1

C	4.56123	0.44320	-0.18749
C	4.37620	-0.92766	0.00215
C	3.09363	-1.45190	0.14567
C	1.98154	-0.60058	0.11438
C	2.15850	0.77438	-0.09270
C	3.46658	1.30139	-0.24322
C	0.60639	-1.21118	0.28215
C	-0.55962	-0.20650	0.44230
C	-0.25738	1.22116	0.07125
C	1.00494	1.64427	-0.14423
C	-1.92350	-0.65938	-0.19192
C	-2.86526	0.19368	0.71422
C	-2.79622	1.71629	0.40643

C	-1.38119	2.19563	0.03618
O	3.55662	2.64406	-0.43016
O	2.81332	-2.78735	0.29558
C	4.84947	3.23354	-0.58076
C	3.89862	-3.71082	0.42516
O	0.30982	-2.09425	-0.81977
O	-1.20580	3.36729	-0.25804
C	-2.00114	-0.42483	-1.68282
C	-2.83744	-0.97425	-2.56999
C	-3.95594	-1.95411	-2.37610
O	-2.22066	-2.00127	0.18505
O	-4.20227	-0.21821	0.70661
O	-0.86685	-0.20295	1.89573
C	-2.19276	-0.03553	2.08411
O	-2.70450	-0.05120	3.17020
C	-3.82873	2.19549	-0.63043
H	5.56818	0.82127	-0.29423
H	5.24498	-1.57085	0.03036
H	0.60962	-1.80140	1.20291
H	1.16585	2.69360	-0.35663
H	-3.02881	2.23699	1.34398
H	4.67271	4.29881	-0.71206
H	5.46129	3.07122	0.31123
H	5.36286	2.83646	-1.46115
H	3.44005	-4.68672	0.56931
H	4.51260	-3.72547	-0.47964
H	4.51692	-3.46540	1.29273
H	1.00227	-2.77044	-0.81246
H	-1.27062	0.27638	-2.07320
H	-2.69508	-0.65756	-3.60195
H	-3.80596	-2.82849	-3.01949
H	-4.05505	-2.29164	-1.34817
H	-4.90285	-1.50008	-2.68943
H	-1.56272	-2.55653	-0.26299
H	-4.21919	-1.16756	0.88743
H	-3.72428	3.27376	-0.75234
H	-4.83695	1.97439	-0.28363
H	-3.68088	1.71637	-1.59804

Tatiomicin* (**3**) C-2 epimer conformation 2

C	4.56019	0.43112	-0.14618
C	4.37434	-0.93590	0.06132
C	3.09023	-1.48068	0.13414
C	1.97669	-0.63773	-0.00134
C	2.15643	0.74461	-0.18524
C	3.46522	1.28201	-0.26867
C	0.58912	-1.23624	-0.00892
C	-0.54104	-0.23853	0.33503

C	-0.25341	1.20343	0.02391
C	1.00587	1.62354	-0.21874
C	-1.95046	-0.64536	-0.22126
C	-2.80948	0.13922	0.81762
C	-2.74688	1.68230	0.60191
C	-1.37468	2.17611	0.10062
O	3.55694	2.62595	-0.45342
O	2.83169	-2.80304	0.33380
C	4.85191	3.22704	-0.50662
C	3.92833	-3.69829	0.51788
O	0.32511	-1.90660	-1.25473
O	-1.22925	3.35285	-0.18870
C	-2.16267	-0.29464	-1.67591
C	-3.06170	-0.79282	-2.53258
C	-4.13982	-1.81516	-2.33017
O	-2.21256	-2.01529	0.06972
O	-4.14687	-0.26360	0.88888
O	-0.72491	-0.33711	1.80245
C	-2.03026	-0.18568	2.10995
O	-2.45383	-0.28093	3.22925
C	-3.88754	2.23742	-0.26916
H	5.56932	0.81493	-0.20060
H	5.24678	-1.56652	0.16257
H	0.53713	-2.02715	0.73791
H	1.17496	2.67828	-0.39545
H	-2.85648	2.14259	1.59231
H	4.67583	4.29166	-0.64451
H	5.39937	3.06454	0.42627
H	5.43174	2.84062	-1.34981
H	3.48393	-4.67934	0.67223
H	4.56968	-3.72533	-0.36821
H	4.52064	-3.42451	1.39621
H	0.57469	-1.33341	-1.98948
H	-1.49349	0.46624	-2.06561
H	-3.01745	-0.38623	-3.54160
H	-4.16474	-2.21997	-1.32220
H	-5.11494	-1.36751	-2.55188
H	-4.01262	-2.64211	-3.03798
H	-1.68034	-2.54156	-0.54395
H	-4.16127	-1.22233	1.01024
H	-3.77588	3.31938	-0.34077
H	-4.85062	2.00973	0.18486
H	-3.86903	1.81546	-1.27397

Tatiomicin* (**3**) C-2 epimer conformation 3

C	4.56887	0.42424	-0.08421
C	4.37547	-0.94760	0.08135
C	3.08932	-1.49272	0.09922

C	1.98232	-0.64566	-0.04361
C	2.16813	0.73845	-0.19581
C	3.47833	1.27799	-0.22267
C	0.59532	-1.23944	-0.11861
C	-0.52281	-0.24745	0.27131
C	-0.24632	1.19363	-0.05097
C	1.01844	1.61476	-0.26599
C	-1.94771	-0.65701	-0.22541
C	-2.75785	0.13160	0.84054
C	-2.71428	1.67576	0.59689
C	-1.36899	2.16074	0.01333
O	3.57531	2.62649	-0.37228
O	2.82552	-2.82220	0.24923
C	4.87210	3.22428	-0.39935
C	3.91804	-3.72381	0.42015
O	0.41887	-1.71022	-1.45886
O	-1.24694	3.32549	-0.33046
C	-2.19686	-0.32318	-1.68460
C	-3.18551	-0.76421	-2.46827
C	-4.35276	-1.65014	-2.14329
O	-2.07837	-2.05621	0.04197
O	-4.06728	-0.37073	0.98284
O	-0.64958	-0.34408	1.75531
C	-1.93376	-0.18998	2.09259
O	-2.36270	-0.29073	3.21390
C	-3.90705	2.20765	-0.21332
H	5.57938	0.80799	-0.09691
H	5.24446	-1.58170	0.19092
H	0.52488	-2.07625	0.57829
H	1.18965	2.66919	-0.44287
H	-2.76295	2.15383	1.58469
H	4.70111	4.29121	-0.52585
H	5.40650	3.04854	0.53886
H	5.46282	2.84705	-1.23919
H	3.47165	-4.71027	0.52836
H	4.57687	-3.71723	-0.45348
H	4.49358	-3.48474	1.31971
H	-0.32832	-2.32378	-1.45475
H	-1.45196	0.31633	-2.14441
H	-3.14226	-0.43952	-3.50587
H	-4.19656	-2.65691	-2.54777
H	-4.55788	-1.72803	-1.07662
H	-5.25591	-1.25968	-2.62185
H	-3.01116	-2.24222	0.21186
H	-4.26226	-0.42185	1.93095
H	-3.80855	3.28840	-0.31547
H	-4.84136	1.98565	0.30098
H	-3.94495	1.76425	-1.20831

Tatiomicin* (**3**) C-2 epimer conformation 4

C	4.56905	0.41177	-0.02383
C	4.37016	-0.96263	0.12003
C	3.08216	-1.49967	0.10119
C	1.97985	-0.64793	-0.03406
C	2.17200	0.73299	-0.19667
C	3.48384	1.26839	-0.19130
C	0.59066	-1.24234	-0.14221
C	-0.52197	-0.25225	0.22807
C	-0.24876	1.17299	-0.17677
C	1.02172	1.59807	-0.34823
C	-1.97042	-0.68939	-0.17106
C	-2.72074	0.20239	0.86438
C	-2.68802	1.71971	0.49635
C	-1.37183	2.14091	-0.18730
O	3.58716	2.61550	-0.34349
O	2.80091	-2.83723	0.17472
C	4.88601	3.20982	-0.35878
C	3.87707	-3.75380	0.37938
O	0.36252	-1.67628	-1.49028
O	-1.26262	3.26136	-0.65858
C	-2.28037	-0.46516	-1.63845
C	-3.31362	-0.93798	-2.34021
C	-4.47846	-1.77583	-1.89916
O	-2.13094	-2.04751	0.22873
O	-4.02493	-0.27617	1.11218
O	-0.57885	-0.26108	1.71891
C	-1.84091	-0.03724	2.09952
O	-2.21597	-0.03410	3.24464
C	-3.91825	2.19555	-0.29144
H	5.58007	0.79395	-0.00713
H	5.23472	-1.60158	0.23785
H	0.49334	-2.08838	0.53892
H	1.19356	2.64598	-0.55986
H	-2.68210	2.27645	1.44378
H	4.71947	4.27585	-0.49802
H	5.40700	3.04219	0.58833
H	5.48681	2.82260	-1.18682
H	4.40243	-3.54121	1.31500
H	3.41914	-4.73913	0.43654
H	4.58280	-3.72771	-0.45628
H	0.90994	-2.45761	-1.63197
H	-1.54353	0.11634	-2.18120
H	-3.31349	-0.69221	-3.40037
H	-4.62593	-1.78518	-0.82076
H	-5.39941	-1.39880	-2.35463
H	-4.35896	-2.80916	-2.24508

H	-3.04448	-2.15262	0.52694
H	-4.18086	-0.22839	2.06765
H	-3.81315	3.26101	-0.49658
H	-4.82501	2.03660	0.29094
H	-4.01568	1.66487	-1.23841

Tatiomicin* (**3**) C-2 epimer conformation 5

C	4.57308	0.43055	-0.14859
C	4.37961	-0.93988	0.03601
C	3.09195	-1.46155	0.14350
C	1.98465	-0.60595	0.08780
C	2.17004	0.76808	-0.11817
C	3.48254	1.29180	-0.23541
C	0.60313	-1.21230	0.20180
C	-0.55205	-0.20347	0.39582
C	-0.24667	1.21685	0.00582
C	1.01840	1.63756	-0.20194
C	-1.93877	-0.67044	-0.18562
C	-2.84567	0.18843	0.75490
C	-2.75669	1.72307	0.43622
C	-1.36751	2.18679	-0.03917
O	3.58067	2.63396	-0.42251
O	2.80213	-2.79607	0.27281
C	4.87821	3.21923	-0.54702
C	3.87919	-3.72348	0.43589
O	0.31754	-2.02231	-0.95821
O	-1.21235	3.34074	-0.40660
C	-2.10201	-0.42220	-1.66943
C	-2.98121	-0.99074	-2.50253
C	-4.05195	-2.00702	-2.22941
O	-2.17582	-2.02105	0.18084
O	-4.16747	-0.25839	0.87998
O	-0.81284	-0.19393	1.85683
C	-2.13423	-0.03927	2.09552
O	-2.60073	-0.04189	3.20015
C	-3.85750	2.23299	-0.51114
H	5.58361	0.80589	-0.22794
H	5.24574	-1.58530	0.08700
H	0.58284	-1.85536	1.08541
H	1.18277	2.68415	-0.42522
H	-2.90357	2.24462	1.39076
H	4.70744	4.28444	-0.68661
H	5.46991	3.05913	0.35883
H	5.40969	2.81710	-1.41431
H	3.41283	-4.69834	0.56097
H	4.52377	-3.73732	-0.44751
H	4.46876	-3.48344	1.32483
H	0.99967	-2.70793	-0.98162

H	-1.41517	0.29978	-2.09942
H	-2.91673	-0.67268	-3.54168
H	-3.86934	-2.91308	-2.81873
H	-4.11566	-2.28617	-1.18023
H	-5.02330	-1.62148	-2.55978
H	-1.52785	-2.53978	-0.32130
H	-4.64274	-0.06917	0.06296
H	-3.70007	3.29685	-0.68697
H	-4.84360	2.11322	-0.05968
H	-3.83726	1.72200	-1.47527

Tatiomicin* (**3**) C-2 epimer conformation 6

C	4.61238	0.34610	0.03780
C	4.38540	-0.99469	0.33203
C	3.08930	-1.51048	0.37970
C	2.00030	-0.66993	0.15066
C	2.21145	0.69415	-0.12868
C	3.53268	1.20048	-0.19532
C	0.60475	-1.25041	0.12897
C	-0.51158	-0.20478	0.35848
C	-0.19750	1.19437	-0.09085
C	1.07988	1.57635	-0.30584
C	-1.91960	-0.64111	-0.16384
C	-2.76505	0.25569	0.78201
C	-2.68367	1.76880	0.39560
C	-1.30523	2.17775	-0.16737
O	3.65791	2.52227	-0.47548
O	2.86842	-2.82737	0.71441
C	4.96589	3.09429	-0.55320
C	3.25354	-3.79286	-0.27726
O	0.45498	-1.90692	-1.13389
O	-1.14778	3.30264	-0.61431
C	-2.09268	-0.44729	-1.65930
C	-3.05911	-0.93774	-2.44124
C	-4.26745	-1.75351	-2.08491
O	-2.08778	-2.00711	0.22845
O	-4.08716	-0.21625	0.90906
O	-0.70885	-0.15156	1.83719
C	-2.00506	0.04737	2.09711
O	-2.48675	0.06036	3.20109
C	-3.82846	2.23946	-0.51557
H	5.62803	0.71486	0.00827
H	5.22525	-1.64885	0.53520
H	0.51252	-1.98087	0.93479
H	1.27146	2.60652	-0.57889
H	-2.76802	2.33954	1.33042
H	4.81190	4.14490	-0.78930
H	5.49021	3.00696	0.40243

H	5.55441	2.62358	-1.34570
H	2.67892	-3.64065	-1.19475
H	4.32421	-3.73344	-0.49411
H	3.02545	-4.77046	0.14535
H	-0.32455	-2.47646	-1.07049
H	-1.30759	0.12097	-2.14527
H	-2.95949	-0.72125	-3.50291
H	-4.51709	-1.72418	-1.02536
H	-5.13739	-1.38315	-2.63565
H	-4.12941	-2.79797	-2.38772
H	-3.03080	-2.16552	0.36725
H	-4.32940	-0.16661	1.84636
H	-3.70466	3.30446	-0.71215
H	-4.78917	2.08030	-0.02730
H	-3.82982	1.70691	-1.46661

Tatiomicin* (**3**) C-2 epimer conformation 7

C	-4.32071	0.70317	-0.26829
C	-4.25169	-0.68964	-0.19867
C	-3.01684	-1.33129	-0.14638
C	-1.83410	-0.58005	-0.16881
C	-1.89580	0.82030	-0.22697
C	-3.15727	1.46713	-0.27915
C	-0.51312	-1.32285	-0.14087
C	0.74886	-0.44432	-0.32797
C	0.55508	1.04307	-0.21904
C	-0.67456	1.59526	-0.21739
C	2.02447	-0.88454	0.45677
C	3.09256	-0.28368	-0.51108
C	3.14141	1.27299	-0.46267
C	1.76263	1.91156	-0.20961
O	-3.13432	2.82416	-0.34099
O	-2.85463	-2.69179	-0.05398
C	-4.37350	3.53393	-0.38611
C	-4.01351	-3.53020	-0.10284
O	-0.37395	-2.09777	1.06652
O	1.68143	3.11842	-0.04685
C	2.27913	-0.48013	1.89031
C	1.55174	0.02595	2.89293
C	0.11634	0.43786	3.00096
O	2.22567	-2.29887	0.32986
O	4.37936	-0.80097	-0.32411
O	1.14496	-0.69982	-1.74681
C	2.48684	-0.68530	-1.86699
O	3.05480	-0.92893	-2.89696
C	4.20667	1.84644	0.48762
H	-5.29324	1.17283	-0.31082
H	-5.17257	-1.25646	-0.18571

H	-0.50609	-2.01362	-0.98871
H	-0.75463	2.67461	-0.19802
H	3.41330	1.60967	-1.47169
H	-4.10648	4.58767	-0.42693
H	-4.94723	3.26754	-1.27846
H	-4.96985	3.34210	0.51060
H	-3.64177	-4.55114	-0.04963
H	-4.67406	-3.33599	0.74654
H	-4.55739	-3.38684	-1.04009
H	-1.11984	-2.71545	1.07269
H	3.31780	-0.69656	2.12926
H	2.11369	0.17840	3.81336
H	-0.28589	0.09367	3.95909
H	0.03444	1.53120	3.00178
H	-0.51643	0.04122	2.21501
H	1.48435	-2.71703	0.79700
H	4.30617	-1.76541	-0.30430
H	4.19409	2.93344	0.40775
H	5.19405	1.48217	0.20838
H	4.01344	1.57431	1.52509

Genome sequencing and analysis of biosynthetic pathway

Bioinformatic analysis

Artemis (Rutherford, Parkhill et al. 2000) was used for visual inspection and comparative genomics. The web based software RAST [10] was used for annotation of the sequence, while genome mining was performed using the automated software antiSMASH 6.0.1.[11] The ends of the tatiomicin gene cluster identified due to its homology to the rishirilide gene cluster were manually defined.[12]

Table S9 Size of contigs from the assembly of the PacBio dataset.

	Size in Kb		Size in Kb
Contig1	42	Contig8	302
Contig2	16	Contig9	543
Contig3	328	Contig10	134
Contig4	995	Contig11	123
Contig5	4418	Contig12	615
Contig6	1767	Contig13	377
Contig7	12	Total	9672

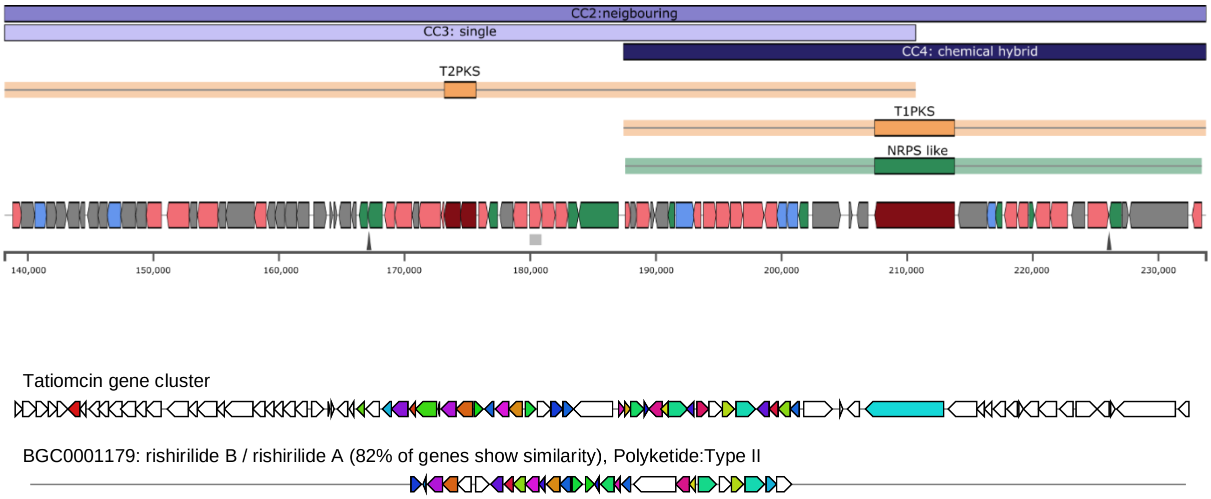

Figure S32 antiSMASH analysis of the contig containing the tatiomicin gene cluster. (**Top**) Region 2 depicting a putative T1PKS & T2PKS biosynthetic gene cluster. Genes colours: red – core biosynthetic genes, pink – additional biosynthetic genes, blue – transport related genes, green – regulatory genes, grey – other genes (**Bottom**) comparison to rishirilide gene cluster as depicted by antiSMASH

Table S10. Deduced function of ORFs in the tatiomicin gene cluster from *Amycolatopsis* DEM30355

ORF	AA	Most similar Protein	E value	ident	accession
tatO10	261	3-oxoacyl-ACP reductase FabG [*Micromonospora* sp. KC213]	2E-145	79.69%	WP_132336555.1
tatK1	76	act minimal PKS acyl carrier protein [*Streptomyces* sp. SolWspMP-5a-2]	8E-19	57.00%	SCD95577.1
tatK2	423	ketosynthase chain-length factor [*Amycolatopsis balhimycina*]	0	73.00%	WP_020639073.1
tatK3	418	beta-ACP synthase [*Streptacidiphilus jiangxiensis*]	0	80.00%	WP_042441868.1
tatO3	245	3-oxoacyl-ACP reductase [*Actinosynnema* sp. ALI-1.44]	2E-119	73.00%	WP_076992001.1
tatR2	242	SARP family pathway specific regulatory protein [*Streptomyces collinus*]	1E-72	51.00%	WP_020939953.1
tatO1	351	luciferase [*Actinosynnema* sp. ALI-1.44]	0	77.00%	WP_076991986.1
tatP	372	putative phosphotransferase [*Streptomyces bottropensis*]	4E-112	53.00%	AHL46718.1
TatC1	312	MBL fold metallo-hydrolase [*Actinosynnema* sp. ALI-1.44]	7E-132	61.00%	WP_076992077.1
tatM1	349	methyltransferase [*Acidobacteriales* bacterium 13_1_40CM_3_55_5]	3E-112	50.00%	OLD18459.1

TatC2	320	cyclase [Actinobacteria bacterium 13_2_20CM_2_71_6]	5E-124	56.00%	OLB79503.1
tatR1	269	DNA-binding transcriptional activator of the SARP family [*Streptomyces* sp. MnatMP-M17]	1E-67	48.00%	SCF79391.1
tatR3	1052	SARP family transcriptional regulator [*Streptomyces scabiei*]	0	42.00%	WP_037700027.1
tatC3	144	hydroxylacyl-CoA dehydrogenase [*Actinosynnema sp*. ALI-1.44]	3E-69	75.00%	WP_076991988.1
tatC4	160	hydroxylacyl-CoA dehydrogenase [*Streptomyces acidiscabies*]	4E-28	42.00%	WP_050987403.1
tatO5	357	alkene reductase [*Actinosynnema sp*. ALI-1.44]	4E-146	64.00%	WP_076992078.1
tatO4	100	antibiotic biosynthesis monooxygenase [*Actinosynnema* sp. ALI-1.44]	6E-42	67.00%	WP_076991989.1
tatO6	340	luciferase family oxidoreductase [*Streptomyces scabiei*]	3E-112	58.00%	WP_037700025.1
tatR4	170	MarR family transcriptional regulator [*Amycolatopsis sp*. MJM2582]	1E-49	58.00%	WP_037347867.1
tatT4	495	MFS transporter [*Actinosynnema sp*. ALI-1.44]	0	71.00%	WP_076992079.1
tatO2	205	flavin reductase family protein [*Kitasatospora mediocidica*]	2E-61	57.93%	WP_035798953.1

tatM2	338	O-methyltransferase [*Nonomuraea jiangxiensis*]	2E-108	53.00%	SDI34086.1
tatC5	375	two-component flavin-dependent monooxygenase [*Micromonospora marina*]	3E-116	52.00%	SCF37294.1
tatO8	325	oxidoreductase [*Amycolatopsis sp.* MJM2582]	3E-138	63.00%	WP_037347708.1
tatO9	548	Putative polyketide hydroxylase [*Streptomyces bottropensis* ATCC 25435]	0	56.00%	EMF53299.1
tatT1	331	amino acid ABC transporter substrate-binding protein, PAAT family [*Actinoplanes derwentensis*]	3E-97	52.00%	SDT78871.1
tatT2	255	ABC transporter related protein [*Streptomyces thermoautotrophicus*]	6E-127	73.00%	KWX02137.1
tatT3	299	ABC transporter permease [*Streptomyces thermoautotrophicus*]	2E-118	67.00%	WP_066891941.1
tatR5	255	SARP family transcriptional regulator [*Streptomyces sp.* PTY087I2]	5E-62	50.00%	WP_065488167.1
tatS1	2120	type I polyketide synthase [*Amycolatopsis vastitatis*]	0.0	89.38%	WP_093947784.1
TatO11	393	cytochrome P450 [*Amycolatopsis rifamycinica*]	0.0	94.91%	WP_051735947.1

Table S11. Homologous genes between the tatiomicin and rishirilide biosynthetic gene clusters

tatiomicin ORFs	rishirilide ORFs	qurery cover	E value	ident
tatO10	rslO10	98%	1.00E-127	70.04%
tatK1	rslK1	94%	1E-18	47%
tatK2	rslK2	93%	2E-162	63%
tatK3	rslK3	99%	0	68%
tatO3	rslO3	99%	1E-72	49%
tatR2	rslR2	95%	4E-67	47%
tatO1	rslO1	99%	8E-174	68%
tatP	rslP	97%	7E-117	53%
TatC2	rslC2	92%	4E-96	50%
tatM1	rslO9	10%	0.75	31%
TatC1	rslC1	93%	8E-103	52%
tatR1	rslR1	93%	1E-61	47%
tatR3	rslR3	98%	0	41%
tatC3	rslC3	91%	2E-61	65%
tatC4	rslC3	91%	1E-30	40%
tatO5	rslO5	98%	1E-134	56%
tatO4	rslO4	87%	2E-36	56%
tatO6	rslO6	99%	2E-113	56%
tatR4	rslR4	86%	6E-45	49%
tatT4	rslT4	92%	0	66%
tatO2	rslO2	100%	4E-52	52%
tatM2	rslR4	18%	0.57	26%
tatC5	rslC2	28%	0.64	32%
tatO8	rslO8	100%	6E-131	61%
tatO9	rslO9	97%	0	56%

tatT1	rslT1	82%	2E-99	52%
tatT2	rslT2	96%	4E-102	60%
tatT3	rslT3	88%	3E-111	67%
tatR5	rslR2	91%	5E-60	49%
tatS1	rslK3	14%	1E-25	28.87%
tatO11	No significant hit found			

Proposed biosynthesis of tatiomicin

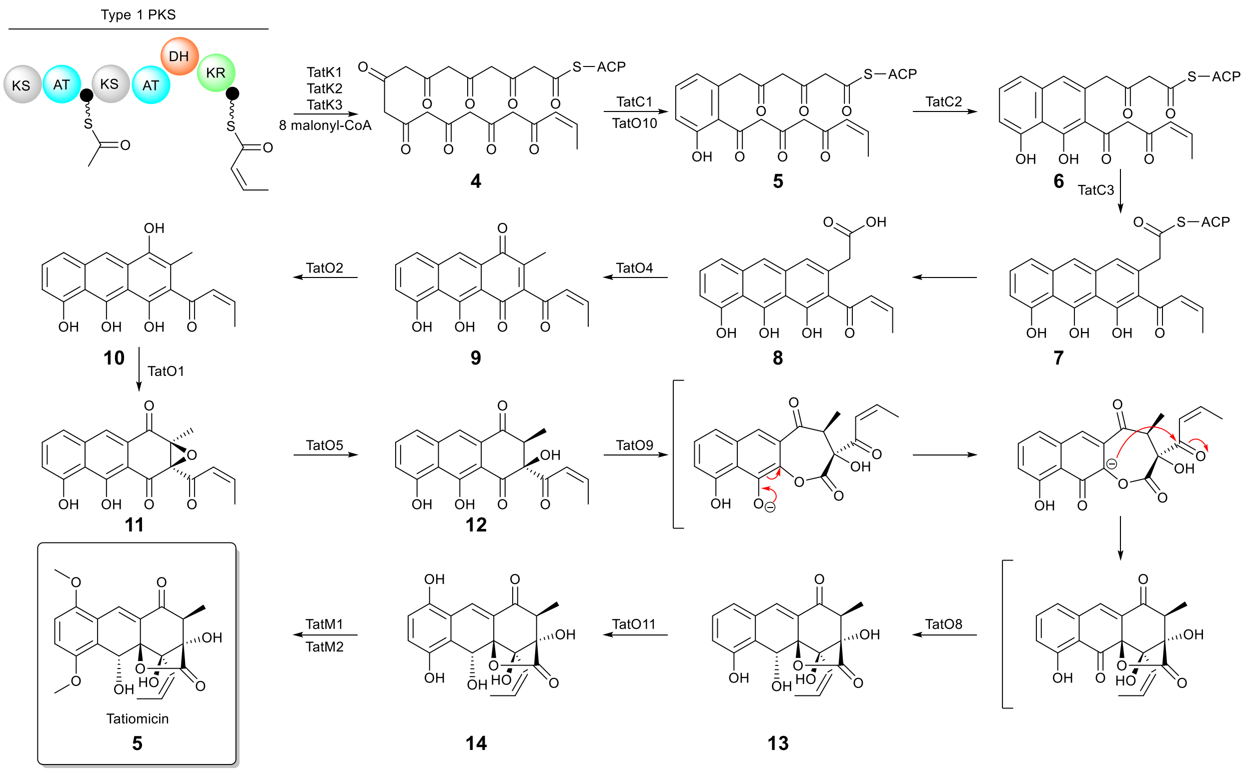

Figure S33. Proposed biosynthetic pathway of tatiomicin via hybrid type I and type II polyketide synthase.

The starting moiety in biosynthetic assembly of tatiomicin and rishirilide are different. In rishirilide biosynthesis, enzymes encoded by *rslK4*, *rslO3* and *rslA* are proposed to be involved in production of 4-methylpentanoyl-ACP.[13] Tatiomicin BGC lacks the homologous of these genes, instead it contains *tatS1* that encodes a modular type I polyketide synthase enzyme with module and domain organization required for the formation of cis-crotonyl moiety. TatS1 consist of a loading module and one extender module which incorporate a malonyl-CoA onto the priming acetate unit followed by ketoreduction and dehydration to produce cis-crotonyl backbone. Cis-crotonyl moiety is then elongated via attachment of eight malonyl-CoA by minimal PKS enzymes TatK1, TatK2, and TatK3 homologous to RslK1, RslK2, RslK3, respectively. [13] Sequence analysis of TatC1, TatC2, and TatC3 showed homology to rishirilide cyclases RslC1, RslC2, and RslC3, respectively.[13, 14] It has been proposed that C9-ketoreductase RslO10 involves in first ring cyclisation of rishirilide.[13] The *tatO10* in *tat* gene cluster shows 70% identity to *rslO10*, therefore, TatC1 and TatO10 together involve in first ring cyclization/aromatization of tatiomicin **5**. TatC2 and TatC3 catalyse the second and third ring cyclization to produce intermediate **7**. Several tailoring enzymes are then modifying this backbone. The function of most of the enzymes proposed here are based on their close homology to the enzymes involved in rishirilide biosynthesis. The *tatO4* with 56% identity to *rslO4* could be responsible for quinone **9** formation. Flavin mononucleotide (FMN)-dependent monooxygenase TatO1 (68% identity to RslO1) together with putative flavin reductase TatO2 (52% identity to RslO2) are proposed to be responsible for epoxy formation in third ring.[14] Flavin

dependent RslO5 reductively ring-opens the epoxide moiety in rishirilide biosynthesis.[15] In *tat* gene cluster, *tatO5* showed homology to *rslO5* (56% identity), suggesting that TetO5 is converting **11** to **12** which then undergoes oxidative rearrangement of carbon backbone to give intermediate **13**. The enzyme candidate for this rearrangement is TatO9. It shares a 56% identity to RslO9, that has been shown to be responsible for the carbon backbone rearrangement via lactone-forming Baeyer-Villiger oxidation followed by intramolecular aldol condensation.[15] The ketoreductase TatO8 (homologous of RslO8) catalyses the ketone reduction in the second ring to form **13**. Compared to rishirilide, three additional tailoring enzymes are involved in the assembly of tatiomicin. The gene *tatO11* located downstream of the *tat* gene cluster encodes for a P_{450} enzyme that could be responsible for hydroxylation of the first ring of tatiomicin at para position of the previous hydroxyl group. Both these hydroxyls are the site for methylation by the two methyl transferases TatM1 and TatM2 to yield the final tatiomicin product.

Heterologous expression of tatiomicin

Table S12. Primers used for the screening of the tatiomicin gene cluster

Name	Sequence
Tat_1_for	GATCGCCACCGCCGACCTCTAC
Tat_1_rv	CTCAGCACCAGGTCCTCCACTTC
Tat_2_for	CACAAAGTGTTGATCGCCAACCGTG
Tat_2_rv	GTCTCCTCGATCAGTTTCTGGTGCC
Tat_3_for	CAGCTGGTTCGCTTCGTCGTAGTGG
Tat_3_rv	ACCACCACCGAATATGACGCAGCCG
Tat_4_for	CCGTTGCTGATCTTCGTGACCACTG
Tat_4_rv	AATCGTCGAAGTAGTCGCTCTTGCC

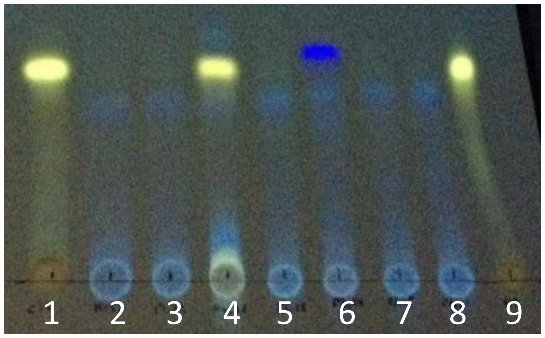

1… Extract containing DEM30355/A
2…M1152
3…M1152 exconjugant Nr. P1_23
4…M1152 exconjugant Nr. P4_22
5…M1152 exconjugant Nr. P2_16
6…M1152 exconjugant Nr. P3_14
7…M1152 exconjugant Nr. P2_15
8…M1152 exconjugant Nr. P3_21
9…Extract containing DEM30355/A

Figure S34. Example TLC of the exconjugant screening for tatiomicin production

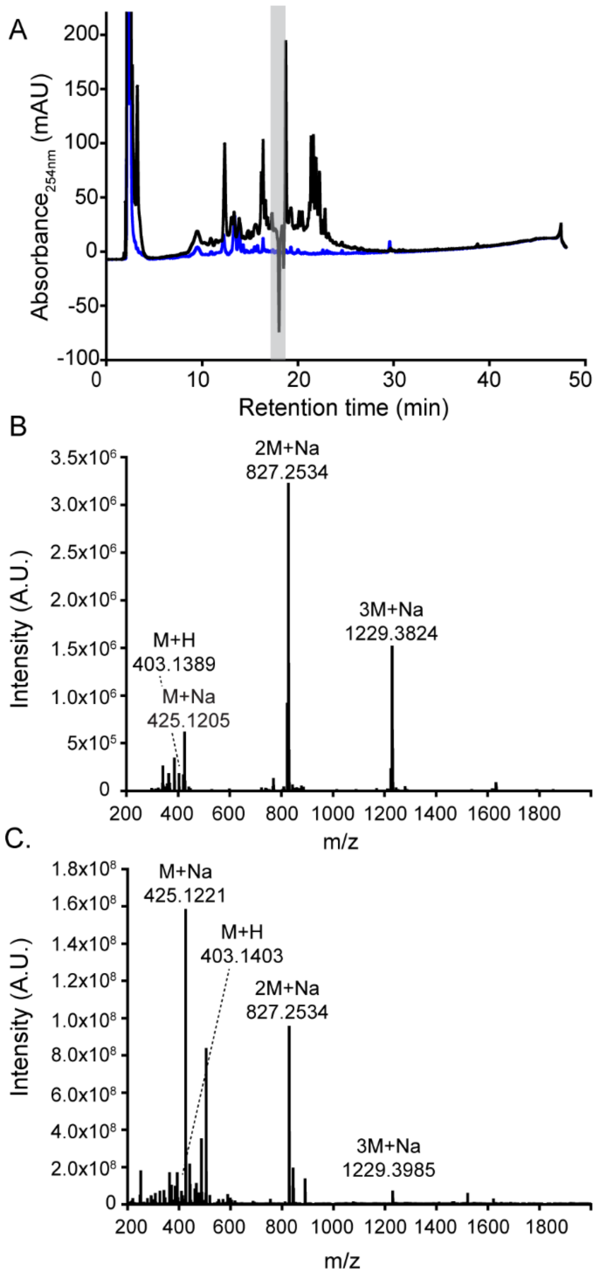

Figure S35 Detection of tatiomicin in the heterologous host *S. coelicolor* M1152 A) extracts of *S. coelicolor* (blue) and *S. coelicolor* M1152::tat (black). In grey is the retention time of purified tatiomicin according to a purified standard. B) MS spectrum of tatiomicin purified from the native host *Amycolatopsis* DEM30355. C) MS spectrum of tatiomicin purified from the heterologous host *S. coelicolor* M1152::tat

Bioactivity Studies

MIC determination against a panel of bacterial isolates

Table S13 MIC assay against a panel of pathogenic microorganisms using agar dilution method. Organism name and code are provided. The MIC was determined after 22 h of growth via visible inspection and given in mg/L.

	Organism	Org. Code	Tatiomicin/Tatiomicin*
Gram-negative	*Acinetobacter baumannii*	ATCC 19606	>64
	Burkholderia cepacia	ATCC 25416	>64
	Enterobacter cloacae	NCTC 11936	>64
	Escherichia coli	NCTC 10418	>64
	Klebsiella pneumoniae	NCTC 9528	>64
	Providencia rettgeri	NCTC 7475	>64
	Pseudomonas aeruginosa	NCTC 10662	>64
	Salmonella typhimurium	NCTC 74	>64
	Serratia marcescens	NCTC 10211	>64
	Yersinia enterocolitica	NCTC 11176	>64
Gram-positive	*Bacillus subtilis*	NCTC 9372	16
	Enterococcus faecalis	NCTC 775	16
	Enterococcus faecium	NCTC 7171	64
	Listeria monocytogenes	NCTC 11994	16
	Staphylococcus epidermidis	NCTC 11047	8
	Staphylococcus aureus	NCTC 6571	8
	Staphylococcus aureus (MRSA)	NCTC 11939	16
	Streptococcus pyogenes	NCTC 8306	8
Yeast	*Candida albicans*	ATCC 90028	>64
	Candida glabrata	NCPF 9725	>64

Table S14 MIC assay against a panel of methicillin resistant *Staphylococcus aureus* (MRSA) using agar dilution method. Organism name and code are provided. The MIC was determined after 22 h of growth via visible inspection and given in mg/L.

MRSA Ref.		Tatiomicin/Tatiomicin*
Bel	97597	4
Bel	97598	4
Fin	37481	4
Fin	54511	4
Fin	54518	8
Fra	462	8
Fra	920	8
Fra	95035	8
Ger	131/98 (Sger 11d2)	8
Ger	1966/97 (Han 111c)	8
Ger	2594-1/97 (Sger 11a)	8
Ger	2594-2/97 (Sger 11b)	8
EMRSA 15	1729/98	8
EMRSA 15	1758/98	8
EMRSA 15	14956	8
EMRSA 15	12484/98	8
EMRSA 15	14185/98	8
EMRSA 15	16822/98	8
EMRSA 15	19972/98	8
EMRSA 15	20460/98	8
EMRSA 15	21268/98	8
EMRSA 15	21698/98	8
EMRSA 15	2501/98	8
EMRSA 15	6323/98	8
EMRSA 16	00036/95	4
EMRSA 16	00998/95	4
EMRSA 16	03732/95	4
EMRSA 16	07121/95	4
EMRSA 16	07924/95	4
EMRSA 16	18200/95	8
EMRSA 16	18205/95	4
EMRSA 16	21354/95	4
EMRSA 16	23728/98	8
EMRSA 16	23729/98	4
EMRSA 16	32010/96	4
EMRSA 16	34256/96	4
MRSA	NCTC 11939	4
Staphylococcus aureus	NCTC 6571	4

Mode of Action Studies

Reporter strain panel

A *B. subtilis* reporter strain panel, gyrA, ypuA, fabHA, ϕ105, helD and liaI,[16-18] was used to indicate likely modes of action of tatiomicin/tatiomicin*. *B. subtilis* reporter strains were analysed in a Kirby Bauer disc diffusion assay using nutrient agar supplemented with X-Gal (100 µg/mL). LacZ activity is visualised by blue colouration due to hydrolysis of X-Gal. The plates were imaged on black background to observe the halo and on white background to observe the blue coloration.

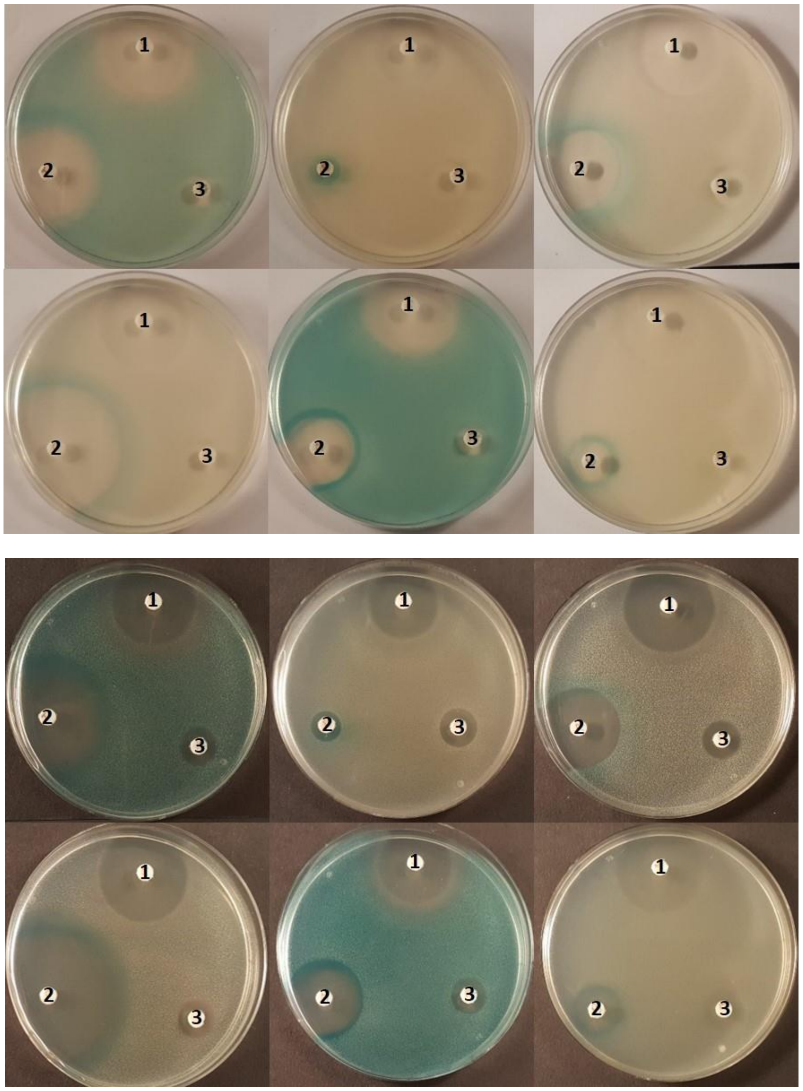

Figure S36 Reporter panel analysis of tatiomicin/tatiomicin*. Controls: **1**, (negative control) spectinomycin 1 mg; **2**, (positive controls) *fabHA* – Triclosan 250 µg, *ypuA* – cefotaxime 10 µg, *gyrA* – nalidixic acid 30 µg, *ϕ105* – doxorubicin 10 µg, *helD* – rifampicin 10 µg; **3** tatiomicin 10 µg. **Top, left to right:** fatty acid synthesis (*fabHA*), DNA damage (*ϕ105*) RNA polymerase inhibition (*helD*). **Bottom, left to right:** cell wall damage (*ypuA*), gyrase inhibition (*gyrA*), cell envelope reporter (*liaI*).

Assessment of Michael acceptor ability of tatiomicin

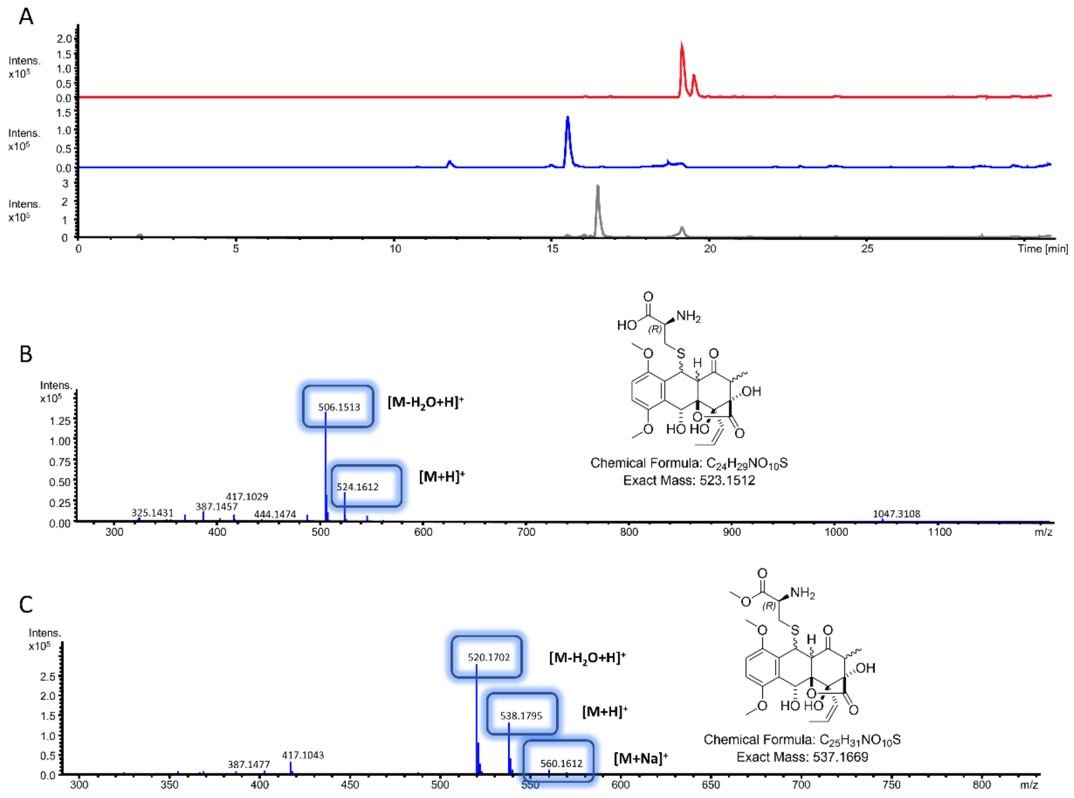

Figure S37 Michael addition of tatiomicin with L-cystine and L-cystine methyl ester. LCMS base peak chromatograms of the resulting reactions are depicted (A). Red line tatiomicin control. Blue line tatiomicin with L-cystine. Grey line tatiomicin with L-cystine methyl ester. (B) MS spectrum of L-cystine with tatiomcin at 15.5min (theoretical product shown). (C) MS spectrum of L-cystine methyl ester with tatiomcin at 16.5min (theoretical product shown). The highest isotope peak is labelled.

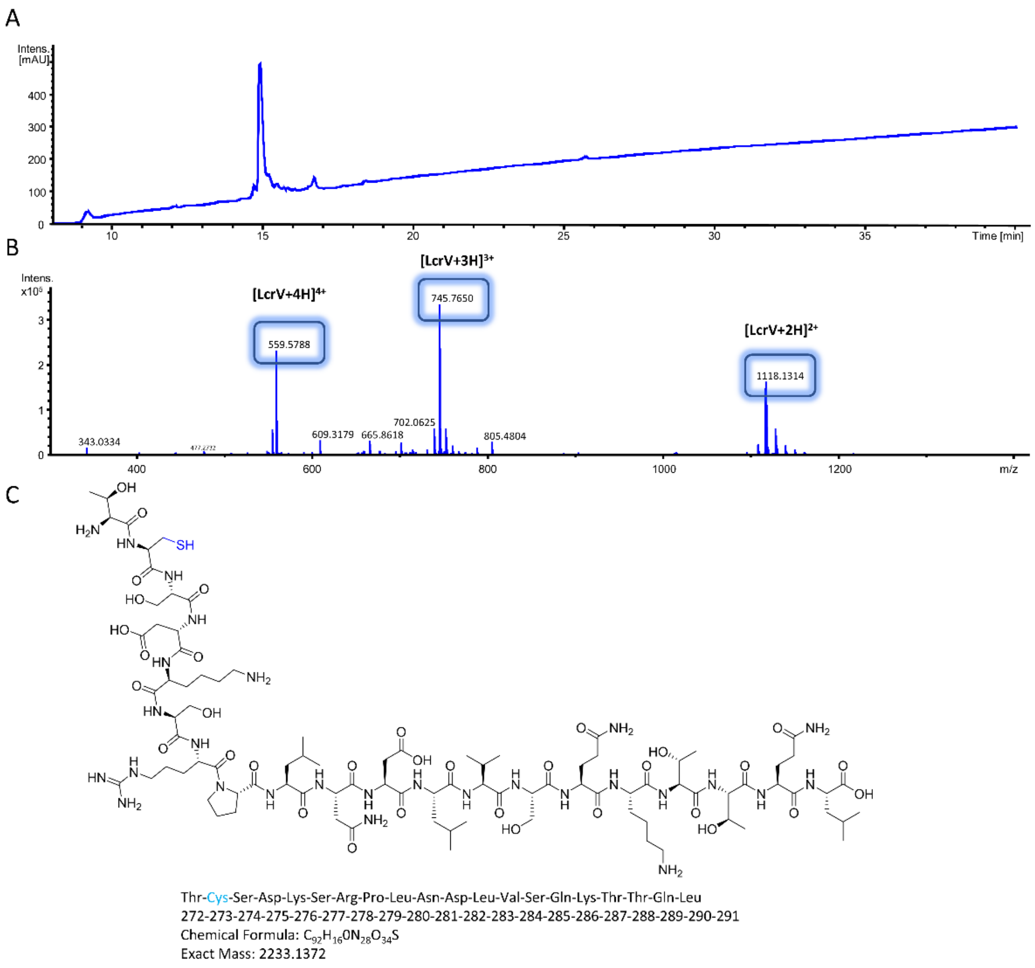

Figure S38 LCMS base peak chromatograms of the peptide LcrV (272-291) (A). MS spectrum of LcrV (272-291) at 14.9 min. The highest isotope peak is labelled. (B). Structure of LcrV (272-291) with cystine labelled in blue.

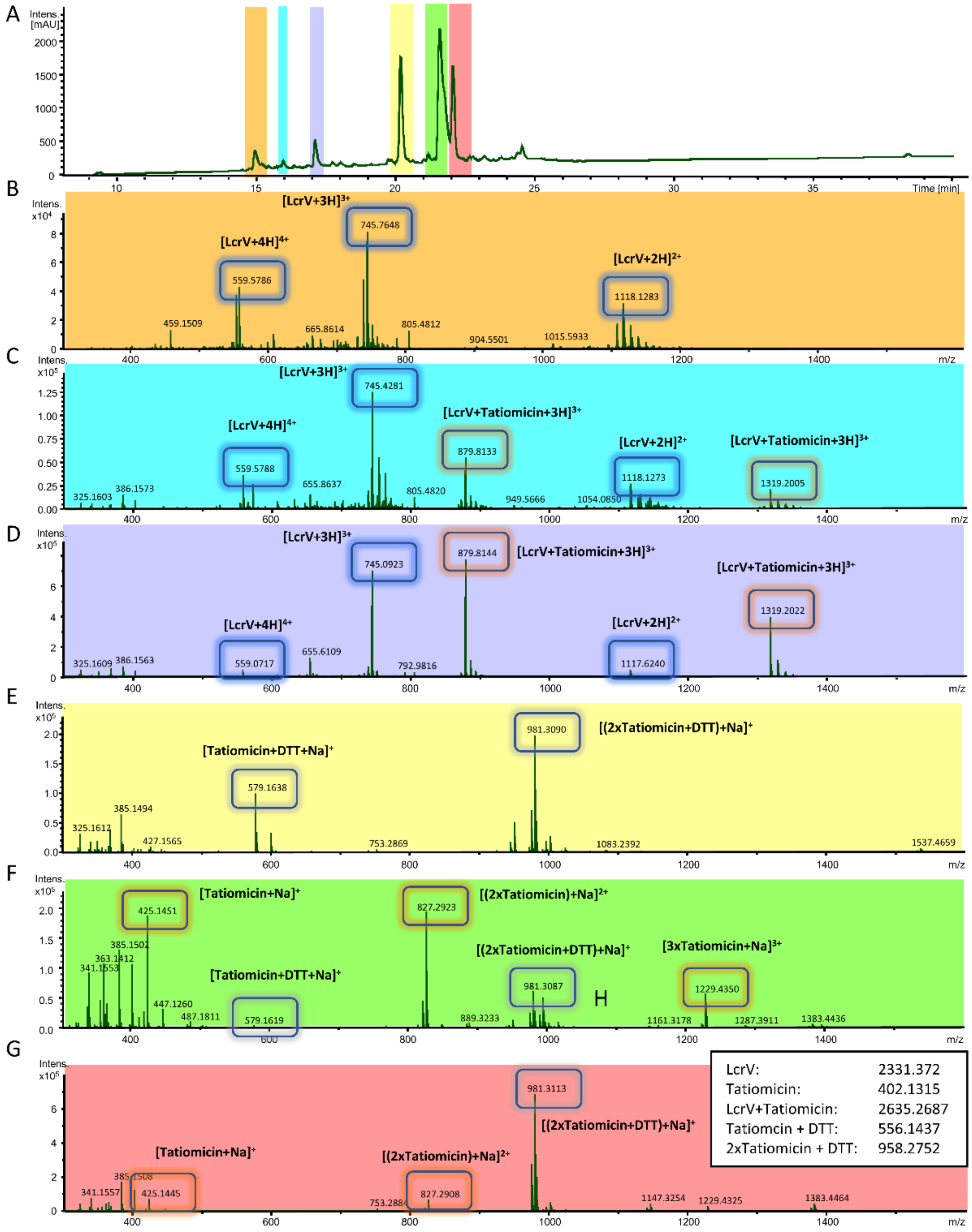

Figure S39 Michael addition of tatiomicin with LcrV (271-291) in the presence of DTT. LCMS base peak chromatograms of the resulting reactions are depicted (A) the colour of each labelled peak corresponds to MS spectrum in the same colour below. (B – orange box) Mass spectrum at 15.0 min indicates unreacted LcrV (271-291). (C – turquoise box) Mass spectrum at 16.0 min. LcrV (271-291) reacted with tatiomicin. (D – purple box) Mass spectrum at 17.2 min indicates LcrV (271-291) reacted with tatiomicin. (E – yellow box) Mass spectrum at 20.2 min. Tatiomicin reacted with DTT. (F – green box) Mass spectrum at 21.6 Tatiomicin reacted with DTT. (G – turquoise red) Mass spectrum at 22.1 min. Tatiomicin reacted with DTT. The highest isotope peak in each MS chromatogram is labelled.

Single cell microscopy

Quantification

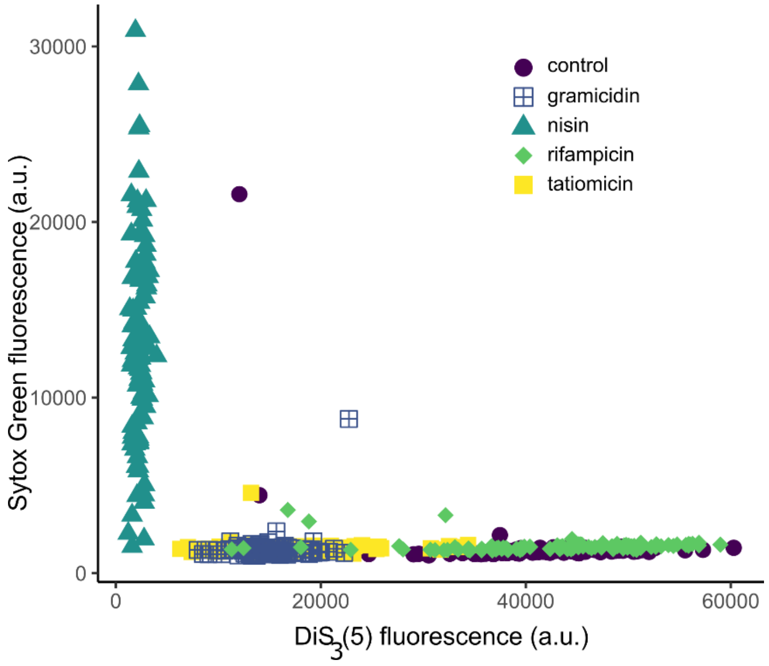

Figure S40. Cellular DiSC3(5) and Sytox Green fluorescence values were quantified for cells treated with Gramicidin, nisin, rifampicin and tatiomicin. The scatter plot depicts the fluorescence intensity values of individual cells (n = 100 per compound) for both dyes.

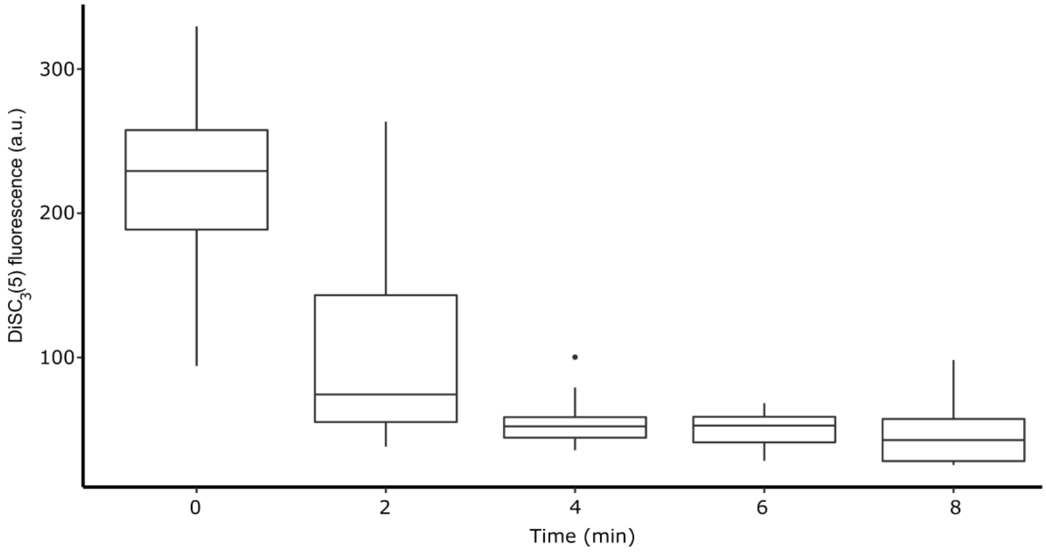

Figure S41. Cellular DiSC3(5) fluorescence values for cells treated with tatiomicin. A fresh sample was analyzed every 2 min. The box plot depicts the fluorescence intensity values of individual cells (n = >30) over time.

Creation of tatiomicin/tatiomicin* resistant mutants

B. subtilis 168CA cells were grown to late exponential phase in LB broth (~$2x10^8$ CFU/mL). We plated 100 µL ($2x10^7$ CFU) on plates containing 4x MIC (64 µg/mL) tatiomicin/tatiomicin* in duplicates and incubated the plates at 37°C for 18h. We observed four colonies. However when these were re-streaked on plates containing 64 µg/mL tatiomicin/tatiomicin* no increase in resistance could be observed.

Microorganisms used

Table S15 Microorganisms used

Strain	Reference
Amycolatopsis sp DEM30355	[19-21]
Bacillus subtilis 168CA	[22]
B. subtilis PL39 *gyrA*::pMUTIN4 *ermC gyrA'-lacZ* P_{spac}-*gyrA*$^{+}$. DNA Gyrase inhibition reporter	[19]
B. subtilis ypuA::pMUTIN4 *ermC ypuA'-lacZ.* Cell wall damage reporter	[17]
B. subtilis fabHA::pMUTIN4 *ermC gyrA'-lacZ* P_{spac}-*gyrA*$^{+}$. Fatty acid synthesis inhibition reporter (*fabHA)*	[16]
B. subtilis DNA damage reporter (*lacZ* fusion to a late promoter in a ϕ105 prophage)	[19]
B. subtilis helD::pMUTIN4 *helD-lacZ ermC.* RNA polymerase inhibition reporter	[17]
B. subtilis liaI::pMUTIN4 *liaI-lacZ ermC*. Cell envelope stress reporter	[18]
Streptomyces coelicolor M1152	[23]
Escherichia coli TOP10/pR9406	[24]
E. coli ET12567	[25]
B. subtilis trpC2 *sacA*::hbs-*mgfp* mut3 (cat)	unpublished
CRW419 *B. subtilis* trpC2 *amyE*::(spc Pxyl(M9R)-WALP23-*mcherry*(B)) *sacA*::hbs-*mgfp* mut3 (cat)	unpublished

References

1. Z. Pragai, C. Eschevins, S. Bron and C. R. Harwood, *J Bacteriol*, 2001, **183**, 2505-2515.
2. H. Nowell, S. A. Barnett, K. E. Christensen, S. J. Teat and D. R. Allan, *J Synchrotron Radiat*, 2012, **19**, 435-441.
3. G. M. Sheldrick, *Acta Crystallogr A Found Adv*, 2015, **71**, 3-8.
4. G. M. Sheldrick, *Acta Crystallogr A*, 2008, **64**, 112-122.
5. D. R. Allan, H. Nowell, S. A. Barnett, M. R. Warren, A. Wilcox, J. Christensen, L. K. Saunders, A. Peach, M. T. Hooper, L. Zaja, S. Patel, L. Cahill, R. Marshall, S. Trimnell, A. J. Foster, T. Bates, S. Lay, M. A. Williams, P. V. Hathaway, G. Winter, M. Gerstel and R. W. Wooley, *Crystals*, 2017, **7**, 336.
6. H. D. Flack, *Acta Crystallographica Section A*, 1983, **39**, 876-881.
7. H. D. Flack and G. Bernardinelli, *Acta Crystallographica Section A*, 1999, **55**, 908-915.
8. H. D. Flack and G. Bernardinelli, *Journal of Applied Crystallography*, 2000, **33**, 1143-1148.
9. *Journal*.
10. R. K. Aziz, D. Bartels, A. A. Best, M. DeJongh, T. Disz, R. A. Edwards, K. Formsma, S. Gerdes, E. M. Glass, M. Kubal, F. Meyer, G. J. Olsen, R. Olson, A. L. Osterman, R. A. Overbeek, L. K. McNeil, D. Paarmann, T. Paczian, B. Parrello, G. D. Pusch, C. Reich, R. Stevens, O. Vassieva, V. Vonstein, A. Wilke and O. Zagnitko, *BMC Genomics*, 2008, **9**, 75.
11. K. Blin, S. Shaw, A. M. Kloosterman, Z. Charlop-Powers, G. P. van Wezel, Marnix H. Medema and T. Weber, *Nucleic Acids Research*, 2021, **49**, W29-W35.
12. X. Yan, K. Probst, A. Linnenbrink, M. Arnold, T. Paululat, A. Zeeck and A. Bechthold, *Chembiochem*, 2012, **13**, 224-230.
13. P. Schwarzer, O. Tsypik, C. Zuo, A. Alali, J. Wunsch-Palasis, T. Heitzler, J. Derochefort, M. Bernhardt, X. Yan, T. Paululat and A. Bechthold, *Molecules*, 2020, **25**, 1955.
14. A. Alali, L. Zhang, J. Li, C. Zuo, D. Wassouf, X. Yan, P. Schwarzer, S. Günther, O. Einsle and A. Bechthold, *Molecular Biotechnology*, 2021, **63**, 502-514.
15. O. Tsypik, R. Makitrynskyy, B. Frensch, D. L. Zechel, T. Paululat, R. Teufel and A. Bechthold, *J Am Chem Soc*, 2020, **142**, 5913-5917.
16. H. P. Fischer, N. A. Brunner, B. Wieland, J. Paquette, L. Macko, K. Ziegelbauer and C. Freiberg, *Genome Res*, 2004, **14**, 90-98.
17. A. Urban, S. Eckermann, B. Fast, S. Metzger, M. Gehling, K. Ziegelbauer, H. Rubsamen-Waigmann and C. Freiberg, *Appl Environ Microbiol*, 2007, **73**, 6436-6443.
18. T. Mascher, S. L. Zimmer, T. A. Smith and J. D. Helmann, *Antimicrob Agents Chemother*, 2004, **48**, 2888-2896.
19. B. Kepplinger, S. Morton-Laing, K. H. Seistrup, E. C. L. Marrs, A. P. Hopkins, J. D. Perry, H. Strahl, M. J. Hall, J. Errington and N. E. E. Allenby, *ACS Chem Biol*, 2018, **13**, 207-214.
20. H. Mosaei, V. Molodtsov, B. Kepplinger, J. Harbottle, C. W. Moon, R. E. Jeeves, L. Ceccaroni, Y. Shin, S. Morton-Laing, E. C. L. Marrs, C. Wills, W. Clegg, Y. Yuzenkova, J. D. Perry, J. Bacon, J. Errington, N. E. E. Allenby, M. J. Hall, K. S. Murakami and N. Zenkin, *Mol Cell*, 2018, **72**, 263-274 e265.
21. C. K. Okoro, R. Brown, A. L. Jones, B. A. Andrews, J. A. Asenjo, M. Goodfellow and A. T. Bull, *Antonie Van Leeuwenhoek*, 2009, **95**, 121-133.
22. F. Kunst, N. Ogasawara, I. Moszer, A. M. Albertini, G. Alloni, V. Azevedo, M. G. Bertero, P. Bessières, A. Bolotin, S. Borchert, R. Borriss, L. Boursier, A. Brans, M. Braun, S. C. Brignell, S. Bron, S. Brouillet, C. V. Bruschi, B. Caldwell, V. Capuano, N. M. Carter, S. K. Choi, J. J. Cordani, I. F. Connerton, N. J. Cummings, R. A. Daniel, F. Denziot, K. M. Devine, A. Düsterhöft, S. D. Ehrlich, P. T. Emmerson, K. D. Entian, J. Errington, C. Fabret, E. Ferrari, D. Foulger, C. Fritz, M. Fujita, Y.

Fujita, S. Fuma, A. Galizzi, N. Galleron, S. Y. Ghim, P. Glaser, A. Goffeau, E. J. Golightly, G. Grandi, G. Guiseppi, B. J. Guy, K. Haga, J. Haiech, C. R. Harwood, A. Hènaut, H. Hilbert, S. Holsappel, S. Hosono, M. F. Hullo, M. Itaya, L. Jones, B. Joris, D. Karamata, Y. Kasahara, M. Klaerr-Blanchard, C. Klein, Y. Kobayashi, P. Koetter, G. Koningstein, S. Krogh, M. Kumano, K. Kurita, A. Lapidus, S. Lardinois, J. Lauber, V. Lazarevic, S. M. Lee, A. Levine, H. Liu, S. Masuda, C. Mauël, C. Médigue, N. Medina, R. P. Mellado, M. Mizuno, D. Moestl, S. Nakai, M. Noback, D. Noone, M. O'Reilly, K. Ogawa, A. Ogiwara, B. Oudega, S. H. Park, V. Parro, T. M. Pohl, D. Portelle, S. Porwollik, A. M. Prescott, E. Presecan, P. Pujic, B. Purnelle, G. Rapoport, M. Rey, S. Reynolds, M. Rieger, C. Rivolta, E. Rocha, B. Roche, M. Rose, Y. Sadaie, T. Sato, E. Scanlan, S. Schleich, R. Schroeter, F. Scoffone, J. Sekiguchi, A. Sekowska, S. J. Seror, P. Serror, B. S. Shin, B. Soldo, A. Sorokin, E. Tacconi, T. Takagi, H. Takahashi, K. Takemaru, M. Takeuchi, A. Tamakoshi, T. Tanaka, P. Terpstra, A. Togoni, V. Tosato, S. Uchiyama, M. Vandebol, F. Vannier, A. Vassarotti, A. Viari, R. Wambutt, H. Wedler, T. Weitzenegger, P. Winters, A. Wipat, H. Yamamoto, K. Yamane, K. Yasumoto, K. Yata, K. Yoshida, H. F. Yoshikawa, E. Zumstein, H. Yoshikawa and A. Danchin, *Nature*, 1997, **390**, 249-256.

23. A. Thanapipatsiri, J. Claesen, J. P. Gomez-Escribano, M. Bibb and A. Thamchaipenet, *Microb Cell Fact*, 2015, **14**, 145.
24. A. C. Jones, B. Gust, A. Kulik, L. Heide, M. J. Buttner and M. J. Bibb, *PLoS One*, 2013, **8**, e69319.
25. D. J. MacNeil, K. M. Gewain, C. L. Ruby, G. Dezeny, P. H. Gibbons and T. MacNeil, *Gene*, 1992, **111**, 61-68.
